# UDP-glucosyltransferase regulates grain size and abiotic stress tolerance associated with metabolic flux redirection in rice

**DOI:** 10.1038/s41467-020-16403-5

**Published:** 2020-05-26

**Authors:** Nai-Qian Dong, Yuwei Sun, Tao Guo, Chuan-Lin Shi, Yi-Min Zhang, Yi Kan, You-Huang Xiang, Hai Zhang, Yi-Bing Yang, Ya-Chao Li, Huai-Yu Zhao, Hong-Xiao Yu, Zi-Qi Lu, Yong Wang, Wang-Wei Ye, Jun-Xiang Shan, Hong-Xuan Lin

**Affiliations:** 1 National Key Laboratory of Plant Molecular Genetics, CAS Centre for Excellence in Molecular Plant Sciences and Collaborative Innovation Center of Genetics & Development, Shanghai Institute of Plant Physiology & Ecology, Chinese Academic of Sciences, Shanghai, 200032 China; 2 CAS-Key Laboratory of Synthetic Biology, CAS Center for Excellence in Molecular Plant Sciences, Shanghai Institute of Plant Physiology & Ecology, Chinese Academy of Sciences, Shanghai, 200032 China; 3 University of the Chinese Academy of Sciences, 100049 Beijing, China; 4 School of Life Science and Technology, ShanghaiTech University, Shanghai, 201210 China

**Keywords:** Agricultural genetics, Plant breeding, Secondary metabolism

## Abstract

Grain size is an important component trait of grain yield, which is frequently threatened by abiotic stress. However, little is known about how grain yield and abiotic stress tolerance are regulated. Here, we characterize *GSA1*, a quantitative trait locus (QTL) regulating grain size and abiotic stress tolerance associated with metabolic flux redirection. *GSA1* encodes a UDP-glucosyltransferase, which exhibits glucosyltransferase activity toward flavonoids and monolignols. *GSA1* regulates grain size by modulating cell proliferation and expansion, which are regulated by flavonoid-mediated auxin levels and related gene expression. GSA1 is required for the redirection of metabolic flux from lignin biosynthesis to flavonoid biosynthesis under abiotic stress and the accumulation of flavonoid glycosides, which protect rice against abiotic stress. *GSA1* overexpression results in larger grains and enhanced abiotic stress tolerance. Our findings provide insights into the regulation of grain size and abiotic stress tolerance associated with metabolic flux redirection and a potential means to improve crops.

## Abstract (web summary)

Increasing grain yield needs to be put in the context of environmental stress. Here, the authors reveal that a UDP-glucosyltransferase is associated with regulation of rice grain size, abiotic stress tolerance, flavonoid-mediated auxin signaling, and redirection of carbon flux to flavonoid glycosides synthesis.

## Introduction

Rice is one of the most important crops, providing food for more than half of the world’s population. Rice yield is mainly determined by quantitative traits such as effective panicle number, grain number per panicle, and grain weight. Mutant identification and map-based cloning of quantitative trait loci (QTLs) have widened our knowledge of the genetic and molecular bases of these agronomic traits^1,2^. Among them, grain weight is largely determined by grain size^3^. In recent decades, a large number of QTLs have been identified and functionally characterized in rice^4–19^, regulating grain size through signaling pathways mediated by phytohormones, G-proteins, proteasomal degradation, protein kinases, and transcriptional factors^1,2^. Nevertheless, little is known about QTLs synergistically regulating grain size and abiotic stress.

As sessile organisms, plants have evolved to cope with diverse abiotic stresses such as soil salinity, drought, and extreme temperatures, which affect plant development and threaten crop yields. Excessive reactive oxygen species (ROS) production is induced by abiotic stress and results in oxidative damage to proteins, DNA, and membrane lipids. ROS can be scavenged by antioxidants, such as ascorbic acid, glutathione, flavonoids, carotenoids, and proline^20^. Flavonoids accumulate in response to different abiotic stresses and function as antioxidants to reduce oxidative damage^21–25^. Glycosylation catalyzed by glycosyltransferases plays an essential role in regulating the stability, availability, and biological activity of flavonoids including anthocyanins^26,27^. To date, several QTLs associated with abiotic stress, such as *SKC1*^28^, *COLD1*^29^, and *TT1*^30^, have been molecularly identified and characterized in rice. However, QTLs controlling abiotic stress tolerance by regulating flavonoid glycosylation remain largely unknown.

Here, we report the identification and characterization of a QTL, *Grain Size* *and* *Abiotic stress tolerance 1* (*GSA1*), which encodes a UDP-glucosyltransferase (UTG83A1). Natural variations in *GSA1* result in smaller grains due to flavonoid-mediated affection of auxin levels, PIN1 protein levels and auxin-related gene expression. GSA1 catalyzes glucosylation of monolignols and flavonoids and modulates the redirection of metabolic flux by altering flavonoid glycoside profiles and the phenylpropanoid pathway in response to abiotic stress. Collectively, we clone and characterize a rice QTL and reveal possible mechanism underlying the regulation of grain size and abiotic stress tolerance. The knowledge will pave the way for improving crop yields and abiotic stress resistance.

## Results

### GSA1 is a QTL for grain size

To identify QTLs underlying grain yield, we constructed a set of CSSLs with an African rice variety, CG14 (*Oryza. glaberrima*), as the donor parent and an Asian rice variety, Wuyunjing (WYJ, *Oryza. sativa japonica*) as the recurrent parent^31^. Multiple QTLs for grain size were identified, and we selected a QTL on chromosome 3, designated *GSA1*, for further characterization. *GSA1* is a QTL for grain size, and explains 14.5% of the phenotypic variation for 1000-grain weight, 18.6% of the phenotypic variation for grain length and 14.1% of the phenotypic variation for grain width (Supplementary Table 1). *GSA1* has an additive and semi-dominant effect on grain size (Supplementary Fig. 1a–c).

**Fig. 1:**
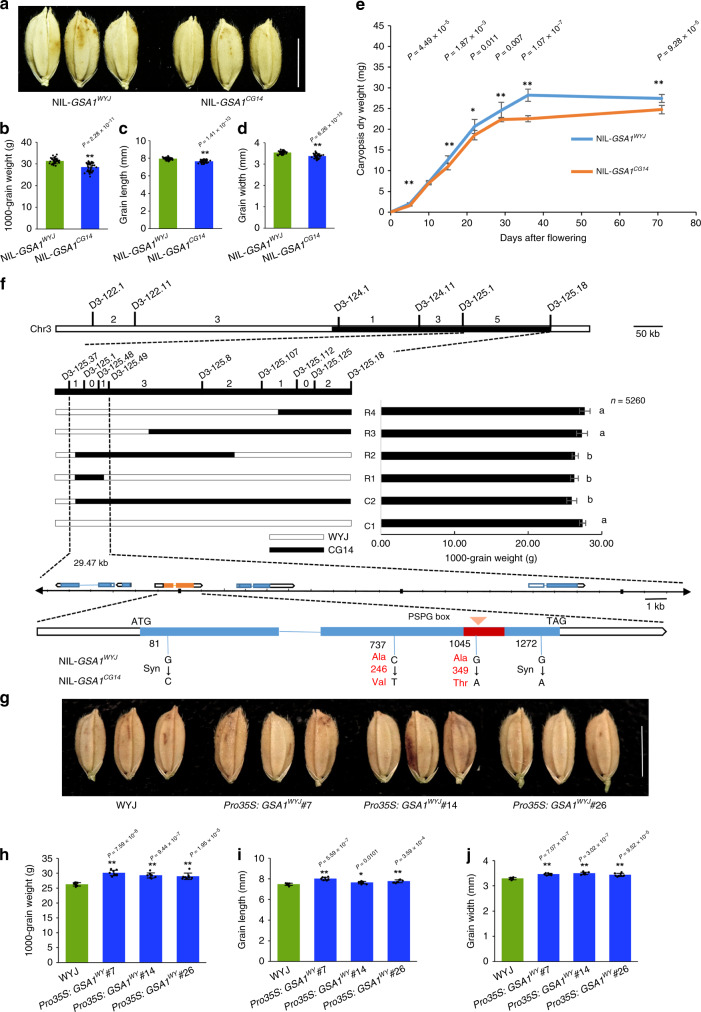
Map-based Cloning of *GSA1*. **a** Mature grains of NIL-*GSA1*^*WYJ*^ and NIL-*GSA1*^*CG14*^. Scale bar = 5 mm. Comparison of 1000-grain weight (**b**), grain length (**c**) and grain width (**d**) between NIL-*GSA1*^*WYJ*^ and NIL-*GSA1*^*CG14*^ (*n* = 40 plants). **e** Time-course of the change in caryopsis dry weight for NIL-*GSA1*^*WYJ*^ and NIL-*GSA1*^*CG14*^ (*n* = 8 plants, 40 caryopses per plant). **f** *GSA1* was initially mapped to the interval between the markers D3-125.1 and D3-125.18 on long arm of chromosome 3 and then narrowed to a 29.47 kb region containing five genes. The numbers of recombinant individuals are shown between the marker positions. 1000-grain weight is shown to the right of the schematic for each representative recombinant line. Recombinant lines R2, R3, and R4 originated from recombinant line C2; recombinant line R1 originated from recombinant line R2. Values represent the mean ± s.d. (*n* = 20 plants). Different letters indicate significant differences (*P* < 0.05) determined by Duncan’s multiple range test. Single nucleotide mutations and the corresponding amino acid changes in NIL-*GSA1*^*CG14*^ are shown beneath the schematic illustration of the *GSA1* gene. Syn, synonymous variations. The red box indicates the PSPG domain. **g** Mature grains of WYJ and the overexpression lines *Pro35S: GSA1*^*WYJ*^#7, *Pro35S:GSA1*^*WYJ*^#14 and *Pro35S:GSA1*^*WYJ*^#26. Scale bar = 5 mm. Comparison of 1000-grain weight (**h**), grain length (**i**) and grain width (**j**) between WYJ and the overexpression lines (*n* = 8 plants). The values in **b**–**e** and **h**–**j** represent the mean ± s.d. *P < 0.05 and **P < 0.01 indicate significant differences compared with NIL-*GSA1*^*WYJ*^ or WYJ in two-tailed Student’s *t* tests. The source data underlying Fig. 1b–f and h–j are provided as Source Data file.

We also constructed a nearly isogenic line (NIL) of *GSA1*, NIL-*GSA1*^*CG14*^, and an isogenic control, NIL-*GSA1*^*WYJ*^, to further investigate the effects of the *GSA1* locus on grain size and other agronomic traits. NIL-*GSA1*^*CG14*^ exhibited a decrease in 1000-grain weight (−9.29%), grain length (−3.78%), and grain width (−4.8%) compared with NIL-*GSA1*^*WYJ*^ (Fig. 1a–d), and NIL-*GSA1*^*CG14*^ produced more grains compared with NIL-*GSA1*^*WYJ*^ (Supplementary Fig. 1d). However, no significant difference was observed in plant height, number of effective panicles, panicle length or grain yield per plant (Supplementary Fig. 1e–j). These results demonstrate that *GSA1* is a QTL contributing to grain size.

Given the smaller grains in NIL-*GSA1*^*CG14*^, we investigated the grain milk filling rate in NIL-*GSA1*^*WYJ*^ and NIL-*GSA1*^*CG14*^. The fresh weight, dry weight, length, and width of caryopses from NIL-*GSA1*^*CG14*^ were significantly smaller than those of NIL-*GSA1*^*WYJ*^ (Fig. 1e and Supplementary Fig. 2a–d). There was no significant difference in caryopsis water content between NIL-*GSA1*^*WYJ*^ and NIL-*GSA1*^*CG14*^ during caryopsis development (Supplementary Fig. 2e), indicating that *GSA1* is involved in regulating dry matter accumulation and caryopsis development.

To clone the causal gene for *GSA1*, we performed high-resolution mapping and narrowed the *GSA1* locus to a 29.47-kb region (Fig. 1f and Supplementary Fig. 3). In this region, five genes were annotated, namely three UDP-glucosyltransferase (UGT) encoding genes *LOC_Os03g55030*, *LOC_Os03g55040*, and *LOC_Os03g55050*, a UDP-glucose-6-dehydrogenase encoding gene *LOC_Os03g55070* which had a synonymous nucleotide substitution, and a small peptide*-*encoding gene, *LOC_Os03g55034*, with an unknown function. Sequence comparison among the UDP-glucosyltransferase encoding genes identified a nucleotide variation between the two parental lines causing an amino acid substitution from alanine to threonine at position 349 (A349T) within the conserved Plant Secondary Product Glycosyltransferase (PSPG) box domain in *LOC_Os03g55040*, and two synonymous nucleotide substitutions and another nucleotide variation resulting in an amino acid substitution (A246V) were also identified (Fig. 1f and Supplementary Fig. 4a). Further protein sequence alignment revealed that A349 is conserved in most monocots and that *GSA1*^*CG14*^ is a rare allele of *GSA1* (Supplementary Fig. 4b). Phylogenetic analysis of *GSA1* and its homologs in *Gramineae*, *Brassicaceae Burnett* and *Leguminosae* species revealed that GSA1 sequences from monocotyledons and dicotyledons were separated into two branches, which suggested that *GSA1* had existed before monocotyledons and dicotyledons diverged during plant evolution, and that *GSA1* is a conserved gene with a fundamental function (Supplementary Fig. 4c). In addition, we compared the promoter regions of *GSA1*^*WYJ*^ and *GSA1*^*CG14*^ and found natural variations in the conserved motifs of the *GSA1*^*CG14*^ promoter region predicted by the PlantCARE database^32^; these natural variations may affect the binding of transcription factor to these motifs and the activation of the *GSA1* promoter (Supplementary Fig. 5). Therefore, *LOC_Os03g55040* is the most likely candidate gene for the *GSA1* locus.

We then analysed the nucleotide diversity and selection signatures in *GSA1* in African and Asian rice. We examined the nucleotide diversity of a ~3.3-kb genomic region containing the promoter (~1 kb) and the entire ORF (~1.6 kb) of *GSA1* using Rice3K data for *O. sativa ssp. japonica* and *O. sativa ssp. indica* varieties^33^, and data for 446 *O. rufipogon* accessions^34^, 20 *O. glaberrima* varieties, and 94 *O. barthii* accessions^35^. A sliding-window analysis of the nucleotide diversity in this 3.3-kb region showed that the π value in the *O. glaberrima* varieties was much lower than in *O. barthii* accessions (Supplementary Fig. 6a). The π value in *O. sativa ssp. japonica* varieties were much lower than in *O. rufipogon* accessions (Supplementary Fig. 6b), while the π value in *O. sativa ssp. indica* varieties were much higher than in *O. rufipogon* (Supplementary Fig. 6c), implying that more natural variations occurred during the domestication of *O. sativa ssp. indica* varieties from *O. rufipogon*. These results demonstrated that *GSA1* has been directionally selected in *O. glaberrima* and *O. sativa ssp. japonica* varieties during the domestication of African and Asian rice.

To further verify that *LOC_Os03g55040* corresponds to *GSA1*, we overexpressed *GSA1*^*WYJ*^ in the WYJ background and subsequently observed an obvious increase in 1000-grain weight (+12.06%), grain length (+4.41%) and grain width (+5.27%) in the positive transgenic lines (Fig. 1g–j and Supplementary Fig. 7a). In contrast, knocking out *GSA1*^*WYJ*^ using the CRISPR/Cas9 system and overexpressing *GSA1*^*CG14*^ in the WYJ background resulted in smaller grains compared with WYJ (Supplementary Fig. 7b–j and Supplementary Table 2**)**. We then performed a genetic complementation test in which a DNA fragment from WYJ containing the putative promoter region, the entire ORF, and the 3′ untranslated region of *GSA1* was introduced into NIL-*GSA1*^*CG14*^. The transgenic line harboring the full-length *GSA1* transgene showed NIL-*GSA1*^*WYJ*^ phenotypes with respect to 1000-grain weight, grain length, and grain width (Supplementary Fig. 7k–n). In addition, knock-out lines of two other UGT genes, *LOC_Os03g55030* and *LOC_Os03g55050*, generated using the CRISPR/Cas9 system exhibited no significant difference compared with WYJ with respect to 1000-grain weight, grain length, and grain width (Supplementary Fig. 8a–j). Moreover, the expression levels of *LOC_Os03g55030* and *LOC_Os03g55050* were not significantly different between NIL-*GSA1*^*WYJ*^ and NIL-*GSA1*^*CG14*^ (Supplementary Fig. 8k). These results imply that the other UGT genes within the *GSA1* target region have no effect on grain size. Taken together, the above-mentioned results indicate that *GSA1* is a positive regulator of grain size and confirm that we successfully cloned *GSA1*, a QTL that finely regulates grain size in rice.

### GSA1 regulates cell proliferation and cell expansion

**Fig. 2:**
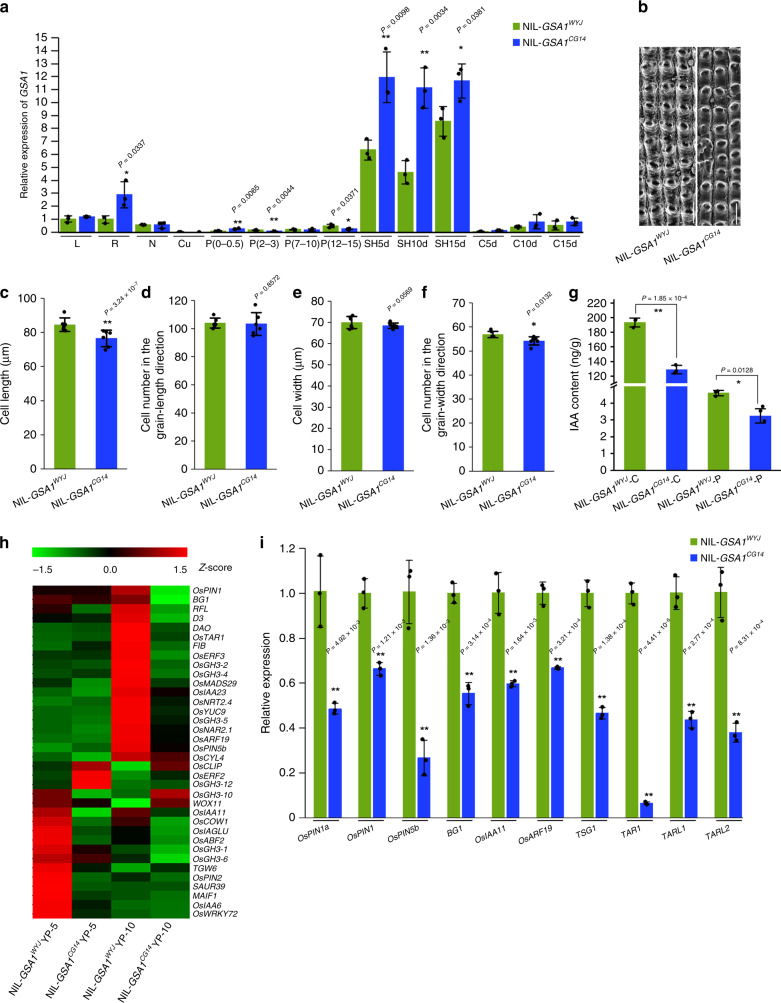
*GSA1* controls spikelet size by influencing cell proliferation and cell expansion. **a** The relative expression levels of *GSA1* in NIL-*GSA1*^*WYJ*^ and NIL-*GSA1*^*CG14*^ leaves (L), roots (R), culm nodes (N), culms (Cu), young panicles (P, numbers in brackets indicate the length of young panicles, cm), spikelet hulls (SH) and caryopses (C) determined by qRT-PCR (*n* = 3 biological replicates). 5 d, 10 d, and 15 d indicate 5 days, 10 days and 15 days after flowering, respectively. The actin gene was used for normalization. **b** Scanning electron micrographs of the outer epidermal cells of NIL-*GSA1*^*WYJ*^ and NIL-*GSA1*^*CG14*^ spikelet hulls at the mature stage. Scale bar, 100 μm. Comparison of the outer epidermal cell length (**c**), cell number in the grain-length direction (**d**), cell width (**e**) and cell number in the grain-width direction (**f**) of spikelet hulls between NIL-*GSA1*^*WYJ*^ and NIL-*GSA1*^*CG14*^ (*n* = 6 grains). **g** Comparison of the endogenous IAA levels in young caryopses (C, 10 days after flowering) and young panicles (P, ~10 cm) at the booting stage between NIL-*GSA1*^*WYJ*^ and NIL-*GSA1*^*CG14*^ (*n* = 3 biological replicates). **h** Clustering heat maps of the relative expression levels of auxin-related genes in young panicles (YP) of NIL-*GS3.2*^*WYJ*^ and NIL-*GS3.2*^*CG14*^ determined using RNA-seq data. YP-5, 5 cm young panicles. YP-10, 10 cm young panicles. Standard-scores (Z-scores) were used as the numerical signs to evaluate the standard deviations from the mean of the corresponding samples. **i** The relative expression levels of auxin-related genes in young panicles (~10 cm) of NIL-*GSA1*^*WYJ*^ and NIL-*GSA1*^*CG14*^ determined by qRT-PCR (*n* = 3 biological replicates). The values in **a**, **c**–**g**, and **i** represent the mean ± s.d. *P < 0.05 and **P < 0.01 indicate significant differences compared with NIL-*GSA1*^*WYJ*^ in two-tailed Student’s *t* tests. The ubiquitin gene was used for normalization. The source data underlying Fig. 2a and c–i are provided as Source Data file.

We examined the tissue-specific expression pattern of *GSA1* and found that *GSA1* was widely expressed in various reproductive and vegetative organs, and notably was expressed at a relatively high level in spikelet hulls and caryopses, consistent with the role in controlling spikelet development. Furthermore, the expression level of *GSA1* in NIL-*GSA1*^*CG14*^ was relatively higher than that in NIL-*GSA1*^*WYJ*^, especially in spikelet hulls (Fig. 2a and Supplementary Fig. 9a). Hence, we speculate that *GSA1*^*CG14*^ is a weakly functioning allele that results in higher *GSA1* expression level through a feedback loop. In addition, conserved motifs containing natural variations in *GSA1*^*CG14*^ promoter region (Supplementary Fig. 5) may also possibly affect the expression of *GSA1*.

Because NIL-*GSA1*^*CG14*^ exhibits smaller grains, we investigated the cell number and cell size in mature and young spikelets. Cell length and cell number in the grain-width direction of outer epidermal cells were significantly reduced in mature grains of NIL-*GSA1*^*CG14*^ compared with those of NIL-*GSA1*^*WYJ*^, and cell width and cell number in the grain-length direction were slightly decreased (Fig. 2b–f and Supplementary Fig. 9b). In agreement with this observation, NIL-*GSA1*^*CG14*^ formed smaller young spikelets with reduced cell width and cell number in grain-width direction (Supplementary Fig. 9c–j). We also compared cross sections of the central parts of the spikelet hulls in NIL-*GSA1*^*WYJ*^ and NIL-*GSA1*^*CG14*^ at the booting stage. Our observations revealed that there were significantly fewer outer parenchyma cells in NIL-*GSA1*^*CG14*^ than in NIL-*GSA1*^*WYJ*^ (Supplementary Fig. 9k, l). Observation of inner epidermal cells revealed that the average length and width of cells in NIL-*GSA1*^*CG14*^ spikelet hulls was significantly decreased compared with those in NIL-*GSA1*^*WYJ*^ (Supplementary Fig. 9m–o). These results suggest that *GSA1* is a QTL that finely regulates spikelet development by controlling cell proliferation and cell expansion.

Plant growth and development are largely influenced by auxin, and a recent study revealed that the disruption of local auxin biosynthesis in rice affects auxin signaling and transport, as well as localized cell proliferation and cell expansion, resulting in smaller grains^36^. Given that *GSA1* is responsible for cell proliferation and cell expansion and regulates spikelet development, we asked whether local auxin biosynthesis, signaling and transport are disrupted in NIL-*GSA*^*CG14*^. We measured the endogenous auxin level in young caryopses and young panicles of NIL-*GSA1*^*WYJ*^ and NIL-*GSA1*^*CG14*^ at the booting stage. The IAA levels in young NIL-*GSA1*^*CG14*^ caryopses and panicles were remarkably lower than those in NIL-*GSA1*^*WYJ*^ (Fig. 2g), and the same decrease was also observed for indole-3-carboxaldehyde (ICA, photooxidation product of IAA, Supplementary Fig. 9p), suggesting a reduced amount of auxin biosynthesis in NIL-*GSA1*^*CG14*^ compared with NIL-*GSA1*^*WYJ*^ during spikelet development. Moreover, RNA-seq data showed that the expression levels of a large number of genes involved in auxin biosynthesis, signaling, transport, and metabolism were significantly decreased in the young panicles of NIL-*GSA1*^*CG14*^ compared with NIL-*GSA1*^*WYJ*^ (Fig. 2h). In order to validate the RNA-seq results, we performed qRT-PCR assays using young panicles of NIL-*GSA1*^*WYJ*^ and NIL-*GSA1*^*CG14*^. We found that the relative expression levels of genes encoding IAA biosynthetic components, auxin transport proteins and auxin response factors were obviously lower in NIL-*GSA1*^*CG14*^ than in NIL-*GSA1*^*WYJ*^ (Fig. 2i). Interestingly, the expression level of *TSG1*, which controls grain size by regulating local auxin biosynthesis^36^, was significantly decreased in NIL-*GSA1*^*CG14*^; the expression levels of the *TSG1* homologs *OsTAR1*, *OsTARL1*, and *OsTARL2* were also decreased (Fig. 2i). In addition, the expression levels of auxin-related genes in seedlings of the complementary line *gGSA1*^*com*^ were comparable to that of NIL-*GSA1*^*WYJ*^ (Supplementary Fig. 9q). Moreover, the endogenous OsPIN1 protein level was clearly lower in young panicles of NIL-*GSA1*^*CG14*^ than in that of NIL-*GSA1*^*WYJ*^ (Supplementary Fig. 9r). Thus, we conclude that *GSA1* takes part in the regulation of cell proliferation and cell expansion underlying spikelet development and also indirectly affects auxin levels, PIN1 protein levels and auxin-related gene expression which may be responsible for the differences in grain size.

### GSA1 modulates phenylpropanoid homeostasis

**Fig. 3:**
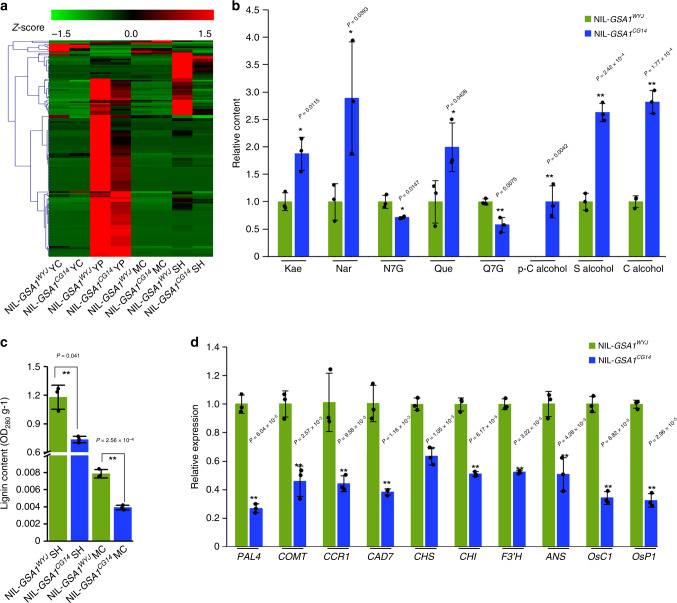
Altered flavonoid glycoside profiles and phenylpropanoid pathway in NIL-*GSA1*^*CG14*^. **a** Clustering heat maps of the relative levels of flavonoid glycosides in young caryopses (YC, 10 days after flowering), young panicles (YP, ~10 cm) at the booting stage, mature caryopses (MC) and mature spikelet hulls (SH) of NIL-*GSA1*^*WYJ*^ and NIL-*GSA1*^*CG14*^ (*n* = 3 biological replicates) from widely targeted metabolomics data. Standard-scores (Z-scores) were used as the numerical signs to evaluate the standard deviations from the mean of the corresponding samples. **b** Relative levels of flavonoids and flavonoid glycosides (Kae, Nar, N7G, Que, Q7G) in young panicles and monolignol (p-C alcohol, S alcohol, C alcohol) in young caryopses of NIL-*GSA1*^*WYJ*^ and NIL-*GSA1*^*CG14*^ (*n* = 3 biological replicates). Kae, kaempferol. Nar, naringenin. N7G, naringenin-7-*O*-glucoside. Que, quercetin. Q7G, quercetin-7-*O*-glucoside. p-C alcohol, p-coumaryl alcohol. S alcohol, sinapyl alcohol. C alcohol, coniferyl alcohol. **c** The lignin content of mature spikelet hulls (SH) and mature caryopses (MC) of NIL-*GSA1*^*WYJ*^ and NIL-*GSA1*^*CG14*^ (*n* = 3 biological replicates, 5 plants per replicate). **d** The relative expression levels of genes involved in the general phenylpropanoid pathway (*PAL4*, *COMT*), the lignin specific pathway (*CCR1*, *CAD7*), flavonoid biosynthesis (*CHS*, *CHI*, *F3’H*) and anthocyanin biosynthesis (*ANS*, *OsC1*, *OsP1*) determined by qRT-PCR in young panicles (10 cm) of NIL-*GSA1*^*WYJ*^ and NIL-*GSA1*^*CG14*^ at the booting stage (*n* = 3 biological replicates). The ubiquitin gene was used for normalization. The values in **b**–**d** represent the mean ± s.d. *P < 0.05 and **P < 0.01 indicate significant differences compared with NIL-*GSA1*^*WYJ*^ in two-tailed Student’s *t* tests. Source data are provided as Source Data file.

The phenylpropanoid pathway produces enormous amounts of secondary metabolites, such as monolignols and flavonoids^37,38^. The *GSA1* gene is predicted to encode a UDP-glucosyltransferase (UGT83A1) containing a PSPG box domain. Uridine diphosphate (UDP) glycosyltransferases (UGTs) catalyze the transfer of activated sugar linked to UDP to acceptors such as lipids, proteins and secondary metabolites, regulating their bioactivity, solubility and stability^26,39^. To further investigate the roles and potential sugar acceptors of *GSA1*, we performed a widely targeted metabolomics assay in various tissues. As shown in Fig. 3a, the relative levels of flavonoid glycosides were significantly lower in young panicles, spikelet hulls, and young caryopses of NIL-*GSA1*^*CG14*^ than in those of NIL-*GSA*^*WYJ*^, indicating that flavonoid glycoside biosynthesis was disrupted in NIL-*GSA1*^*CG14*^. In addition, levels of aglycone flavonoids such as kaempferol, naringenin, and quercetin were clearly higher in NIL-*GSA1*^*CG14*^ panicles, while the levels of flavonoid glycosides such as naringenin-7-*O*-glucoside and quercetin-7-*O*-glucoside were significantly lower (Fig. 3b), implying that *GSA1* is required for the biosynthesis of flavonoid glycosides. Moreover, levels of monolignols (p-coumaryl alcohol, sinapyl alcohol and coniferyl alcohol) were higher in young caryopses of NIL-*GSA1*^*CG14*^ than in those of NIL-*GSA1*^*WYJ*^ (Fig. 3b), whereas the contents of lignin, a polymer of heterogeneous monolignols, in spikelet hulls and mature caryopses were notably reduced compared with those in NIL-*GSA1*^*WYJ*^ (Fig. 3c), indicating that *GSA1* also contributes to lignin biosynthesis. Then we assayed the expression levels of genes related to the phenylpropanoid pathway. As shown in Fig. 3d, the expression levels of genes involved in the central phenylpropanoid pathway (*PAL4*, *COMT*), the lignin pathway (*CCR1*, *CAD7*), flavonoid biosynthesis (*CHS*, *CHI*, *F3’H*) and anthocyanin biosynthesis (*ANS*, *OsC1*, *OsP1*) were obviously decreased in young panicles of NIL-*GSA1*^*CG14*^ compared with those of NIL-*GSA1*^*WYJ*^ ^37,40–42^. In summary, we conclude that *GSA1* is indispensable for phenylpropanoid homeostasis including the biosynthesis of flavonoid glycosides and lignin.

### GSA1 exhibits wide-spectrum glucosyltransferase activity

Previous studies have indicated that the amino acids in the N-terminal region of UGTs contribute to the regio-specificity of sugar acceptors and that the PSPG box in the C-terminal region of UGTs plays a key role in sugar-donor binding^43,44^. The metabolomics data described above showed that levels of kaempferol, naringenin, quercetin and monolignols were higher in NIL-*GSA1*^*CG14*^ than in NIL-*GSA1*^*WYJ*^, whereas the levels of naringenin-7-*O*-glucoside, quercetin-7-*O*-glucoside and lignin were significantly lower. This inspired us to investigate whether GSA1 catalyzes glucosylation of these flavonoids and monolignols, and whether the substitutions A349T within the PSPG box and A246V result in a decrease in glucosyltransferase activity.

**Fig. 4:**
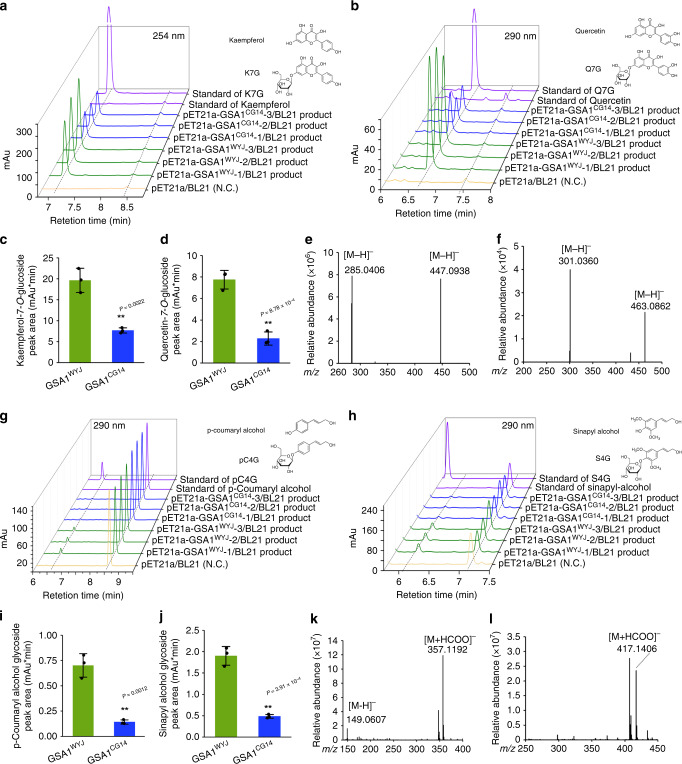
*GSA1* encodes a UDP-glucosyltransferase with activity toward flavonoids and monolignols. The in vitro glycosyltransferase activity of GSA1^WYJ^ and GSA1^CG14^ toward flavonoids (**a**, kaempferol. **b**, quercetin) determined by HPLC analyses. N.C., negative control. K7G, kaempferol-7-*O*-glucoside. Q7G, quercetin-7-*O*-glucoside. Comparison of the kaempferol-7-*O*-glucoside (**c**) and quercetin-7-*O*-glucoside (**d**) peak areas for product of GSA1^WYJ^ and GSA1^CG14^. The values represent the mean ± s.d. (*n* = 3 biological replicates). **P < 0.01 indicates a significant difference compared with GSA1^WYJ^ in a two-tailed Student’s *t* test. **e**, **f**, MS analyses of the glucoside products produced by GSA1^WYJ^. Mass spectra of kaempferol consisted of a parent molecule ion with *m/z* 285.0406 [M-H]^-^ and kaempferol-7-*O*-glucoside with *m/z* 447.0938 [M-H]^-^ (**e**). Mass spectra of quercetin consisted of a parent molecule ion with *m/z* 301.0360 [M-H]^-^ and quercetin-7-*O*-glucoside with *m/z* 463.0862 [M-H]^-^ (**f**). **g**, **h**, The in vitro glycosyltransferase activity of GSA1^WYJ^ and GSA1^CG14^ toward monolignols (**g**, p-coumaryl alcohol. **h**, sinapyl alcohol) determined by HPLC analyses. N.C., negative control. pC4G, p-coumaryl alcohol 4-*O*-glucoside. S4G, sinapyl alcohol 4-*O*-glucoside. **i**, **j**, Comparison of the p-coumaryl alcohol glycoside (**i**) and sinapyl alcohol glycoside (**j**) peak areas for product of GSA1^WYJ^ and GSA1^CG14^. The values represent the mean ± s.d. (*n* = 3 biological replicates). **P < 0.01 indicates significant differences compared with GSA1^WYJ^ in a two-tailed Student’s *t* tests. **k**, **l**, MS analyses of the glucoside products produced by GSA1^WYJ^. Mass spectra of p-coumaryl alcohol consisted of a parent molecule ion [M-H]^-^ with (*m/z*) 149.0607 and p-coumaryl alcohol glycoside methanoic acid adduct with *m/z* 357.1192 [M + HCOO]^-^ (**k**). Mass spectra of sinapyl alcohol methanoic acid adduct with *m/z* 417.1406 [M + HCOO]^-^ (**l**). The source data underlying Fig. 4c, d, i, and j are provided as Source Data file.

We thus heterologously expressed *GSA1* in *Escherichia coli* to explore the ability of the recombinant proteins to glucosylate flavonoids and monolignols. High-performance liquid chromatography (HPLC) analysis of enzymatic reaction products showed that kaempferol (Fig. 4a), quercetin (Fig. 4b), and naringenin (Supplementary Fig. 10a) were glucosylated by GSA1, producing potential kaempferol-7-*O*-glucoside (K7G), quercetin-7-*O*-glucoside (Q7G) and naringenin-7-*O*-glycoside (N7G) with exactly the same retention times as the authentic standards of K7G, Q7G and N7G, respectively. Moreover, the peak areas of K7G, Q7G, and N7G produced in GSA1^CG14^-mediated reactions were distinctly smaller than those produced by GSA1^WYJ^ (Fig. 4c, d and Supplementary Fig. 10b), further supporting the hypothesis that GSA1^CG14^ is a weakly functioning flavonoid glucosyltransferase. To further validate the identity of products of GSA1-mediated reactions, the putative K7G, Q7G and N7G products were analyzed by liquid chromatography-mass spectrometry (LC-MS). The GSA1^WYJ^ products displayed dominant ion peaks at *m/z* 447.0938 [M-H]^-^, 463.0862 [M-H]^-^, and 433.1144 [M-H]^-^ (Fig. 4e, f and Supplementary Fig. 10c), which correspond well to the peaks of the authentic standards K7G, Q7G, and N7G, respectively, implying that GSA1 is required for the 7-*O*-glucosylation of flavonoids.

To further investigate whether GSA1 uses monolignols as sugar acceptors, we performed HPLC analysis of the products of in vitro GSA1-mediated reactions toward monolignols. We found that GSA1 catalyzed the glucosylation of p-coumaryl alcohol (Fig. 4g), sinapyl alcohol (Fig. 4h), and coniferyl alcohol (Supplementary Fig. 10d), producing catalytic products with relatively longer retention times in HPLC analysis than those of the authentic standards of p-coumaryl alcohol 4-*O*-glucoside (pC4G), sinapyl alcohol 4-*O*-glucoside (S4G), and coniferin, respectively. These results implied that glucosylation of monolignols catalyzed by GSA1 does not occur at the 4-OH position. In addition, the peak areas of the potential p-coumaryl alcohol glycoside, sinapyl alcohol glycoside, and coniferyl alcohol glycoside products produced by GSA1^WYJ^ were significantly larger than those produced by GSA1^CG14^ (Fig. 4i, j and Supplementary Fig. 10e), indicating that the substitutions of A349T within PSPG box and A246V also result in the weak glucosyltransferase activity of GSA1^CG14^ toward monolignols. To further confirm the glucosylation of monolignols, the catalytic products produced by GSA1^WYJ^ were analyzed by LC-MS. We found that the products of GSA1^WYJ^ catalysis showed dominant ion peaks corresponding to a methanoic acid adduct at *m/z* 357.192 [M + HCOO]^-^, 417.1406 [M + HCOO]^-^ and 387.1298 [M + HCOO]^-^ (Fig. 4k, l and Supplementary Fig. 10f), which correspond well to the predicted p-coumaryl alcohol glycoside, sinapyl alcohol glycoside and coniferyl alcohol glycoside, respectively. In summary, we conclude that GSA1 catalyzes the glucosylation of monolignols, but does not produce 4-*O*-glucosides. The potential glycosylation sites might be alcoholic hydroxyls on the side chain.

To identify the key amino acids underlying the differential activity of GSA1^WYJ^ and GSA1^CG14^, we heterologously expressed mutated versions GSA1^A246V^ and GSA1^A349T^ in *E. coli*. HPLC analysis revealed that the glucosyltransferase activity of GSA1^A246V^ toward kaempferol and sinapyl alcohol was comparable to that of GSA1^WYJ^ and the glucosyltransferase activity of GSA1^A349T^ was comparable to that of GSA1^CG14^ (Supplementary Fig. 11a–d). These results imply that the alanine at position 349 within the conserved PSPG box domain is vital for the glucosyltransferase activity of GSA1. We also selected kaempferol as sugar acceptor to perform kinetic analysis of GSA1. The *Km* value of GSA1^WYJ^ toward kaempferol (25.54 ± 5.43 μM) showed no distinct difference from that of GSA1^CG14^ (42.78 ± 23.26 μM, Supplementary Fig. 11e), while a much lower *Km* value toward UDP-glucose was observed in GSA1^WYJ^ (51.06 ± 7.814 μM) than in GSA1^CG14^ (134.6 ± 21.63 μM, Supplementary Fig. 11f). These data imply that GSA1^WYJ^ has higher affinity toward UDP-glucose and the natural variations in GSA1^CG14^ might result in impaired UDP-glucose binding, which possibly leads to the decreased glucosyltransferase activity of GSA1^CG14^.

Collectively, these results demonstrate that GSA1 exhibits wide-spectrum glucosyltransferase activity toward both flavonoids and monolignols in vitro and that amino acid residue A349 within the PSPG box functions as a key amino acid that influences glucosyltransferase activity.

### GSA1 regulates metabolic flux redirection under abiotic stress

**Fig. 5:**
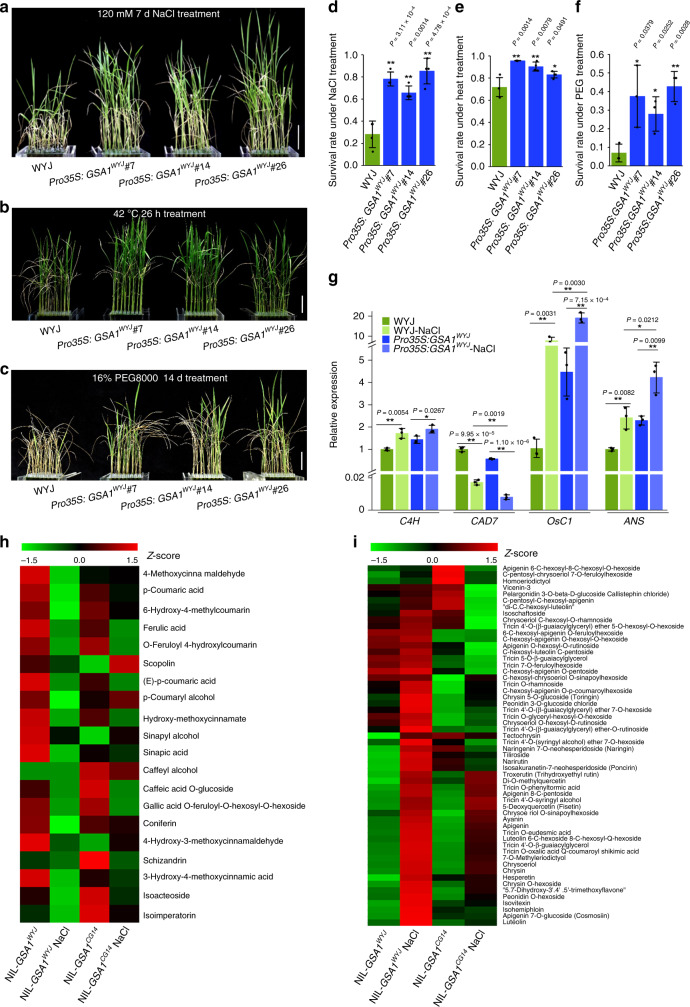
*GSA1* regulates abiotic stress tolerance associated with the redirection of metabolic flux. Fourteen-day-old seedlings of WYJ and overexpression lines were transferred to 120 mM NaCl for 7 days (**a**), 42 °C for 26 h (**b**) or 16% PEG8000 for 14 days (**c**) and recovered for 14 days. Scale bar = 5 cm. Relative survival rates of 14-day-old seedlings of WYJ and overexpression lines after NaCl treatment (**d**, *n* = 4 biological replicates), heat treatment (**e**, *n* = 4 biological replicates) and PEG treatment (**f**, *n* = 3 biological replicates). **d**–**f** 24 plants per biological replicate. **g** The relative expression levels of *OsC4H*, *OsCAD7*, *OsC1* and *OsANS* determined by qRT-PCR before and after NaCl treatment in WYJ and *Pro35S:GSA1*^*WYJ*^ seedlings (*n* = 3 biological replicates). The ubiquitin gene was used for normalization. The values in **d**–**g** represent the mean ± s.d. *P < 0.05 and **P < 0.01 indicate significant differences compared with WYJ or NIL-*GSA1*^*WYJ*^ in two-tailed Student’s *t* tests. **h**, **i** Clustering heat maps of the relative levels of phenylpropanoid (**h**) and flavonoids (**i**) after NaCl treatment in NIL-*GSA1*^*WYJ*^ and NIL-*GSA1*^*CG14*^ seedlings (*n* = 3 biological replicates) determined from widely targeted metabolomics data. Standard-scores (Z-scores) were used as the numerical signs to evaluate the standard deviations from the mean of the corresponding samples. The source data underlying Fig. 5d–i is provided as Source Data file.

Flavonoid glycosides including anthocyanins accumulate under abiotic stresses such as drought, salinity and high-temperature, protecting plants against oxidative damage^21–25^. Given that *GSA1* plays a significant role in modulating flavonoid glycoside profiles, we asked whether *GSA1* is necessary for abiotic stress tolerance. Compared with WYJ seedlings, seedlings of the overexpression lines *Pro35S:GSA1*^*WYJ*^ exhibited improved salt tolerance, thermotolerance and drought tolerance (Fig. 5a–c and Supplementary Fig. 12a–c), and increased survival rate under abiotic stress (Fig. 5d–f). However, NIL-*GSA1*^*CG14*^ seedlings exhibited much higher sensitivity to abiotic stress compared with NIL-*GSA1*^*WYJ*^ seedlings (Supplementary Fig. 13a–f). Similarly, seedlings of the overexpression lines *Pro35S:GSA1*^*CG14*^ and the *GSA1* knock-out lines *KO-GSA1* exhibited decreased salt tolerance compared with WYJ seedlings (Supplementary Fig. 13g–j**)**. The complementary transgenic line showed NIL-*GSA1*^*WYJ*^ phenotypes with respect to survival rate under NaCl treatment (Supplementary Fig. 13k, l**)**. In addition, the expression level of *GSA1* in NIL-*GSA1*^*WYJ*^ and NIL-*GSA1*^*CG14*^ was higher after NaCl treatment, and the increase in NIL-*GSA1*^*WYJ*^ was larger than that in NIL-*GSA1*^*CG14*^, implying that *GSA1* was induced by abiotic stress (Supplementary Fig. 13m). Taken together, these results strongly suggest that *GSA1* functions directly in abiotic stress tolerance.

In addition, the knock-out lines of the two other UGT genes mapping to the *GSA1* target region, *LOC_Os03g55030* and *LOC_Os03g55050*, generated using the CRISPR/Cas9 system exhibited no significant difference compared with WYJ with respect to survival rate under NaCl treatment (Supplementary Fig. 14a–f), implying that the other UGT genes within the *GSA1* target region have no effect on abiotic stress tolerance.

To further investigate the role of *GSA1* in metabolic processes regulating abiotic stress tolerance, we examined the expression levels of genes involved in the phenylpropanoid pathway under salt stress and normal conditions. Previous studies showed that the phenylpropanoid pathway is induced under abiotic stress and that cinnamate 4-hydroxylase (C4H) catalyzes the second step in the central phenylpropanoid pathway, providing precursors for synthesis of all of the downstream metabolites^23,37,38^. Interestingly, *OsC4H* was significantly up-regulated under NaCl treatment in both WYJ and *Pro35S:GSA1*^*WYJ*^ seedlings compared with normal conditions, as well as in NIL-*GSA1*^*WYJ*^ and NIL-*GSA1*^*CG14*^ (Fig. 5g and Supplementary Fig. 15a), indicating that the central phenylpropanoid pathway is induced under salt stress. However, the increase in *OsC4H* expression level in NIL-*GSA1*^*CG14*^ was obviously smaller than that in NIL-*GSA1*^*WYJ*^ (Supplementary Fig. 15a), and there was no difference in the *OsC4H* expression level in *KO-GSA1* between normal conditions and NaCl treatment (Supplementary Fig. 15b), suggesting that *GSA1* is essential for induction of the central phenylpropanoid pathway in response to abiotic stress. To further investigate the metabolic flux of induced central phenylpropanoid pathway flow into which branch, expression levels of genes responsible for the biosynthesis of lignin and flavonoids were examined. Cinnamyl alcohol dehydrogenase (CAD) is required for monolignol biosynthesis and is involved in the lignin pathway^37,45^. The expression level of *OsCAD7* was remarkably decreased under salt stress in WYJ and *Pro35S:GSA1*^*WYJ*^ seedlings, as well as in NIL-*GSA1*^*WYJ*^, while there was no obvious difference in *OsCAD7* expression between salt stress and normal conditions in *KO-GSA1* and NIL-*GSA1*^*CG14*^ seedlings (Fig. 5g and Supplementary Fig. 15a,b). The complementary transgenic line showed no significant difference in the expression levels of these genes compared with NIL-*GSA1*^*WYJ*^ (Supplementary Fig. 15c). Taken together, these results demonstrate that *GSA1* takes part in the down-regulation of the lignin pathway under abiotic stress.

However, the expression levels of *OsC1*, which encodes an R2R3-MYB transcriptional factor regulating the biosynthesis of anthocyanin in leaf and spikelet hulls^41,42,46^, and *OsANS*, which encodes anthocyanidin synthase^40^, were clearly more highly expressed under salt stress in both WYJ and *Pro35S:GSA1*^*WYJ*^ than under normal conditions, as well as in NIL-*GSA1*^*WYJ*^ and NIL-*GSA1*^*CG14*^ seedlings (Fig. 5g and Supplementary Fig. 15a), indicating that the biosynthesis of flavonoids including anthocyanins is greatly up-regulated by abiotic stress. Similar to *OsC4H*, the increase in *OsC1* and *OsANS* expression levels in *Pro35S:GSA1*^*WYJ*^ was much larger than that in WYJ; the increase in NIL-*GSA1*^*WYJ*^ was much larger than that in NIL-*GSA1*^*CG14*^; and the increase in WYJ was much larger than that in *KO-GSA1* (Fig. 5g and Supplementary Fig. 15a, b). The complementary transgenic line showed no significant difference in the expression levels of these genes compared with NIL-*GSA1*^*WYJ*^ (Supplementary Fig. 15c). These data reveal that *GSA1* is of high importance for up-regulation of flavonoids biosynthesis including anthocyanins under abiotic stress. In summary, *GSA1* is required for the activation of the phenylpropanoid pathway and flavonoid biosynthesis, as well as down-regulation of the lignin pathway, under abiotic stress.

To investigate whether the redirection of metabolic flux is responsible for abiotic stress tolerance, we performed a widely targeted metabolomics assay of NIL-*GSA1*^*WYJ*^ and NIL-*GSA1*^*CG14*^ seedlings under normal and salt stress conditions. In agreement with the expression levels of genes related to the central phenylpropanoid pathway and lignin pathway, the relative levels of some phenylpropanoid metabolites (i.e., caffeate, zingerone, 4-hydroxycoumarin) were clearly increased under salt stress in NIL-*GSA1*^*WYJ*^ and NIL-*GSA1*^*CG14*^ seedlings, whereas the relative levels of other phenylpropanoid metabolites, especially monolignols (p-coumaryl alcohol, sinapyl alcohol, and coniferyl alcohol, which accept sugars from GSA1) were largely reduced (Supplementary Fig. 16a). Intriguingly, among these down-regulated phenylpropanoid metabolites, the degree of decrease was obviously smaller in NIL-*GSA1*^*CG14*^ seedlings compared with NIL-*GSA1*^*WYJ*^ seedlings, and some phenylpropanoid metabolites were even up-regulated in NIL-*GSA1*^*CG14*^, including p-coumaryl alcohol and sinapyl alcohol (Fig. 5h and Supplementary Fig. 16a). In addition, the down-regulation of the phenylpropanoid metabolites including the lignin pathway metabolites was much more obvious in *Pro35S:GSA1*^*WYJ*^ than in WYJ under salt stress (Supplementary Fig. 17a). These results suggest that *GSA1* plays a key role in the flowing out of metabolic flux from lignin pathway under salt stress. On the contrary, the relative levels of large numbers of glycosidic flavonoids were distinctly increased under salt stress in NIL-*GSA1*^*WYJ*^ seedlings, whereas the levels of these flavonoids in NIL-*GSA1*^*CG14*^ seedlings did not increase as much as in *GSA1*^*WYJ*^ seedlings (Fig. 5i), implying that the metabolic flux redirection is disrupted in NIL-*GSA1*^*CG14*^ seedlings. The widely targeted metabolomics data showed that apigenin glucoside derivatives, particularly apigenin-7-*O*-glucoside, chrysoeriol glucoside derivatives and anthocyanins, which are required for abiotic stress, accumulated at higher levels in NIL-*GSA1*^*WYJ*^ seedlings than in NIL-*GSA1*^*CG14*^ seedlings under salt stress (Fig. 5i and Supplementary Fig. 16b). In addition, glycosidic flavonoids including apigenin-7-*O*-glucoside, chrysoeriol glucoside derivatives, and anthocyanins accumulated at higher levels in *Pro35S:GSA1*^*WYJ*^ seedlings than in WYJ seedlings under salt stress (Supplementary Fig. 17b**)**, which possibly results in the enhanced abiotic stress tolerance in *Pro35S:GSA1*^*WYJ*^ seedlings. Taken together, we conclude that *GSA1* plays a key role in the induction of central phenylpropanoid pathway and flavonoid biosynthesis pathway, as well as in the redirection of metabolic flux from the lignin pathway to the flavonoid biosynthesis pathway in response to abiotic stress.

## Discussion

Grain size determines grain weight, which is an essential agronomic trait affecting rice yield. A number of major QTLs regulating grain size have been identified and found to be involved in the ubiquitin-proteasome pathway, G-protein signaling, mitogen-activated protein kinase signaling, phytohormones signaling, and regulation of transcriptional factors^1^. Flavonoids accumulate in response to abiotic stress and protect plants by scavenging ROS and reducing oxidative damage^21–24,47^. However, our knowledge of the mechanism underlying the synergistic regulation of grain yield and abiotic stress tolerance remains elusive. In this study, we identified a QTL, *GSA1*, which functions in the glucosylation of monolignols and flavonoids. *GSA1* regulates grain size and abiotic stress tolerance associated with the lignin pathway and flavonoid glycoside profiles.

Previous studies have revealed that auxin is of vital importance in the regulation of grain size. Enhanced expression of *Big Grain 1* (*BG1*), which is a positive regulator of auxin response and transport results in extra-large grains^48^. Moreover, plants with mutation in *TILLERING AND SMALL GRAIN 1* (*TSG1*), which controls local auxin biosynthesis and affect auxin signaling and transport, exhibit small grains as a result of disruption of localized cell proliferation and cell expansion^36^. Consistent with these previous studies linking auxin to grain size, we found that *GSA1* regulates grain size by finely modulating cell proliferation and cell expansion during different stages of spikelet development (Fig. 2b–f and Supplementary Fig. 9c–j). Intriguingly, the endogenous auxin level and relative expression levels of auxin-related genes, especially *BG1*, *TSG1* and its homologues *TARL*, *TRAL1,* and *TARL2*, were notably reduced in NIL-*GSA1*^*CG14*^ (Fig. 2g–i), revealing that *GSA1* modulates grain size by indirectly affecting the auxin levels, PIN1 protein levels, and auxin-related gene expression.

The synthetic compound 1-N-naphthylphthalamic acid (NPA) has been used as an auxin transport inhibitor, and aglycone flavonoids that share structural similarity with NPA also function as endogenous inhibitors of auxin transport^49^. In *Arabidopsis*, flavonoid biosynthesis pathway is blocked in *transparent testa* (*tt*) mutants. The *tt3* mutant, which contains no functional dihydroflavanol-4-reductase, accumulates excess kaempferol and quercetin. The *tt7* mutant, which lacks flavonoid-3‘-hydroxylase (F3’H), accumulates excess kaempferol. Both *tt3* and *tt7* mutants exhibit reduced auxin transport and notably decreased seed size^50,51^. Consistent with these studies, we found that levels of the aglycone flavonoids kaempferol, quercetin, and naringenin were significantly higher and the expression levels of flavonoid biosynthesis genes were obviously down-regulated in the young panicles of NIL-*GSA1*^*CG14*^ (Fig. 3b, d), which formed smaller rice grains than NIL-*GSA1*^*WYJ*^ (Fig. 1a–d). Flavonoids inhibit auxin transport by affecting the activity of serine–threonine PINOID proteins which regulate the subcellular localization of PIN proteins^52,53^. Notably, we found that the *PIN* genes were significantly down-regulated in NIL-*GSA1*^*CG14*^, in which high levels of aglycone flavonoids accumulated (Figs. 2h, i, 3b). These results suggest that flavonoids play critical roles in controlling auxin transport and regulating grain size. In addition, we found that NIL-*GSA1*^*CG14*^, in which GSA1 has reduced glucosyltransferase activity, had less flavonoid 7-*O*-glycosylation and decreased expression levels of genes related to the central phenylpropanoid pathway and flavonoid biosynthesis pathway (Figs. 3b, d, 4a–f). We hypothesize that flavonoid glycosylation catalyzed by GSA1 plays a crucial role in feedback regulation of the central phenylpropanoid pathway and flavonoid biosynthesis pathway, which contribute to auxin transport and the regulation of grain size.

Previous studies showed that the lignin pathway is associated with seed size. In *Arabidopsis*, overexpression of *Tap46*, which encodes a regulatory subunit of protein phosphatase 2 A, enhances cell size and cell number, resulting in larger seed size, and also induces genes involved in lignin biosynthesis^54^. In contrast, mutation of *NARROW AND ROLLED LEAF 2* (*NRL2*) in rice causes a reduction in lignin content and altered phenylpropanoid metabolism, resulting in defective cell differentiation and decreased grain width and grain thickness^55^. Similarly, the down-regulation of lignin pathway gene expression and decrease in lignin content in NIL-*GSA1*^*CG14*^ were correlated with interfered cell proliferation and cell expansion, leading to the production of smaller grains (Figs. 2b–f, 3c, d). These results further clarify that the lignin pathway is involved in cell proliferation and cell expansion and regulates grain size.

The central phenylpropanoid pathway, in which the first three steps are catalyzed by phenylalanine ammonia-lyase (PAL), C4H, and 4-coumarate: CoA ligase, provides precursors for the downstream metabolic pathway^37,38^. The lignin and flavonoid biosynthesis pathways are two major branches of the phenylpropanoid pathway and share the central phenylpropanoid pathway. NIL-*GSA1*^*CG14*^, in which GSA1 has decreased glucosyltransferase activity, had a lower content of lignin and flavonoid glycosides compared with NIL-*GSA1*^*WYJ*^, and the metabolic flux from the lignin pathway to the flavonoid biosynthesis pathway under abiotic stress was disrupted (Figs. 3a, b, 5g–i). This finding indicates that GSA1 functions directly in coordinating metabolic flux among diverse phenylpropanoid-dependent branches. Flavonoids including anthocyanins have been extensively studied for their roles in protecting plant against abiotic stress^21,22,24^. A recent study revealed that flavonoid glycosides such as apigenin glucoside derivatives, particularly apigenin-7-*O*-glucoside and chrysoeriol glucoside derivatives accumulate under abiotic stress and act as ROS scavengers to reduce oxidative damage in rice^47^. Consistent with this, the relative levels of these flavonoid glycosides and anthocyanins were clearly reduced in NIL-*GSA1*^*CG14*^, which is more sensitive to abiotic stress than NIL-*GSA1*^*WYJ*^ (Fig. 5i and Supplementary Fig. 16b), suggesting that the accumulation of flavonoid glycosides and anthocyanins under abiotic stress is regulated by *GSA1*. In summary, we uncovered a strategy utilized by plant to respond to abiotic stress: enhancing the accumulation of flavonoid glycosides and anthocyanins through *GSA1*-controlled metabolic flux redirection.

**Fig. 6:**
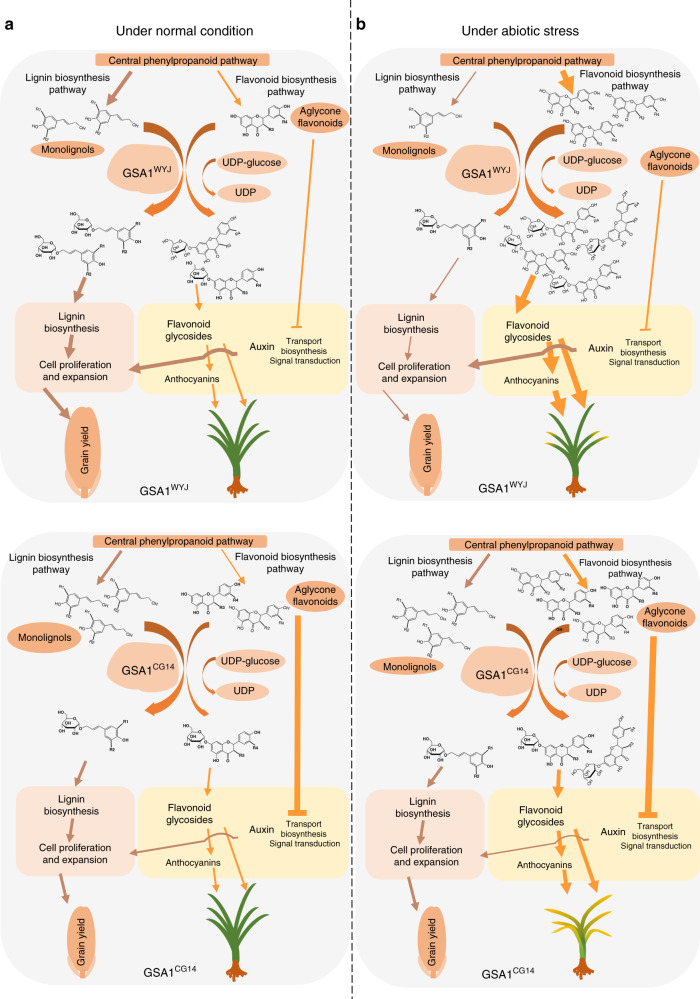
Proposed working model of the role of GSA1 in the regulation grain size and abiotic stress in rice. **a** Under normal conditions, GSA1^WYJ^ catalyzes the glycosylation of monolignol and contributes to lignin biosynthesis, which is required for cell proliferation and cell expansion. In addition, GSA1^WYJ^ catalyzes the glycosylation of flavonoids, which is indispensable for plant development. While the natural variations in GSA1^CG14^ lead to decreased glucosyltransferase activity and cause the accumulation of monolignols and aglycone flavonoids. The subsequent disruption of lignin biosynthesis, auxin transport, and signal transduction result in the suppression of cell proliferation and cell expansion in NIL-*GSA1*^*CG14*^ spikelets, ultimately reducing grain size and grain yield. **b** Under abiotic stress (salt, drought or heat stress), metabolic flux in GSA1^WYJ^ redirects to the biosynthesis of anthocyanins and flavonoid glycosides to enhance tolerance to abiotic stress. In contrast, the decreased glucosyltransferase activity of GSA1^CG14^ results in a lack of anthocyanins and flavonoid glycosides, in addition to the accumulation of aglycone flavonoids. GSA1^CG14^ thereby leads to defects in metabolic flux redirection and a reduction in tolerance to abiotic stress.

Plants grow in continuously changing environments and have evolved sophisticated mechanisms to coordinate the flow of resources between growth and abiotic stress tolerance. However, how plant growth and abiotic stress tolerance are synergistically regulated remains largely unknown. Here, we propose a working model for the regulation of grain size and abiotic stress tolerance associated with metabolic flux redirection by GSA1. Under normal conditions, monolignols are glucosylated by GSA1^WYJ^ and contribute to lignin biosynthesis, which is required for plant growth and development. Flavonoids glucosylated by GSA1^WYJ^ also take part in the plant response to changing environments. In contrast, the decreased glucosyltransferase activity of GSA1^CG14^ results in the accumulation of monolignols and aglycone flavonoids. The subsequent repression of lignin biosynthesis, inhibition of auxin transport, biosynthesis and the downstream signaling leading to the disruption of cell proliferation and cell expansion in NIL-*GSA1*^*CG14*^ spikelets, resulting in the production of smaller grains (Fig. 6a). Under abiotic stresses such as salt, drought, and heat, the phenylpropanoid pathway in NIL-*GSA1*^*WYJ*^ is induced and metabolic flux is redirected toward the accumulation of flavonoid glycosides and anthocyanins, which diminish the damage caused by abiotic stress. In contrast, the natural variations in GSA1^CG14^ lead to decreased glucosyltransferase activity and result in the accumulation of aglycone flavonoids, which down-regulate the central phenylpropanoid pathway and flavonoid biosynthesis pathway through feedback inhibition. The consequent disruption of metabolic flux redirection and lack of anthocyanin and flavonoid glycoside accumulation result in NIL-*GSA1*^*CG14*^ having reduced tolerance to abiotic stress (Fig. 6b). Hence, this discovery not only established a regulatory mechanism underlying grain size but also uncovered a strategy to enhance abiotic stress tolerance in rice. *GSA1* is a valuable gene resource that is expected to accelerate the discovery of strong alleles that coordinately confer high crop productivity and enhanced abiotic stress tolerance, providing strategies for molecular design breeding and sustainable food security.

## Methods

### Plant material and growth conditions

A set of CSSLs was constructed to clone and identify QTLs for grain size and abiotic stress, using African rice variety CG14 (*O. glaberrima*) as the donor parent and Asian rice variety Wuyunjing (WYJ, *O. sativa japonica*) as the recurrent parent^31^. A CSSL containing *GSA1* named SG48, was selected and backcrossed several times to WYJ. We selected plants in which the region around *GSA1* was heterozygous, and almost all other regions were homozygous for WYJ in order to develop the segregating BC_4_F_2_ populations for fine mapping of *GSA1* through marker-assisted selection. From the BC_5_F_2_ generation, we constructed a NIL of *GSA1*, NIL-*GSA1*^*CG14*^ with a very small CG14 chromosomal region containing the *GSA1* locus in the WYJ genetic background, and its isogenic control NIL-*GSA1*^*WYJ*^. All rice plants were cultivated in experimental fields in Shanghai (from May to October) and Hainan (from November to April of the following year) under natural growth conditions.

### Fine mapping of GSA1

To fine-map the *GSA1* locus, we used the traits grain width, grain length, and 1000-grain weight measured in 200 BC_4_F_2_ plants and seven newly designed molecular markers in a target region containing *GSA1*. Molecular markers D3-125.1 and D3-125.48, which flank *GSA1*, were used to detect recombinants in 5260 BC_4_F_2_ plants. *GSA1* was then mapped to a 29.47-kb region on the long arm of chromosome 3. The candidate *GSA1* genes from WYJ and CG14 genomic DNA were sequenced and compared. PCR primers are shown in Supplemental Table 3. Linkage maps were constructed using MAPMAKER/EXP 3.0 software^56^. Putative QTLs were identified using the MAPMAKER/QTL program^57^.

### RNA extraction and qRT-PCR

Total RNA was extracted from various rice tissues using a Plant RNA Kit (Omega). Reverse transcription and cDNA synthesis were performed using the ReverTra Ace qPCR RT Master Mix with gDNA Remover (Toyobo) from 500 ng of total RNA. The ABI 7300 Real Time PCR System and Fast Start Universal SYBR Green Master Mix with ROX (Roche) were used to perform quantitative RT–PCR analysis. 7300 system Software (Version 1.4.0) was used for data collection and the expression data were analyzed using the 2^-△△CT^ method and Microsoft Excel 2013. Ubiquitin or actin genes were used for normalization. There were three biological replicates for all analyses. PCR primer sets for gene amplification are shown in Supplementary Table 3.

### Histochemical analysis and scanning electron microscopy

Spikelet hulls harvested at the booting stage and mature stage were fixed in FAA (50% ethanol, 5% glacial acetic acid, and 5% formaldehyde) overnight at 4 °C, then dehydrated in an ethanol series. For histochemical analysis, after fixing with xylene, the spikelet hulls were embedded in paraplast (Sigma-Aldrich) and sliced into 8-mm thin sections with a rotary microtome (Leica). The prepared tissue sections were stained with safranin and then observed under a light microscope (Carl Zeiss). For scanning electron microscopy, the spikelet hulls were then dried in a critical point drier(Leica), gold sputter coated, and observed under a scanning electron microscope (Hitachi). Image J software was used to measure cell size and cell number. Leica Application Suite (Version V4.2) was used for collecting the images of young caryopses.

### Plasmid construction and plant transformation

To produce the pCAMBIA1306-*GSA1* overexpression constructs, the full-length coding sequences of *GSA1* were amplified from NIL-*GSA1*^*WYJ*^ and NIL-*GSA1*^*CG14*^ and cloned into the plant binary vector pCAMBIA1306 under the control of the CaMV 35 S promoter. The CRISPR/Cas9 system was used for gene editing constructs of *GSA1*^58^. *GSA1*-g*RNA1* and *GSA1*-*gRNA2* were cloned into the pYLgRNA-OsU3 and pYLgRNA-OsU6a vector, respectively. *GSA1-gRNA1* and *GSA1-gRNA2* sequence were transferred to the *pYLCRISPR/Cas9-MTmono* vector to generate *pYLCRISPR/Cas9-MTmono-OsU3-gRNA1-OsU6a-gRNA2* constructs. *Agrobacterium tumefaciens*-mediated transformation of rice was performed using strain EHA105^59^. Constructs were transferred into EHA105 agrobacterium cells. Agrobacterium cells were grown in LB medium at 28 °C overnight and then co-cultivated with rice callus for three days. The transgenic plants were screened out with 25 μg mL^−1^ hygromycin. NEBuilder HIFI DNA Assembly Master Mix (NEB) was used to generate plasmid constructs. All DNA constructs used in this study were confirmed by sequencing, and all negative controls and positive transgenic plants were identified by PCR amplification of hygromycin and sequencing of the *GSA1* target region. PCR primer sets are shown in Supplemental Table 3.

### RNA sequencing

RNA sequencing was performed by Hanyu Bio-Tech (Shanghai, China). Total RNA from all samples was isolated using Trizol (Invitrogen). The quality and integrity of RNA were examined by agarose gel electrophoresis and 2100 Bioanalyzer. The qualified RNA was treated with DNase (5 U μL^−1^) (TaKaRa) at 37 °C for 30 min. The DNase treated RNA was purified using Dynabeads Oligo (dT) 25 (Life). The library was prepared using the NEBNext® UltraTM RNA Library Prep Kit for Illumina following the manufacturer’s instructions. The quality of the cDNA library was evaluated by performing 2% agarose gel electrophoresis, qubit quantification, and high-sensitivity DNA chip analysis. The sequencing library (10 ng) was sequenced on the Illumina NOva platform to obtain 2 × 150 bp data.

### Widely targeted metabolomics assay

The widely targeted metabolomics assay was performed by Metware (Wuhan, China). The freeze-dried plant samples were crushed using a mixer mill (MM 400, Retsch) with a zirconia bead for 1.5 min at 30 Hz. Powder (100 mg) was weighed and extracted overnight at 4 °C with 1.0 mL 70% aqueous methanol. Following centrifugation at 10,000 × *g* for 10 min, the sample extracts were absorbed (CNWBOND Carbon-GCB SPE Cartridge, 250 mg, 3 mL; ANPEL, Shanghai, China) and filtrated (SCAA-104, 0.22μm pore size; ANPEL) before analysis. The extracts were analyzed using an LC-ESI-MS/MS system. The analytical conditions were as follows, HPLC: column, Waters ACQUITY UPLC HSS T3 C18 (1.8 µm, 2.1 mm*100 mm); solvent system, water (0.04% acetic acid): acetonitrile (0.04% acetic acid); gradient program, 95:5 V/V at 0 min, 5:95 V/V at 11.0 min, 5:95 V/V at 12.0 min, 95:5 V/V at 12.1 min, 95:5 V/V at 15.0 min; temperature, 40 °C; flow rate, 0.40 mL min^-1^; injection volume: 2 μL. We then connected the effluent to an ESI-triple quadrupole-linear ion trap (Q TRAP)-MS. Triple quadrupole (QQQ) scans and LIT were acquired on a Q TRAP mass spectrometer, API 6500 Q TRAP LC/MS/MS System. This system was equipped with ESI Turbo Ion-Spray interface and operating in a positive ion mode. Analyst 1.6.3 software (AB Sciex) controlled this system. Parameters of the ESI source operation were set as follows: the collision gas was set at high; ion source, turbo spray; ion spray voltage 5500 V; source temperature, 500 °C; ion source gas I, gas II and curtain gas were set at 55, 60, and 25.0 psi, respectively. We acquired QQQ scans as multiple reaction monitoring (MRM) experiments with nitrogen as collision gas. The collision gas was set to 5 psi. We performed collision energy (CE) and declustering potential (DP) for individual MRM transitions with optimization of CE and DP. According to the metabolites eluted within each period, a specific set of MRM transitions were monitored for this period.

### Endogenous auxin level measurement

Determination of endogenous auxin concentration was performed by Metware (Wuhan, China). Diverse fresh rice tissues were collected, weighed, immediately frozen in liquid nitrogen immediately, and stored at −70 °C. Plant samples (120 mg fresh weight) were frozen in liquid nitrogen, ground into powder, and then extracted with methanol/water (8/2) at 4 °C. The extract was centrifuged at 12,000 × *g* at 4 °C for 15 min. The supernatant was then collected and evaporated to dryness under a nitrogen gas stream, then reconstituted in methanol/water (3/7). The sample was centrifuged, and the resulting supernatant was analyzed using an LC-ESI-MS/MS system.

### Protein expression in Escherichia coli and enzyme activity assay

The full-length cDNA sequence of G*SA1* was cloned into the pET21a (Novagen) vector between the *Nde*I and *Not*I (NEB) sites. The constructs were then introduced into *E. coli* strain BL21 (DE3) pLysS, and the transformants were grown in LB medium containing 100 mg mL^−1^ ampicillin at 37 °C until OD_600_ reached 0.6. Expression of the GSA1 protein was induced by addition of 0.3 mM isopropyl β-D-1-thiogalactopyranoside and incubation at 16 °C for 20 h. The cells were harvested by centrifugation (4000 × *g*, 5 min) at 4 °C and resuspended in 1 mL of extraction buffer (100 mM NaCl, 20 mM Tris-HCl, pH 8.0). Two microliters of MaCl_2_ (1 M) and 10 μL of phenylmethylsulfonyl fluoride (PMSF, 100 mM) were added into the suspension and the sample was kept on ice for 30 min. The suspension was then lysed by using a sonication homogenizer (80 W, five cycles) and centrifuged (11,000 × *g*, 45 min) at 4 °C to obtain the supernatant, which served as crude enzyme extract. The enzyme activity assay was performed in a 200 μL aliquot of reaction mixture containing 10 mM MgCl_2_, 1.5 mM UDP-glucose as the sugar donor, 250 μM monolignol or flavonoid as the sugar acceptor, 200 mM glycine-NaOH buffer (pH 8.6) and 50 μL crude enzyme extract. The reaction mixtures were incubated at 37 °C for 2 h, and 200 μL methanol was added to quench the reaction. The enzymatic reaction mixtures were centrifuged (21,000 × *g*, 20 min) at 4 °C. The supernatant was then subjected to HPLC/UPLC-ESI-MS analysis.

### Lignin content measurement

The isolation of cell walls was described by Turner and Somerville^60^. Diverse rice tissues of three biological replications were harvested, frozen in liquid nitrogen and ground to fine powder using the Tissuelyser (Jingxin, Shanghai). Soluble material was extracted three times with 70% ethanol for 1 h each time at 70 °C to obtain the crude cell wall fraction. The samples were dried at 50 °C to a constant weight. Measurement of lignin content was done as described by Musel^61^. Powdered cell wall materials (15 mg) were hydrolyzed with 0.3 mL thioglycolic acid and 1.5 mL 2 mol L^−1^ HCl in a 2.5 mL tube for 4 h at 95 °C. The materials were cooled down to room temperature and then centrifuged (15,000 × *g*, 15 min). The pellets were washed with distilled water three times and treated with 1.5 mL 0.5 mol L^−1^ NaOH for 16 h at 20 °C with shaking to extract the lignothioglycolic acid. The samples were centrifuged (15,000 × *g*, 15 min) to obtain the supernatant. NaOH (0.4 mL) was added to re-extract pellet in order to obtain a second supernatant, which was combined with the first supernatant and acidified with 0.4 mL concentrated HCl. The samples were mixed and incubated for 4 h at 4 °C and then centrifuged (15,000 × *g*, 20 min). The pellet was dissolved in 1 mL 0.5 mol L^−1^ NaOH. The absorbance of the samples was measured at 280 nm, and NaOH was used as a blank. The relative content of lignin was determined by OD_280_ g^−1^ of the cell wall material.

### Laboratory growth conditions and abiotic stress treatments

The rice plants were grown under a 10 h dark/14 h light cycle at 50% humidity and 26 °C in Yoshida’s rice seedling culture solution. For NaCl treatment, 120 mM NaCl was dissolved in Yoshida’s rice seedling culture solution, and 14-day-old seedlings were treated for 7 days, and then recovered under normal conditions for 14 days. For polyethylene glycol (PEG) treatment, 14-day-old seedlings were treated with Yoshida’s rice seedling culture solution containing 16% PEG8000 for 2 or 3 weeks and recovered under normal conditions for 14 days. For heat treatment, 14-day-old seedlings were treated with Yoshida’s rice seedling culture solution at 42 °C for dozens of hours and recovered under normal conditions for 14 days.

### Plant protein extraction and western blot analysis

We harvested the ~10 cm young panicles from NIL-*GSA1*^*WYJ*^ and NIL-*GSA1*^*CG14*^. Plant total protein extraction kit (Sigma) was used to extract the soluble proteins of young panicles. Fine powder of the young panicles was acquired by grinding the panicles in liquid nitrogen using pestles and mortars. Methanol and acetone were used in sequence to rinse the powder. We dried the powder after removing the supernatant from the final extraction using acetone. The samples were dissolved using reagent type 4 working solution and were denatured by the adding the concentrated sodium dodecyl sulfate loading buffer. After boiling for 5 min, the samples were separated by 10% sodium dodecyl sulfate-polyacrylamide gel electrophoresis. The endogenous protein levels of OsPIN1 (*LOC_Os02g50960*) in NIL-*GSA1*^*WYJ*^ and NIL-*GSA1*^*CG14*^ were visualized by immunoblot analysis using the rabbit polyclonal anti-PIN1 (Abiocode, R2114-3, 1:2000 dilution). The loading control was blotted using the anti-actin antibody (Abmart, M20009, 1:2000 dilution).

### Nucleotide diversity analysis

To investigate the nucleotide diversity of *GSA1*, the SNPs of a ~3.3-kb genomic region containing the ~1-kb promoter and the ~1.6-kb entire ORF of *GSA1* were downloaded for all accessions. The DNA polymorphic data were downloaded from Rice3K data for *japonica* and *indica* accessions^33^, from the Rice Haplotype Map Project data for *O. rufipogon* accessions^34^, and from Wang *et al*. for *O. glaberrima* and *O. barthii*^35^. The nucleotide diversity was calculated for *GSA1* region with 500-bp window and 50 bp step by using vcftools^62^.

### Natural variation analysis of GSA1

To identify natural variation in the PSPG box of GSA1, we performed acid sequence alignment of GSA1 and its homologs using MEGA 6.0. Rice Gene annotation referred to RGAP (http://rice.plantbiology.msu.edu/). GSA1 homologous peptide sequences from monocots and dicots referred to Phytozome (https://phytozome.jgi.doe.gov/pz/portal.html). The Neighbor-Joining Tree was drawn by MEGA 6.0 and modified by iTol^63^.

### Reporting Summary

Further information on research design is available in the Nature Research Reporting Summary linked to this article.

## Supplementary information

Supplementary Information

Peer Review File

Reporting Summary

## Data Availability

Data supporting the findings of this work are available within the paper and its Supplementary Information files. A reporting summary for this article is available as a Supplementary Information file. The genetic materials generated and analyzed during the current study are available from the corresponding author upon request. The source data underlying Figs. 1b–f, h–j, 2a, c–i, 3, 4c, d, i, j, 5d–i, as well as Supplementary Figs. 1a–d, g–j, 2b–e, 3, 6, 7a–b, d–f, h–j, 7l–n, 8c–e, h–k, 9a, e–j, l, n–r, 10b, e, 11b, d–f, 13b, d, f, h, j, l, m, 14c, f, 15, 16, and 17 are provided as a Source Data file.

## Source data

Source Data
