# Supplementary material for: UDP-glucosyltransferase regulates grain size and abiotic stress tolerance associated with metabolic flux redirection in rice
Source: Nat Commun. 2020 May 26;11:2629. doi: 10.1038/s41467-020-16403-5 (PMC7250897; doi:10.1038/s41467-020-16403-5)
Supplement: Supplementary file 1 — Supplementary Information [file 41467_2020_16403_MOESM1_ESM.pdf]

UDP-glucosyltransferase regulates grain size and abiotic stress tolerance associated with metabolic flux redirection in rice

Dong *et al.*

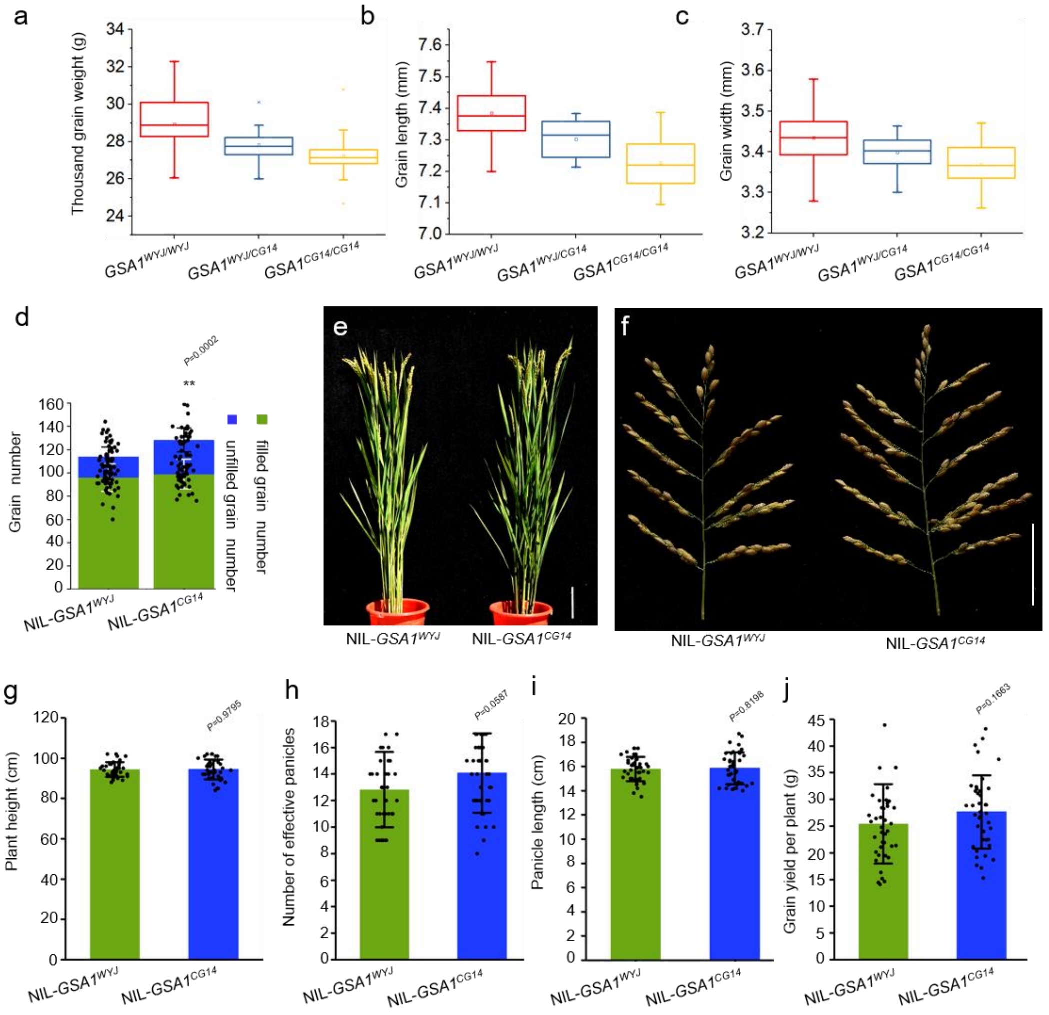

Supplementary Figure 1. Phenotype of NIL-*GSA1*^{WYJ} and NIL-*GSA1*^{CG14}.

a-**c**, Comparison of 1,000-grain weight (**a**), grain length (**b**) and grain width (**c**) among lines *GSA1*$^{WYJ/WYJ}$, *GSA1*$^{CG14/CG14}$ and *GSA1*$^{WYJ/CG14}$. The red boxplots indicate the lines in the segregating population with the genotype *GSA1*$^{WYJ/WYJ}$, which contains the target fragment originating from WYJ; blue box-plots indicate lines with the genotype *GSA1*$^{WYJ/CG14}$, which are heterozygous for the target fragment, and the yellow box-plots indicate lines with the genotype *GSA1*$^{CG14/CG14}$, which contain the target fragment originating from CG14. The centre line, box limits and whiskers represent mean, 25% and 75% confidence limits, and min and max values, respectively. **d**, Comparison of grain number per panicle between NIL-*GSA1*^{WYJ} and NIL-*GSA1*^{CG14}. **e**, **f**, Plant architecture (**e**) and main panicle (**f**) of NIL-*GSA1*^{WYJ} and NIL-*GSA1*^{CG14}. Scale bar, 10 cm (**e**), 5 cm (**f**), respectively. **g**-**j**, Comparison of plant height (**g**), number of effective panicles (**h**), panicle length (**i**) and grain yield per plant (**j**) between NIL-*GSA1*^{WYJ} and NIL-*GSA1*^{CG14}. The values in **d** and **g**–**j** represent the mean ± s.d. (n = 40 plants).

**$P < 0.01$ indicates significant differences compared with NIL-*GSA1*^{WYJ} in two-tailed Student's *t*-tests. Source data underlying Supplementary Figure 1a-d and 1g-j are provided as a Source Data file.

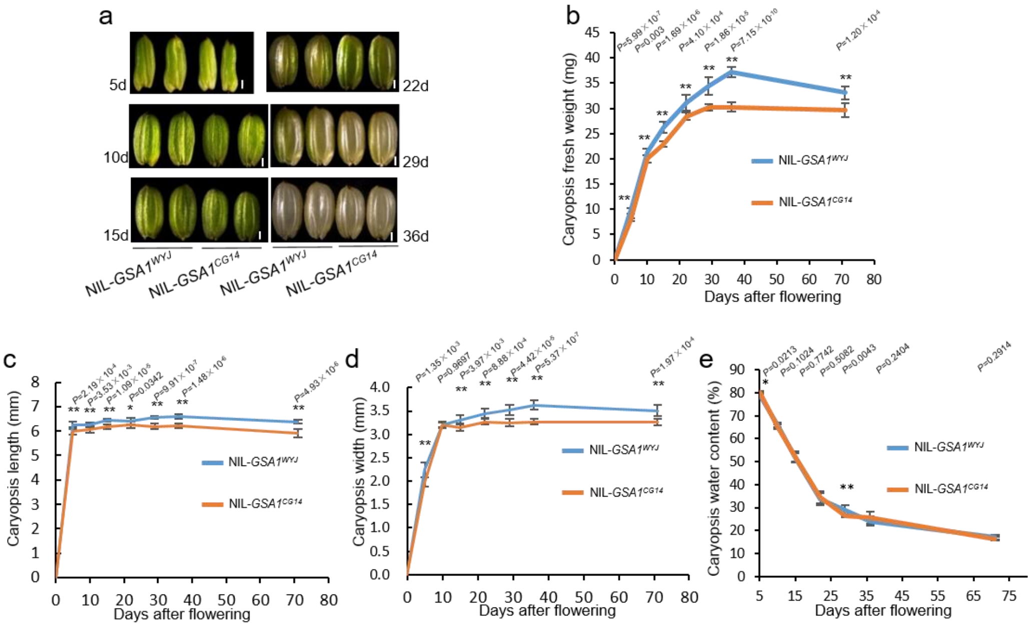

Supplementary Figure 2. Caryopsis development in NIL-*GSA1*^{WYJ} and NIL-*GSA1*^{CG14}.
a, Caryopses at 5 days, 10 days, 15 days, 22 days, 29 days and 36 days after flowering of NIL-*GSA1*^{WYJ} and NIL-*GSA1*^{CG14}. Scale bar, 1 mm. **b-e**, Time-course of the change in caryopsis fresh weight (**b**), caryopsis length (**c**), caryopsis width (**d**), caryopsis water content (**e**) of NIL-*GSA1*^{WYJ} and NIL-*GSA1*^{CG14}. The values in **b**–**e** represent the mean ± s.d. ($n = 8$ plants, 40 caryopses per plant). *$P < 0.05$ and **$P < 0.01$ indicate significant differences compared with NIL-*GSA1*^{WYJ} in two-tailed Student's *t*-tests. Source data underlying Supplementary Figure 2b-e are provided as a Source Data file.

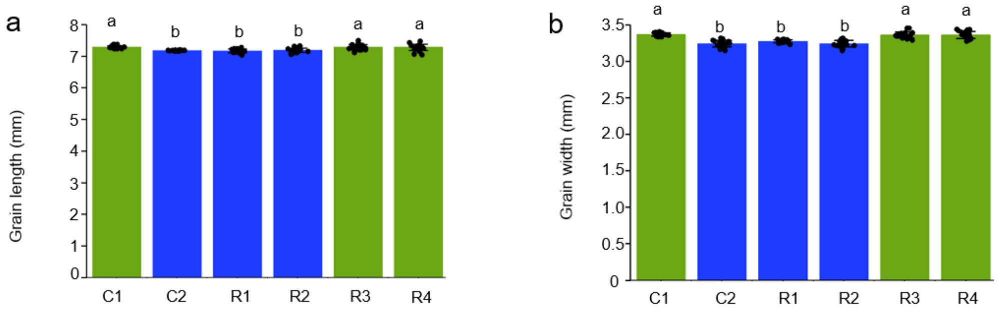

Supplementary Figure 3. Grain length and grain width of recombinant lines.

a, b, Grain length (**a**) and grain width (**b**) of representative recombinant lines C1, C2, R1, R2, R3 and R4 in Figure **1f**. The values in **a** and **b** represent the mean ± s.d. (n = 20 plants). Different letters indicate significant differences (P<0.05) determined by Duncan's multiple range test. Source data are provided as a Source Data file.

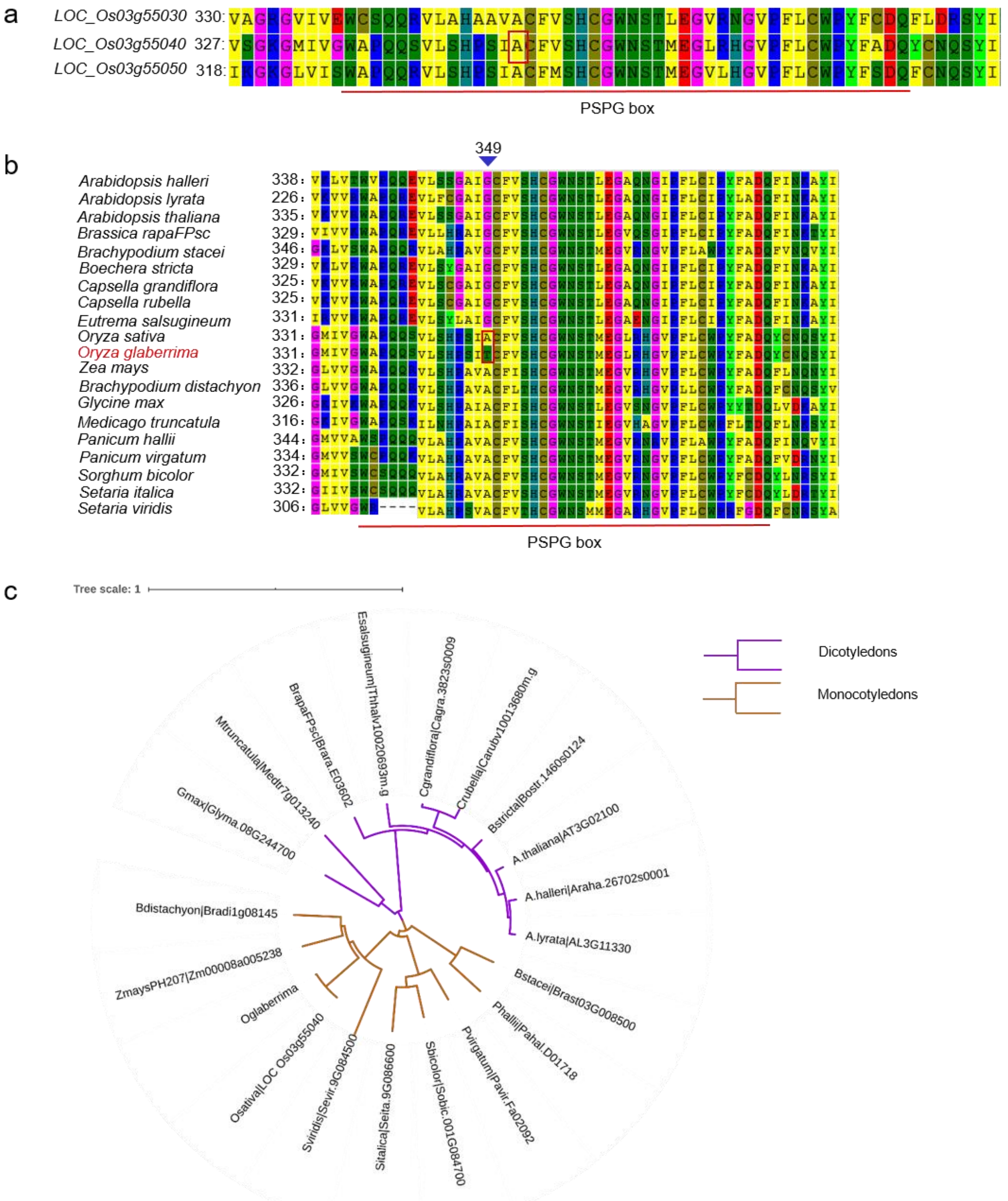

Supplementary Figure 4. Natural variation in the PSPG box of *GSA1*.

a, Alignment of partial GSA1 homologous UGT amino acid sequence in the target region. **b**, Amino acid sequence alignment of partial GSA1 homologous peptide sequences from monocots and dicots. **a**, **b**, The red line indicates the PSPG box domain. The red box indicates the substitution site (T349 in *O. glaberrima* CG14). An alanine residue at this site contained in the PSPG box is highly conserved across monocots. **c**, Neighbor-joining tree using variants in GSA1 with its homologs amino acid sequence. The brown branches and purple branches indicate monocot and dicot sequences, respectively.

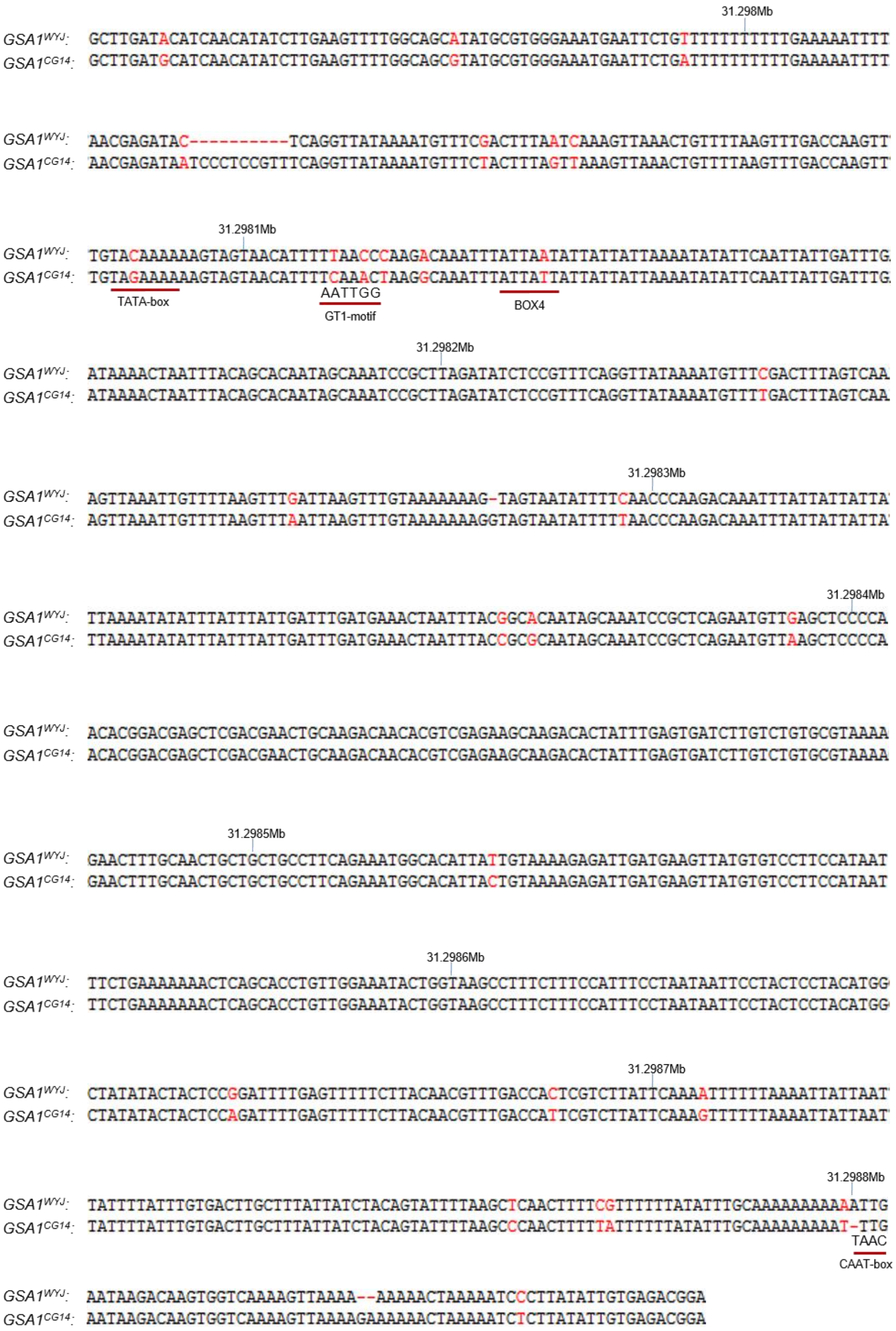

Supplementary Figure 5. Comparison of the promoter regions of *GSA1^{WYJ}* and *GSA1^{CG14}*.
a, Comparison of the promoter regions of *GSA1^{WYJ}* and *GSA1^{CG14}*. Natural variations in the promoter region are shown in red. Conserved motifs predicted by the PlantCARE database, which contain natural variations are underlined in red.

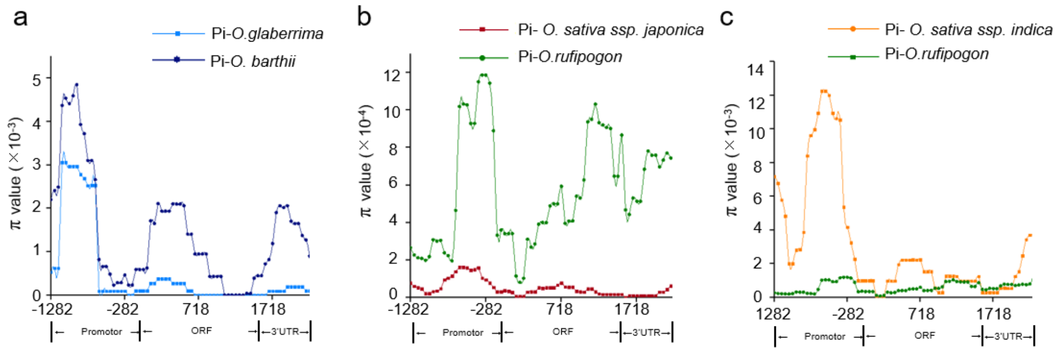

Supplementary Figure 6. Nucleotide diversity analysis of *GSA1*.

a-c, Nucleotide diversity (π) analysis of the *GSA1* promoters and entire ORF of *O.glaberrima* and *O.barthii* (**a**), *O. sativa ssp. japonica* and *O.rufipogon* (**b**) and *O. sativa ssp. indica* and *O.rufipogon* (**c**). Sliding-window analysis of the nucleotide diversity (π) was performed in a region of ~3.3kb using a 500-bp window and 50-bp steps. Source data are provided as a Source Data file.

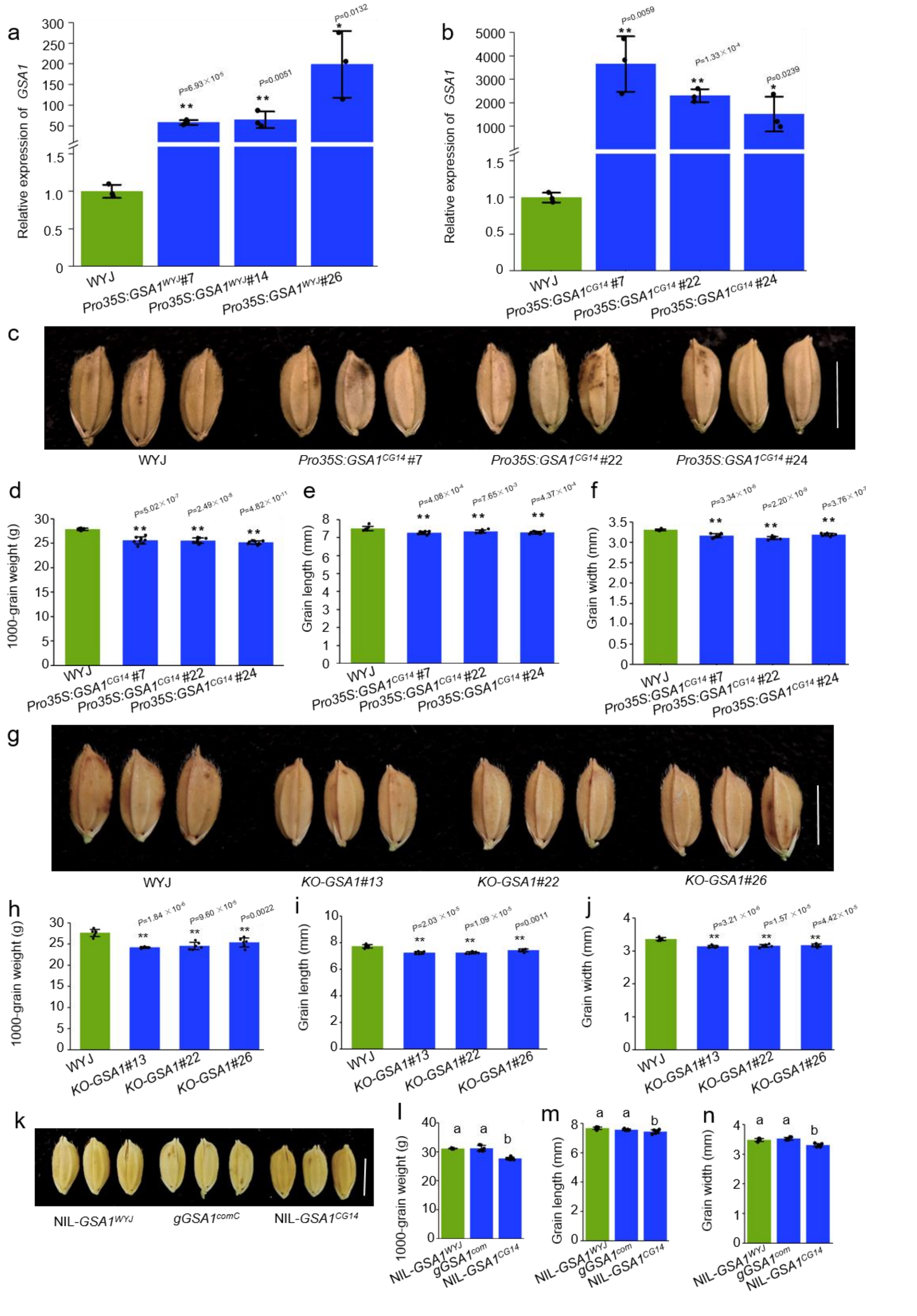

Supplementary Figure 7. Phenotypes of *GSA1* transgenic lines.

a, Relative expression levels of *GSA1* determined by qRT-PCR in WYJ and overexpression lines *Pro35S:GSA1*^{WYJ}#7, *Pro35S:GSA1*^{WYJ}#14 and *Pro35S:GSA1*^{WYJ}#26 (n = 3 biological replicates). **b**, Relative expression of *GSA1* determined by qRT-PCR in WYJ and overexpression lines *Pro35S:GSA1*^{CG14}#7, *Pro35S:GSA1*^{CG14}#22 and *Pro35S:GSA1*^{CG14}#24 (n = 3 biological replicates).

a, **b**, The ubiquitin gene was used for normalization. **c**, Mature grains of WYJ and the $GSA1^{CG14}$ overexpression lines. Scale bar, 5 mm. **d-f**, Comparison of 1,000-grain weight (**d**), grain length (**e**) and grain width (**f**) between WYJ and overexpression lines (n = 8 plants). **g**, Mature grains of WYJ and the CRISPR-Cas9 knock-out transgenic lines *KO-GSA1*#13, *KO-GSA1*#22 and *KO-GSA1*#26. Scale bar, 5 mm. **h-j**, Comparison of 1,000-grain weight (**h**), grain length (**i**) and grain width (**j**) between WYJ and knock-out transgenic lines (n = 6 plants). *P< 0.05 and **P< 0.01 indicate significant differences compared with WYJ in two-tailed Student's *t*-tests. **k**, Mature grains of NIL-$GSA1^{WYJ}$, the complementation line $gGSA1^{com}$ and NIL-$GSA1^{CG14}$. Scale bar, 5 mm. **l-n**, Comparison of 1,000-grain weight (**l**), grain length (**m**) and grain width (**n**) between NIL-$GSA1^{WYJ}$, $gGSA1^{com}$ and NIL-$GSA1^{CG14}$(n = 6 plants). Different letters indicate significant differences (P<0.05) determined by Duncan's multiple range test. The values in **a**, **b, d-f**, **h-j** and **l-n** represent the mean ± s.d.. Source data underlying Supplementary Figure 7a-b, 7d-f, 7h-j and 7l-n are provided as a Source Data file.

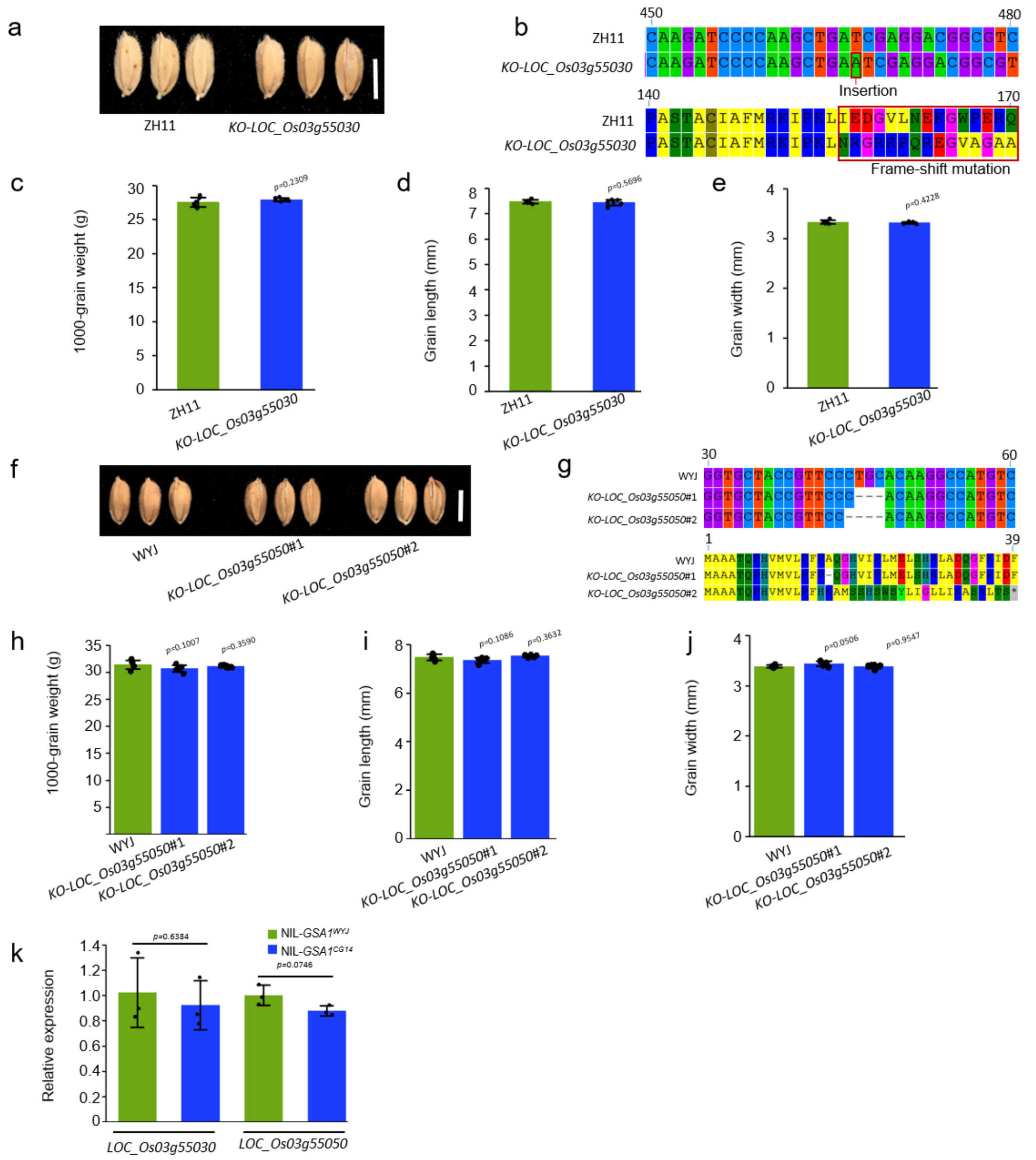

Supplementary Figure 8. The other UGT genes within the *GSA1* target region have no effect on grain size.

a, Mature grains of ZH11 and the homozygous CRISPR-Cas9 knock-out transgenic lines *KO-LOC_Os03g55030*. Scale bar, 5 mm. **b**, Alignment of the nucleotide sequences (450-480, upper panel) and amino acid sequences (140-170, lower panel) of *LOC-Os03g55030* in ZH11 and *KO-LOC_Os03g55030* showing the insertion of A466 (upper panel) and the resulting frameshift mutation (lower panel) in the mutant allele. **c-e**, Comparison of 1,000-grain weight (**c**), grain length (**d**) and grain width (**e**) between ZH11 and *KO-LOC_Os03g55030* (*n* = 6 plants). **f**, Mature grains of WYJ and the homozygous CRISPR-Cas9 knock-out transgenic lines *KO-LOC_Os03g55050*#1 and *KO-LOC_Os03g55050*#2. Scale bar, 5 mm. **g**, Alignment of the nucleotide sequences (30-60, upper panel) and amino acid sequences (1-39, lower panel) of *LOC_Os03g55050* in WYJ, *KO-LOC_Os03g55050*#1 and *KO-LOC_Os03g55050*#2 showing the mutations which result in the deletion of alanine and a frameshift causing premature termination. **h-j**, Comparison of 1,000-grain weight (**h**), grain length (**i**) and grain width (**j**) between WYJ, *KO-LOC_Os03g55050*#1 and *KO-LOC_Os03g55050*#2 (*n* = 6 plants). **k**, The relative expression

levels of *LOC_Os03g55030* and *LOC_Os03g55050* in young panicles of NIL-*GSA1*^{WYJ} and NIL-*GSA1*^{CG14} (*n* = 3 biological replicates). The actin gene was used for normalization. The values in **c-e** and **h-k** represent the mean ± s.d. *P* values in **c-e** and **h-k** from two-tailed Student's *t*-tests were indicated. Source data underlying Supplementary Figure 8c-e and 8h-k are provided as a Source Data file.

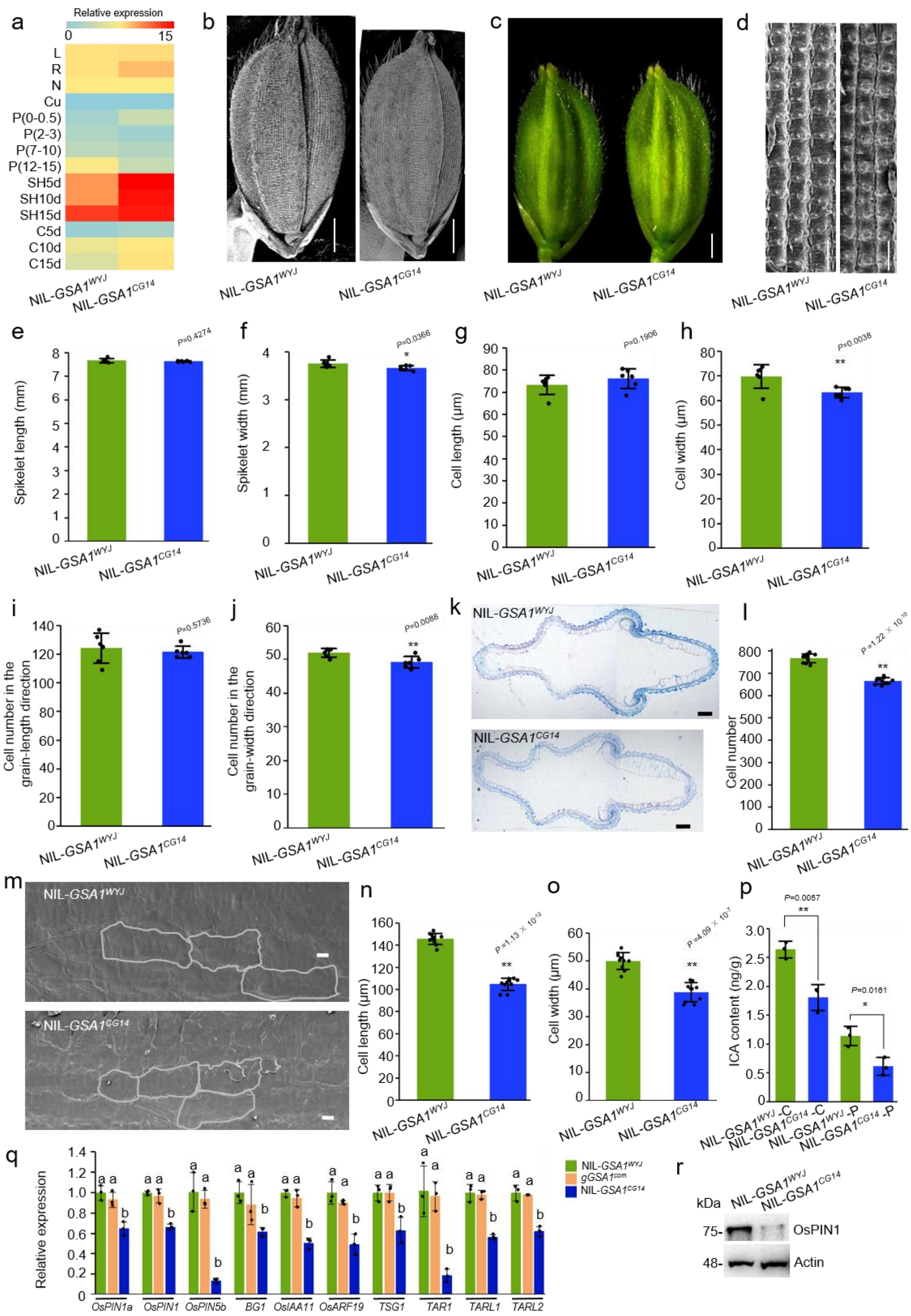

Supplementary Figure 9. Spikelet development in NIL-*GSA1*^{WYJ} and NIL-*GSA1*^{CG14}.

a, Heat map of the relative expression levels of *GSA1* in NIL-*GSA1*^{WYJ} and NIL-*GSA1*^{CG14} leaves (L), roots (R), culm nodes (N), culms (Cu), young panicles (P, numbers in brackets indicate young panicle length), spikelet hulls (SH) and caryopses (C) determined by qRT-PCR (n = 3 biological replicates). 5 d, 10 d and 15 d indicate 5 days, 10 days and 15 days after flowering. The actin gene was used for normalization. **b**, Scanning electron micrographs of the outer epidermal cells of NIL-*GSA1*^{WYJ} and NIL-*GSA1*^{CG14} spikelet hulls at the mature stage. Scale bar, 1 mm. **c**, Spikelets of NIL-*GSA1*^{WYJ} and NIL-*GSA1*^{CG14} at the booting stage. Scale bar, 1 mm. **d**, Scanning electron

micrographs of the outer epidermal cell of NIL-*GSA1*^{WYJ} and NIL-*GSA1*^{CG14} spikelet hulls at the booting stage. Scale bar, 100 µm. **e-j**, Comparisons of spikelet length (**e**), spikelet width (**f**), outer epidermal cell length (**g**), outer epidermal cell width (**h**), cell number in the grain-length direction (**i**) and cell number in the grain-width direction (**j**) between NIL-*GSA1*^{WYJ} and NIL-*GSA1*^{CG14} spikelet hulls at the booting stage (n = 6 grains). **k**, Cross sections of NIL-*GSA1*^{WYJ} and NIL-*GSA1*^{CG14} spikelet hulls. Scale bar, 200 µm. **l**, Comparisons of cell numbers in the outer parenchymal cell layers of NIL-*GSA1*^{WYJ} and NIL-*GSA1*^{CG14} spikelet hulls (n = 10 views). **m**, Scanning electron micrographs of the inner surfaces of NIL-*GSA1*^{WYJ} and NIL-*GSA1*^{CG14} spikelet hulls. Scale bar, 20 µm. **n-o**, Comparisons of inner epidermal cell length (**n**) and inner epidermal cell width (**o**) between NIL-*GSA1*^{WYJ} and NIL-*GSA1*^{CG14} spikelet hulls (n = 10 views). **p**, Comparison of the endogenous Indole-3-carboxaldehyde (ICA) levels in young caryopses (C, 10 days after flowering) and young panicles (P, ~10 cm) at the booting stage between NIL-*GSA1*^{WYJ} and NIL-*GSA1*^{CG14} (n = 3 biological replicates). The values in **e-j**, **l** and **n-p** represent the mean ± s.d. *$P < 0.05$ and **$P < 0.01$ indicate significant differences compared with NIL-*GSA1*^{WYJ} in two-tailed Student's *t*-tests. **q**, The relative expression levels of auxin related genes in 14 day old seedlings of NIL-*GSA1*^{WYJ}, *gGSA1*^{com} and NIL-*GSA1*^{CG14} determined by qRT-PCR. The values represent the mean ± s.d. (n = 3 biological replicates). Different letters indicate significant differences (P<0.05) determined by Duncan's multiple range test. **r**, Immunoblotting assays of endogenous OsPIN1 protein levels in NIL-*GSA1*^{WYJ} and NIL-*GSA1*^{CG14}. Young panicle samples were subjected to immunoblot analysis with anti-PIN1 antibody (Abiocode, 1:2,000 dilution).The loading control was visualized using the anti-actin antibody (Abmart, M20009, 1:2,000 dilution). All analyses were repeated independently at least three times. Source data underlying Supplementary Figure 9a, 9e-j, 9l and 9n-r are provided as a Source Data file.

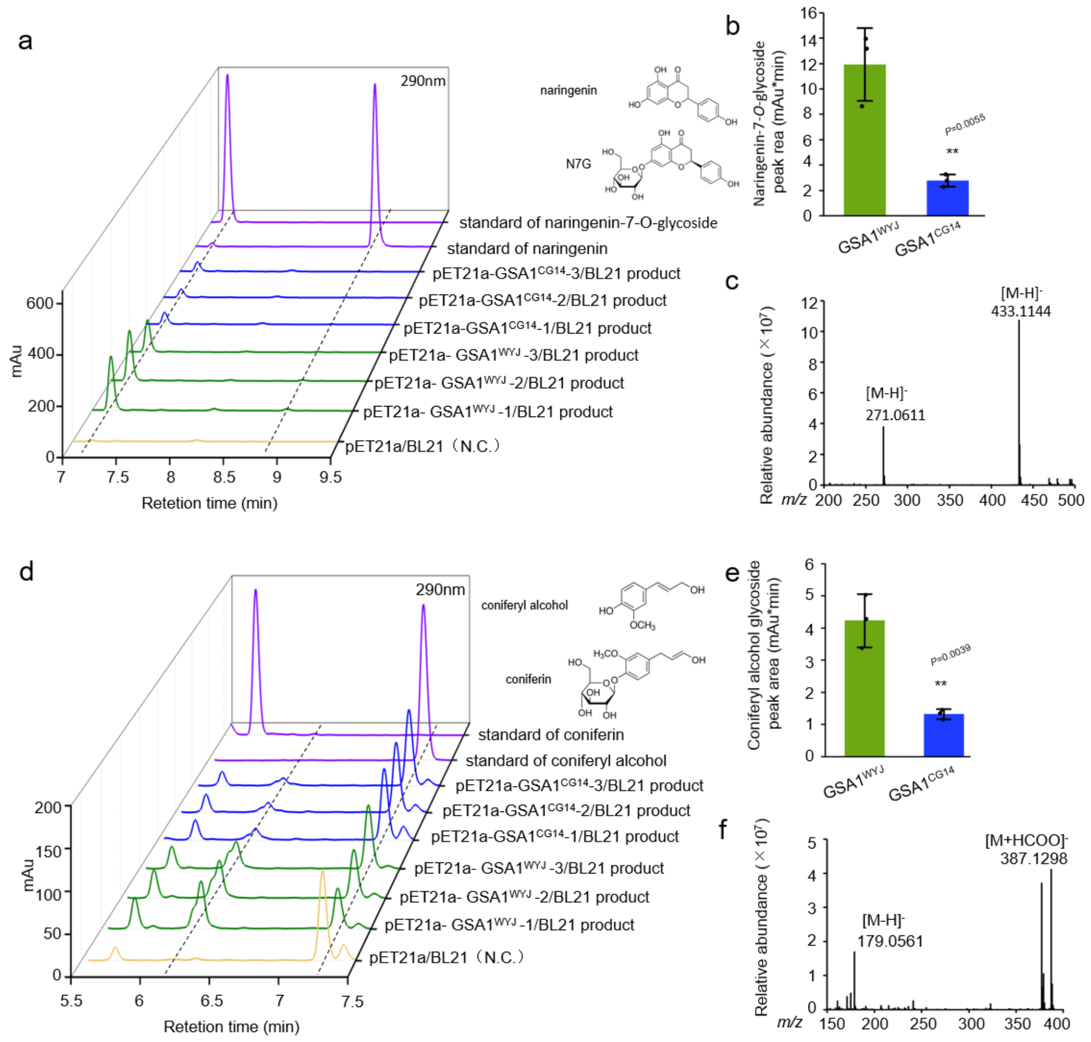

Supplementary Figure 10. Glucosyltransferase activity of GSA1 towards naringenin and coniferyl alcohol.

a, The *in vitro* glycosyltransferase activity of GSA1^{WYJ} and GSA1^{CG14} towards naringenin determined by HPLC analyses. N.C., negative control. N7G, naringenin-7-*O*-glycoside. **b**, Comparison of the peak areas of naringenin-7-*O*-glycoside produced by GSA1^{WYJ} and GSA1^{CG14}. **c**, MS analyses of the glucoside products produced by GSA1^{WYJ}. Mass spectra of naringenin consisted of a parent molecule ion with *m/z* 271.0611 [M-H]$^-$ and naringenin-7-*O*-glycoside with *m/z* 433.1144 [M-H]$^-$. **d**, The *in vitro* glycosyltransferase activity of GSA1^{WYJ} and GSA1^{CG14} towards coniferyl alcohol determined by HPLC analyses. N.C., negative control. **e**, Comparison of the peak areas of coniferyl alcohol glycoside produced by GSA1^{WYJ} and GSA1^{CG14}. The values in **b** and **e** represent the mean ± s.d. ($n = 3$ biological replicates). **$P < 0.01$ indicates significant differences compared with GSA1^{WYJ} in two-tailed Student's *t*-tests (**b**, **e**). **f**, MS analyses of the glucoside products produced by GSA1^{WYJ}. Mass spectra of coniferyl alcohol consisted of a parent molecule ion with *m/z* 179.0516 [M-H]$^-$ and a coniferin methanoic acid adduct with *m/z* 387.1298 [M+HCOO]$^-$. Source data underlying Supplementary Figure 10b and 10e are provided as a Source Data file.

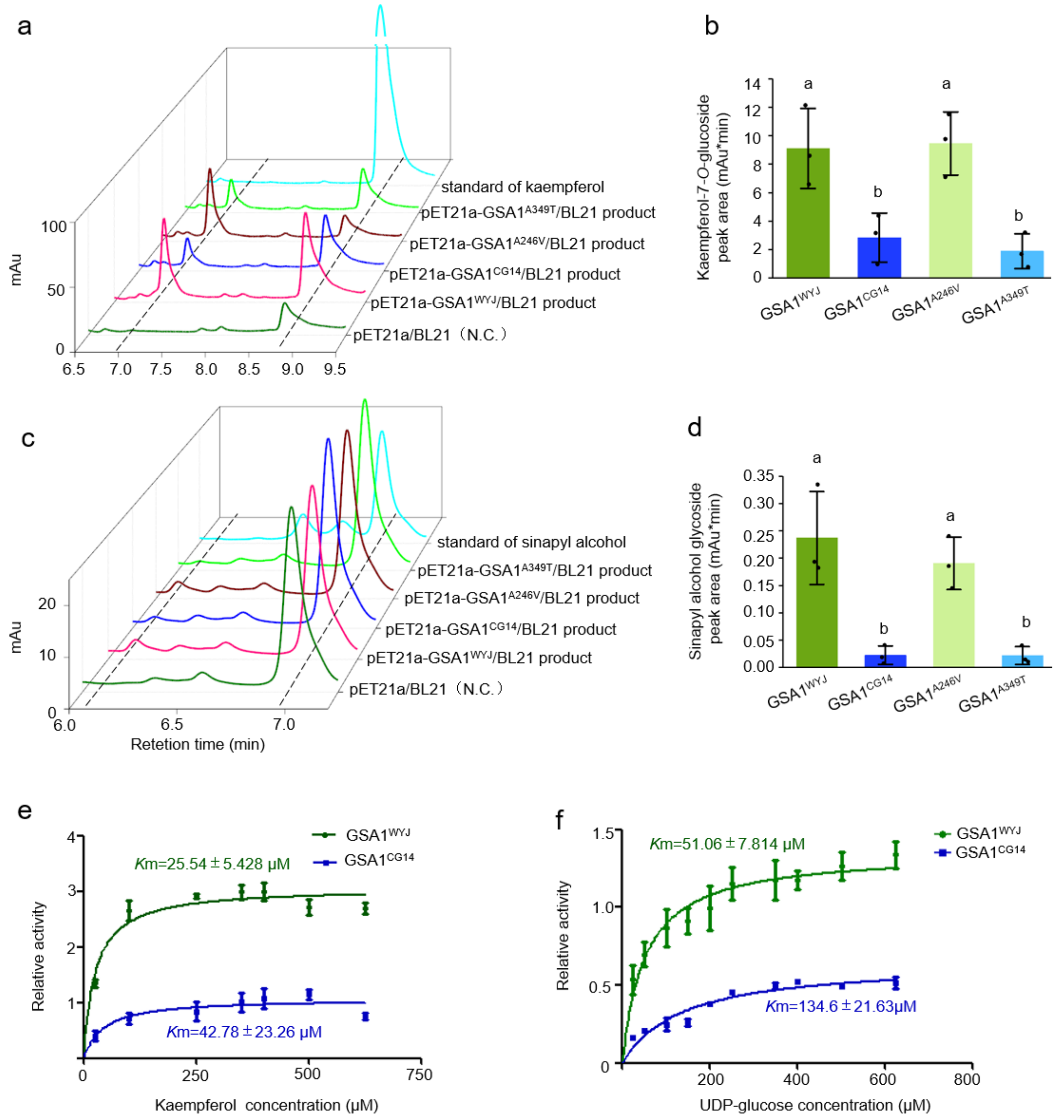

Supplementary Figure 11. Identification of the key amino acids influencing glucosyltransferase activity of GSA1 and analysis of the GSA1 enzyme kinetics.

a, The *in vitro* glycosyltransferase activity of $GSA1^{WYJ}$, $GSA1^{CG14}$, $GSA1^{A246V}$ and $GSA1^{A349T}$ towards kaempferol determined by HPLC analyses. N.C., negative control. **b**, Comparison of the peak areas of kaempferol-7-*O*-glycoside produced by $GSA1^{WYJ}$, $GSA1^{CG14}$, $GSA1^{A246V}$ and $GSA1^{A349T}$. **c**, The *in vitro* glycosyltransferase activity of $GSA1^{WYJ}$, $GSA1^{CG14}$, $GSA1^{A246V}$ and $GSA1^{A349T}$ towards sinapyl alcohol determined by HPLC analyses. N.C., negative control. **d**, Comparison of the peak areas of sinapyl alcohol glycoside produced by $GSA1^{WYJ}$, $GSA1^{CG14}$, $GSA1^{A246V}$ and $GSA1^{A349T}$. The values in **b** and **d** represent the mean ± s.d. (n = 3 biological replicates). Different letters indicate significant differences (P<0.05) determined by Duncan's multiple range test. **e**, **f**, Characterization of GSA1 enzymatic activity. The kinetic parameter *Km* of GSA1 towards kaempferol (**e**) and UDP-glucose (**f**) using kaempferol as sugar acceptor was calculated using GraphPad Prism 5 software. The values in **e** and **f** represent the mean ± s.d. (n = 3 biological replicates). The UDP-glucose was excess (2.5 mM) (**e**). The kaempferol was excess (2.5 mM) (**f**). Source data underlying Supplementary Figure 11b and 11d-f are provided as a Source Data file.

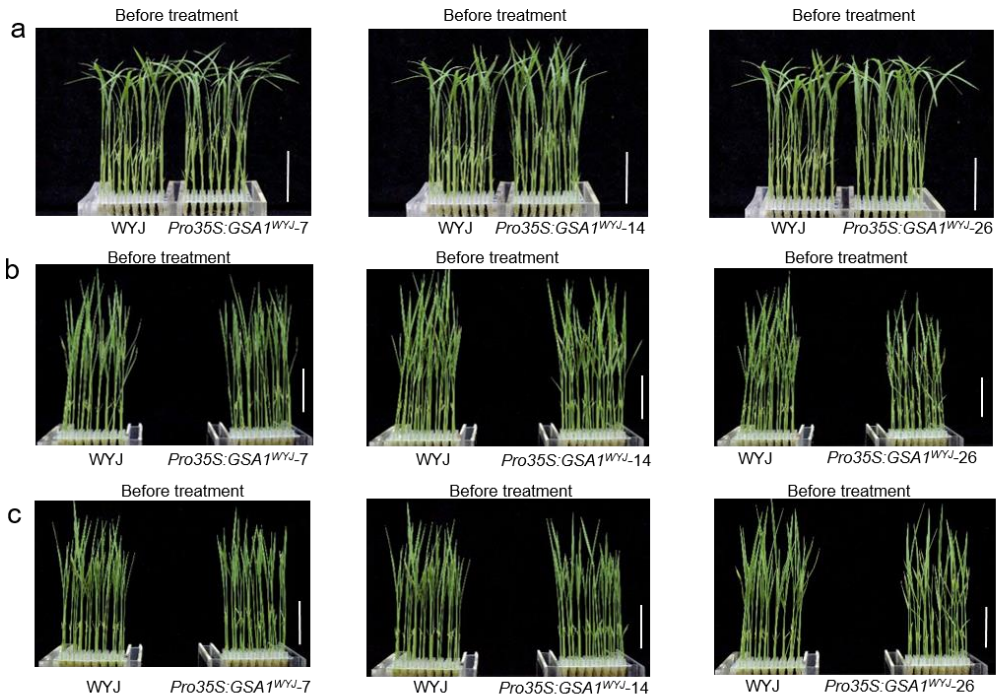

Supplementary Figure 12. WYJ and overexpression lines before abiotic stress treatment. **a-c**, WYJ and overexpression lines grown under normal conditions for 14 days before NaCl treatment (**a**), heat treatment (**b**) and PEG treatment (**c**). At least three biological replicates (24 plants per biological replicate) were used in **a-c**. Scale bar, 5 cm.

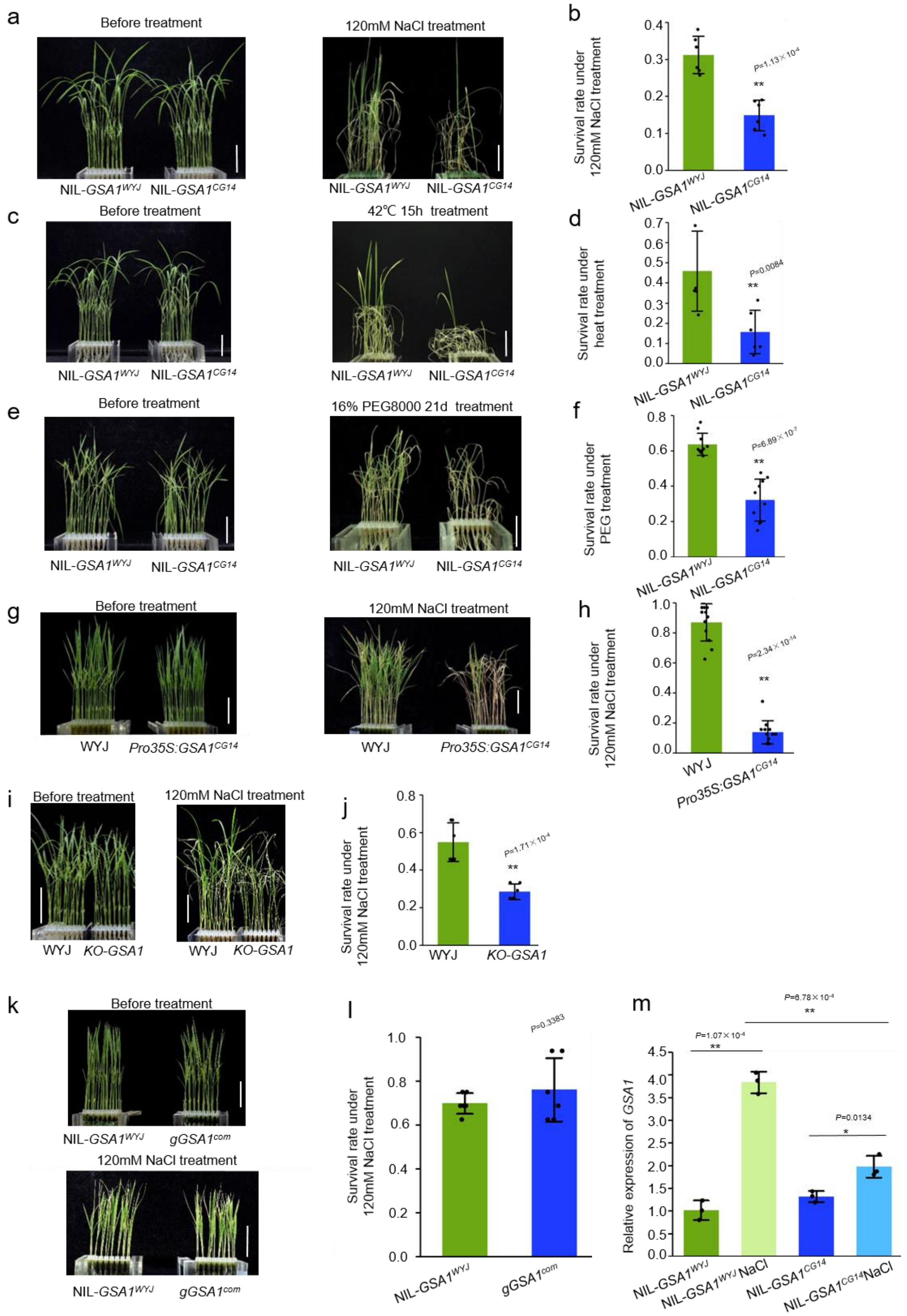

Supplementary Figure 13. ***GSA1*** **is involved in abiotic stress tolerance**

a, NIL-*GSA1*^{WYJ} and NIL-*GSA1*^{CG14} plants grown under normal conditions for 14 days (left), then transferred to 120 mM NaCl for 7 days and recovered for 7 days (right). Scale bar, 5 cm. **b**, Relative survival rates of NIL-*GSA1*^{WYJ} and NIL-*GSA1*^{CG14} seedlings after NaCl treatment (n = 6 biological replicates). **c**, NIL-*GSA1*^{WYJ} and NIL-*GSA1*^{CG14} plants grown under normal conditions

for 14 days (left), then transferred to 42ºC for 15 hours and recovered for 7 days (right). Scale bar, 5 cm. **d**, Relative survival rates of NIL-*GSA1*^{WYJ} and NIL-*GSA1*^{CG14} seedlings after heat treatment (*n* = 6 biological replicates). **e**, NIL-*GSA1*^{WYJ} and NIL-*GSA1*^{CG14} plants grown under normal conditions for 14 days (left), then transferred to 16% PEG for 21 days and recovered for 7 days (right). Scale bar, 5 cm. **f**, Relative survival rates of NIL-*GSA1*^{WYJ} and NIL-*GSA1*^{CG14} seedlings after PEG treatment (*n* = 10 biological replicates). **b**, **d**, **f**, 24 plants per biological replicate. **g**, WYJ and *Pro35S:GSA1*^{CG14} grown under normal conditions for 14 days before NaCl treatment (left), then transferred to 120 mM NaCl for 7 days and recovered for 7 days (right). Scale bar, 5 cm. **h,** Relative survival rates of WYJ and *Pro35S:GSA1*^{CG14} seedlings after NaCl treatment (*n* = 12 biological replicates, 32 plants per biological replicate). **i,** WYJ and *KO-GSA1* grown under normal conditions for 14 days before NaCl treatment (left), then transferred to 120 mM NaCl for 7 days and recovered for 7 days (right). Scale bar, 5 cm. **j,** Relative survival rates of WYJ and *KO-GSA1* seedlings after NaCl treatment (*n* = 6 biological replicates, 24 plants per biological replicate). **k,** NIL-*GSA1*^{WYJ} and *gGSA1*^{com} grown under normal conditions for 14 days before NaCl treatment (upper), then transferred to 120 mM NaCl for 7 days and recovered for 7 days (lower). Scale bar, 5 cm. **l,** Relative survival rates of NIL-*GSA1*^{WYJ} and *gGSA1*^{com} seedlings after NaCl treatment (*n* = 6 biological replicates, 16 plants per biological replicate). **m**, The relative expression levels of *GSA1* before and after NaCl treatment for 5 days in NIL-*GSA1*^{WYJ} and NIL-*GSA1*^{CG14} (*n* = 3 biological replicates). The ubiquitin gene was used for normalization. The values in **b**, **d**, **f**, **h**, **j, l** and **m** represent the mean ± s.d. **P*< 0.05 and ***P*< 0.01 indicates significant differences compared with NIL-*GSA1*^{WYJ} or WYJ in two-tailed Student's *t*-tests. Source data underlying Supplementary Figure 13b, 13d, 13f, 13h, 13j, 13l and 13m are provided as a Source Data file.

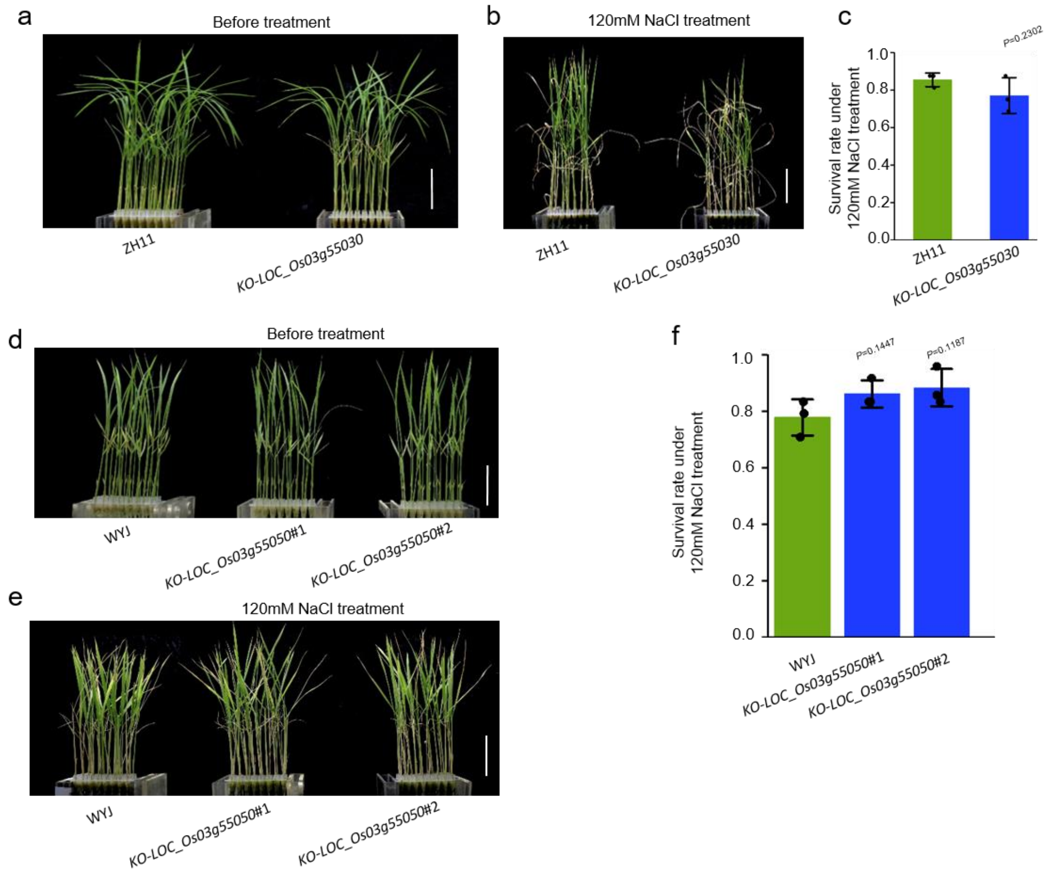

Supplementary Figure 14. The other UGT genes within the *GSA1* target region have no effect on abiotic stress tolerance.

a, ZH11 and the *LOC_Os03g55030* knock-out line *KO-LOC_Os03g55030* grown under normal conditions for 14 days before NaCl treatment, Scale bar, 5 cm. **b**, ZH11 and *KO-LOC_Os03g55030* transferred to 120 mM NaCl for 7 days and recovered for 7 days. Scale bar, 5 cm. **c**, Relative survival rates of ZH11 and *KO-LOC_Os03g55030* seedlings after NaCl treatment (n = 3 biological replicates, 16 plants per biological replicate). **d**, WYJ and *LOC_Os03g55050* knock-out lines *KO-LOC_Os03g55050*#1 and #2 grown under normal conditions for 14 days before NaCl treatment, Scale bar, 5 cm. **e**, WYJ and *KO-LOC_Os03g55050*#1 and #2 transferred to 120 mM NaCl for 7 days and recovered for 7 days. Scale bar, 5 cm. **f**, Relative survival rates of WYJ and *KO-LOC_Os03g55050*#1 and #2 seedlings after NaCl treatment (n = 3 biological replicates, 24 plants per biological replicate). The values in **c** and **f** represent the mean ± s.d. P values in **c** and **f** from two-tailed Student's t-tests were indicated. Source data underlying Supplementary Figure 14c and 14f are provided as a Source Data file.

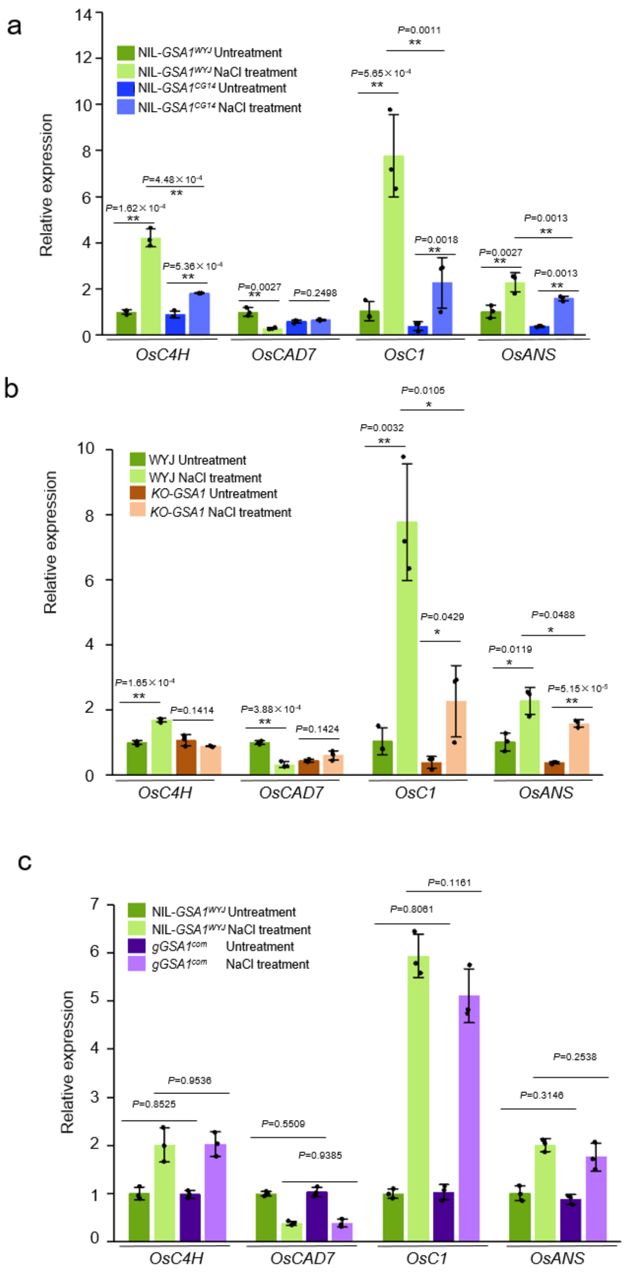

Supplementary Figure 15. *GSA1* is required for the activation of flavonoid biosynthesis and down-regulation of the lignin pathway under abiotic stress.

a, The relative expression levels of *OsC4H*, *OsCAD7*, *OsC1* and *OsANS* determined by qRT-PCR before and after NaCl treatment in NIL-$GSA1^{WYJ}$ and NIL-$GSA1^{CG14}$ seedlings (n = 3 biological replicates). **b**, The relative expression levels of *OsC4H*, *OsCAD7*, *OsC1* and *OsANS* determined by qRT-PCR before and after NaCl treatment in WYJ and *KO-GSA1* seedlings (n = 3 biological replicates). **c**, The relative expression levels of *OsC4H*, *OsCAD7*, *OsC1* and *OsANS* determined by qRT-PCR before and after NaCl treatment in NIL-$GSA1^{WYJ}$ and $gGSA1^{com}$ seedlings (n = 3 biological replicates). The ubiquitin gene was used for normalization. The values in **a-c** represent the mean ± s.d. *P< 0.05 and **P< 0.01 indicates significant differences compared with NIL-$GSA1^{WYJ}$ or WYJ in two-tailed Student's *t*-tests. Source data are provided as a Source Data file.

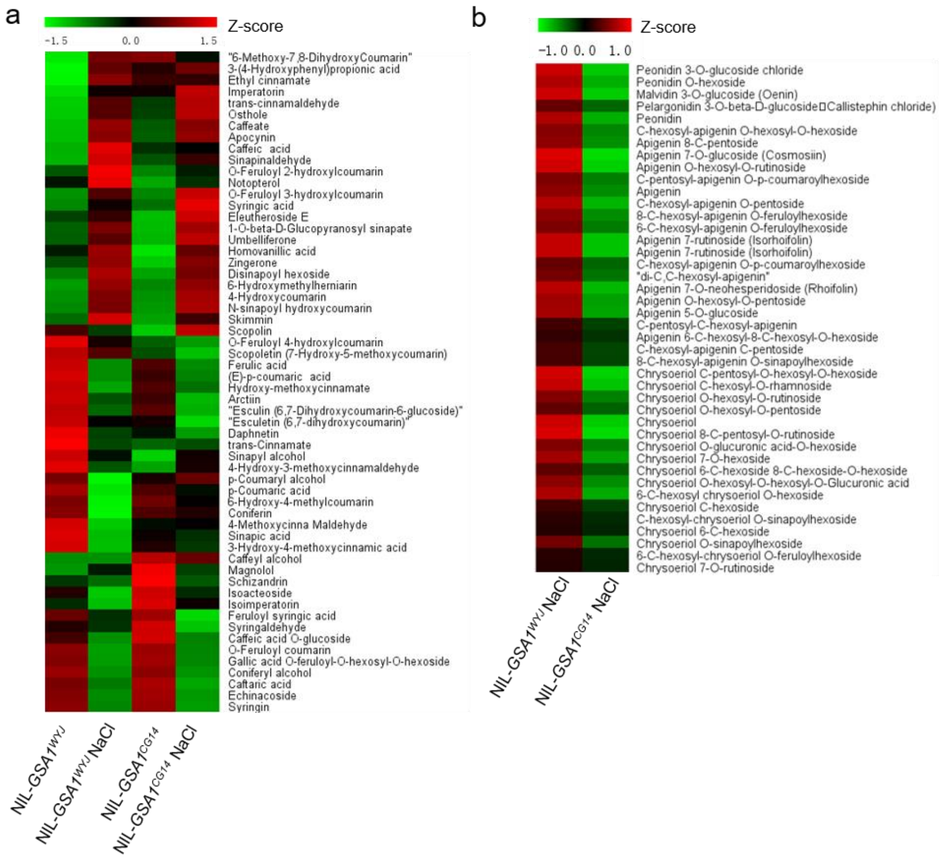

Supplementary Figure 16. The redirection of metabolic flux after NaCl treatment in NIL-*GSA1*^{WYJ} and NIL-*GSA1*^{CG14}.

a, Clustering heat maps of the relative levels of phenylpropanoid metabolites including lignin pathway metabolites in NIL-*GSA1*^{WYJ} and NIL-*GSA1*^{CG14} seedlings before and after NaCl treatment. **b**, Clustering heat maps of the relative levels of anthocyanins, apigenin derivatives and chrysoeriol derivatives in NIL-*GSA1*^{WYJ} and NIL-*GSA1*^{CG14} seedlings after NaCl treatment. **a**, **b**, Standard-scores (Z-scores) were used as the numerical signs to evaluate the standard deviations from the mean of the corresponding samples. Source data are provided as a Source Data file.

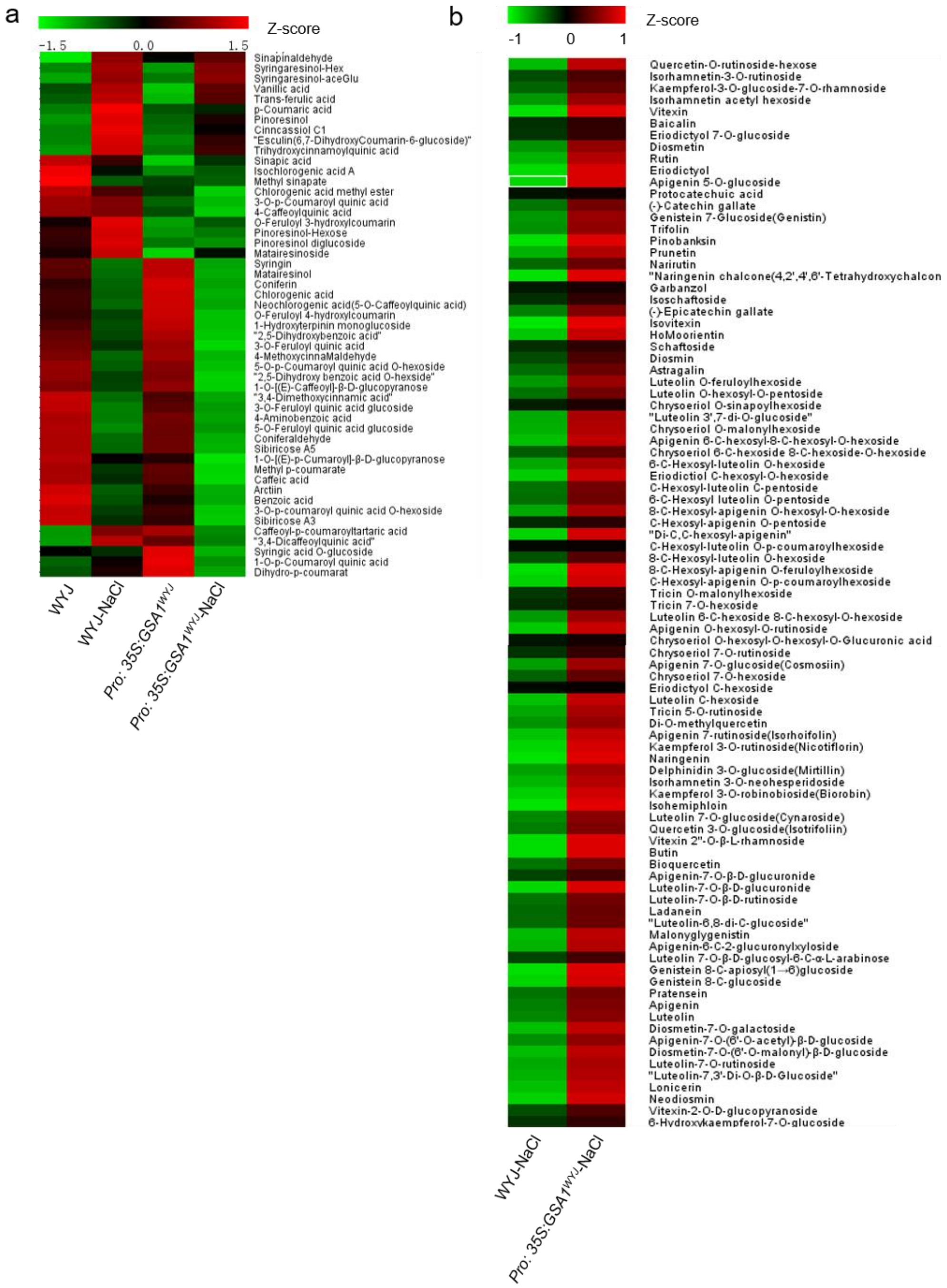

Supplementary Figure 17. The redirection of metabolic flux after NaCl treatment in WYJ and *Pro35S:GSA1*^{WYJ}.

a, Clustering heat maps of the relative levels of phenylpropanoid metabolites including lignin pathway metabolites in WYJ and *Pro35S:GSA1*^{WYJ} seedlings before and after NaCl treatment. **b**, Clustering heat maps of the relative levels of flavonoid glycosides in WYJ and *Pro35S:GSA1*^{WYJ} seedlings after NaCl treatment. **a**, **b**, Standard-scores (Z-scores) were used as the numerical signs to evaluate the standard deviations from the mean of the corresponding samples. Source data are provided as a Source Data file.

Supplementary Table 1. *GSA1* is a QTL contributing to grain size.

	LOD	%VAR	Additive effect	Molecular marker
1,000-grain weight	5.734	14.5%	-0.755	D3-125.1~D3-125.48
grain length	7.570	18.6%	-0.074	D3-125.1~D3-125.48
grain width	5.567	14.1%	-0.029	D3-125.1~D3-125.48

Supplementary Table 2. Mutation sites in *KO-GSA1* lines.

Lines	KO-*GSA1#13*	KO-*GSA1#22*	KO-*GSA1#26*
Mutation sites	Deletion:79-113	Deletion:72-79<br>Deletion:115	Deletion:79-113

Supplementary Table 3. Primers used in this study.

Primer name	Sequence 5'-3'	Purpose
D3-122.1-F	TGTAAATGAACGATGCAAGC	Map-based cloning
D3-122.1-R	TGTTGACAACGAGCTAATCA	Map-based cloning
D3-122.11-F	GGTAATAACACATGCCATCG	Map-based cloning
D3-122.11-R	AGGTTACCTCTGCTTTATTTGA	Map-based cloning
D3-124.1-F	TGGCCACTGAATGAATAACT	Map-based cloning
D3-124.1-R	CCAGTGATGGTGGTGTTAAT	Map-based cloning
D3-124.11-F	CTGTTTGGAACTTTAGGGAC	Map-based cloning
D3-124.11-R	ATACGCCTGAGGTAATCTTG	Map-based cloning
D3-125.1-F	GCAAGATGGCAAAGTCGC	Map-based cloning
D3-125.1-R	CTTTTCAGTCACATCGTATTAAT	Map-based cloning
D3-125.48-F	TATCGGATGGTCTAATCAGC	Map-based cloning
D3-125.48-R	AGGTCAGGACTCATGATCAT	Map-based cloning
D3-125.8-F	ATTTGGGGTGCTACTCAGAT	Map-based cloning
D3-125.8-R	AAAGACATCTACGGGCATATT	Map-based cloning
D3-125.107-F	ATGCTCCTGTTGTCATTCTT	Map-based cloning
D3-125.107-R	GGGCAAATACCAACATTGAT	Map-based cloning
D3-125.112-F	TACTTATGCCCGCTATAGGA	Map-based cloning
D3-125.112-R	GCAACATAAACATCGAGAAATG	Map-based cloning
D3-125.125-F	GATCAAGATGACACAACCCT	Map-based cloning
D3-125.125-R	TGCATGCATGTATGTACGAT	Map-based cloning
D3-125.18-F	AGTTTGCAGTCTGTGAGATT	Map-based cloning
D3-125.18-R	CTGACAACCATCCTTACCAC	Map-based cloning
D3-125.37-F	GGCGACATGGATTGAGATC	Map-based cloning
D3-125.37-R	GTTGAAACTAACCGGAGAAT	Map-based cloning
D3-125.49-F	CTGCATTATTGTTAATATTACGG	Map-based cloning
D3-125.49-R	CCTATGTAAGAGACTGCACA	Map-based cloning
1306-*GSA1*-R	ATCCAAGGGCGAATTGGTCGACTCTAGACTGTTCTCTT AGCAAATTCA	Transgenic construct

1306-*GSA1*-F	AGAGAACACGGGGGACGAGCTCGGTACCATGGCGGC TCCTCCTCCTCC	Transgenic construct
KO-GSA1-1F	GGCAGGCCATGTCATGCCTCTGA	Transgenic construct
KO-GSA1-1R	AAACTCAGAGGCATGACATGGCC	Transgenic construct
KO-GSA1-2F	GCCGCTCGTCGGCCTCGGCTTCG	Transgenic construct
KO-GSA1-2F	AAACCGAAGCCGAGGCCGACGAG	Transgenic construct
q*GSA1*-F	CGCACAGGCTGCAAGTC	qPCR
q*GSA1*-R	GTGGGTCTCAATTGGATCATCTC	qPCR
q030-F	GAACTCGACGCTGGAAGG	qPCR (*LOC_Os03g55030*)
q030-R	CACACGGCGGTGATGTAG	qPCR (*LOC_Os03g55030*)
q050-F	CCGTTCTTATGCTGGCCTTAT	qPCR (*LOC_Os03g55050*)
q050-R	GTTCTTGATCTCCTCCTGTGTG	qPCR (*LOC_Os03g55050*)
PAL4-F	CTTCACAACAGCTAATCGAG	qPCR
PAL4-R	CGCACTCCATTTCAGTACCA	qPCR
OsC4H-F	GCAAGAGAGTGATGGAACAGA	qPCR
OsC4H-R	CGTTGATGTTCTCGACGATGTA	qPCR
COMT-F	GAAGGTGGTGGTGGTGGAGT	qPCR
COMT-R	GCGTTGGCGTAGATGTAGGTG	qPCR
CCR1-F	CTCATCCGTGGCTACCACGTC	qPCR
CCR1-R	GGGTAGGACTTCTTGGTGCC	qPCR
OsCAD7-F	TAGCCAGCTAGCAACCGACAA	qPCR
OsCAD7-R	CCGCCGGAGCAGTAGTTCT	qPCR
F3`H-F	ATACCATTCGGAGCAGGA	qPCR
F3`H-R	TGGCAGTCATCAGTGTGA	qPCR
CHS-F	TTGGGCTGGACAAGGAGAGGATGA	qPCR
CHS-R	AGGACGACGGTCTCAACGGTGA	qPCR
CHI-F	CAGTACTCGGACAAGGTGAC	qPCR
CHI-R	GGAGTGGGTGAAGAGGAT	qPCR
OsC1-F	GAATTAGGAGACGGCGATGA	qPCR
OsC1-R	TCACGCACACAAGTTCCA	qPCR
ANS-F	CAGCTCAAGATCAACTACTAC	qPCR
ANS-R	GTGGAGGATGAAGGAGAG	qPCR
OsP1-F	TGGATCAACTACCTGAGA	qPCR
OsP1-R	ATGGAGCTTGATGATGAC	qPCR
*OsPIN1*a-F	TCATCTGGTCGCTCGTCTGC	qPCR
OsPIN1a-R	CGAACGTCGCCACCTTGTTC	qPCR
OsPIN1-F	TGCACCCTAGCATTCTCAGCA	qPCR
OsPIN1-R	CCCTCCTCCCAAATTCTACTTC	qPCR
OsPIN5b-F	GGGCAGCAGGAGAGGGTGATAG	qPCR
OsPIN5b-R	GAATCGGCAGAGAGATCAATGT	qPCR
BG1-F	GATGGAGAGCGACGAGGAC	qPCR
BG1-R	GCAATGGCGGCGAAGTTC	qPCR
OsIAA11-F	GCGCTGGTGAAGGTGAGCAT	qPCR
OsIAA11-R	ACGTACTCCAGGTCATCTCT	qPCR

OsARF19-F	TCAATGAGTTAGCAAAGGC	qPCR
OsARF19-R	TTTGATTTTGGACAAGGAC	qPCR
TSG1-F	ATAGGGAGGATGTGGAGGATT	qPCR
TSG1-R	AGAGGCGGTTGATGAAGATG	qPCR
TAR1-F	CTCTTCACCGTCTCCAAGTG	qPCR
*TAR*1-R	GATGGTGTTGAGCTCGATGA	qPCR
TARL1-F	AACCGGATCAAAGAGCCATC	qPCR
TARL1-R	CTTCGGCCTCGGATAAAGTG	qPCR
TARL2-F	AAGACGACGACTGCTACGA	qPCR
TARL2-R	CAAATTCGGTGATCCTCTCCAG	qPCR
ACTIN-F	TGCTATGTACGTCGCCATCCAG	qPCR(LOC_Os03g50885)
ACTIN-R	AATGAGTAACCACGCTCCGTCA	qPCR(LOC_Os03g50885)
UBIQUITIN-F	GACGGACGCACCCTGGCTGACTAC	qPCR(LOC_Os03g13170)
UBIQUITIN-R	TGCTGCCAATTACCATATACCACGAC	qPCR(LOC_Os03g13170)
