# Supplementary material for: UDP-glucosyltransferase regulates grain size and abiotic stress tolerance associated with metabolic flux redirection in rice
Source: Nat Commun. 2020 May 26;11:2629. doi: 10.1038/s41467-020-16403-5 (PMC7250897; doi:10.1038/s41467-020-16403-5)
Supplement: Supplementary file 2 — Peer Review File [file 41467_2020_16403_MOESM2_ESM.pdf]

Reviewers' comments:

Reviewer #1 (Remarks to the Author):

The authors reported the cloning and characterization of a minor QTL that affected both grain size and abiotic stress tolerance in rice. The minor QTL, GSA1, encodes a UDP-glucosyltransferase (UGT83A1) that exhibits glucosyltransferase activities with broad substrate specificities towards flavonoids and monolignols. The authors further conclude that natural variation of GSA1 contribute to the variation of grain size in rice and GSA1 controls grain size through regulating auxin biosynthesis, transportation, and signaling. Although the results are overall interesting, with the concerns listed below, I am not sure the conclusions drawn by the authors are fully supported by the data presented.

Main points:
1. Line 37-141: in addition to the coding sequence analyzed, the authors need to compare the promoter region between the two parents to find the possible polymorphism underlying the differential expression of the two GSA1 alleles.
2. Line 163-171: in addition to show that overexpression of GSA1WYJ in WYJ background affecting grain size, the authors really need to compliment the GSA1CG14 phenotype by introducing the genomic region of either GSA1WYJ or GSA1CG14 into CG14 background and to perform further experiments using these two lines.
3. Line 168-169: considering the GSA1CG14 is a weak allele in terms of glucosyltransferase activity, it is not reasonable the overexpressing lines resulted in smaller grain size. The data from the two isogenic lines, the two complimentary lines as required above in main points #2 should also be included.
4. Line 179-180: please give evidence for the suggestion.
5. Line 237-239: the authors need to give direct evidence that differential IAA biosynthesis, transportation, and signaling are really responsible for the variation of grain size in the two parents. I think at least data from complimentary experiment and genetic evidence need to be provided.
6. Line 335-338: kinetics of the two GSA1 alleles need to be provided.
7. Line 379-380: the sensitivity of GSA1CG14 ox line need to be included. The data from the two isogenic lines, the two complimentary lines as required above in main points #2 should also be included.
8. Line 400-402: the authors need to provide data whether and to what extend GSA1WYJ and/or GSA1CG14 are induced by abiotic stresses.
9. Line 427-428: the gene expression and metabolite profiling data showed by included for GSAl CRISPR lines and also the two complimentary lines as required above in main points #2.
10. The authors need to provide evidence that flavonoid aglycones suppress auxin pathway while glycosylated flavonoids do not.

Minor points:
1. Line 110-111: the authors need to show the result of these QTL analyses in Supplementary figure.
2. Line 115-118: the authors need to add result of QTL mapping for number of grains per panicle to show whether the GSA1 locus can also be detected by this trait.
3. Line 119-120: the authors showed that GSA1 didn't affect grain yield, however this is not consistent with what it is claimed in the Title and the Abstract.
4. Line 231-236: are TSG1 and their homologs somehow related to auxin pathway?
5. Lin 270-212: what about the flavonoid content in the leaves of the two isogenic lines and also in the complimentary lines as indicated in main points #2.
6. Line 245-247: the authors need to include the phylogenetic analysis of GSA1 with its homologs in rice and other plant species.

Reviewer #2 (Remarks to the Author):

I really enjoyed reading this. The authors have conducted a thorough analysis of GSA1 and the presentation is very clear. The authors present a study where they mapped a UGT-glucosyl transferase, UGT83A1/GSA1, in rice. This is a minor QTL controlling grain that was not previously mapped. The authors found that GSA1 not only glycosylated many flavonoid species, but also documented two other points: 1) grain size changes were due to cell length and cell number, and 2) overexpression of GSA1 activity also increased abiotic stress tolerance. The authors conclude that GSA1 activity results in metabolic flux changes in the phenyl propanoid pathway and propose that the mechanism is via a feedback loop. However, the author conclude in several places that GSA1 also directly controls auxin biosynthesis, but how GSA1 directly acts on auxin biosynthesis is not clear. There are a few minor comments about some of the interpretations and presentation of the conclusions that need to be addressed.
1.The authors have presented one interpretation of the data, and a in a few cases, the authors may consider adding an alternate hypothesis or alternate interpretation of their data. I encouraged the authors to expand a bit more on the other possibilities.
2. One example is alluded to above regarding GSA1 control of auxin biosynthesis. Since does not encode an auxin biosynthetic enzyme and or a transcription factor, direct control is unlikely. The effect may be indirect. The gene expression of the auxin responsive genes make sense given the auxin levels. The auxin levels and expression of auxin metabolic genes and transporters appear match in the two cultivars, respectively. Perhaps gene expression is constitutively low in CG14 overall? This appears to be the case generally in the data presented. However, there does appear to be a case where CG14 has increased expression in YP compared to WYJ (Fig. 3a). Can this helpful in interpreting your data?
3. Grain cell number (cell division) is also regulated by cytokinin, so this is also a possible mechanism in addition to the role of auxin in cell elongation. Lines 234-237, Lines 495, etc, make a definitive statement, that is extrapolating the data too far, in my opinion. Can the authors modify these sentence? The characterization in lines 520-521 more in keeping with what the date show. Also, Fig 6 does not show GSA1 acting directly on auxin, which is consistent with what the authors state in lines 520-521 and to my thinking.
4. Since other UGTs are in the QTL and a gene of unknown function, and there is more scopolin, heavily glucosylated apigenen, etc., in CG14, is it possible that CG14 might have some other compensatory mechanism for a partially active GSA1 that increases these glucosylated compounds? Are there any differences/natural variation between GC14 and WJY besides GSA1 known? Is it possible that has overall lower gene expression than in WYJ?
5. Fig. S6d, e. For the image it appears that the empty vector (negative control) is the same as CG14? Was the baseline (negative control) subtracted from all of the data? That may affect the presentation of these data if CG14 is equivalent to negative control. Can that authors add a sentence that these are in vitro assays, and that some of the reactions may not necessarily occur in in vivo if the enzyme and substrates are not in the same cell type or compartment?
6. Lines 392-394. While I believe that you can make this statement for the phenylpropanoid pathway because substrate shunting and the feedback mechanism is largely known, I do not agree that this same logic can be applied to auxin biosynthesis when it is applied in other places in this work.
7. Lines 399-400. I think that concluding that GSA1 is the key regulator of lignin biosynthesis under abiotic stress is also an overstatement, especially as CAD7 expression is not affected in bi biotic stress in CG14. Since CG14 has constitutively low CAD7, does it lodge or does it have sufficient lignin?
8. Line 437. Something seems to be missing here.
9. Fig. 6 discussion. Is there a specific key point where GSA1 acts or is it a more overall bulk flow?
10. Did the authors use technical and/or biological replicates for the qRT-PCR? It’s not clear in the methods.
Recommend editing for English grammar and usage, a few examples
Content(s) can be changes to level(s)
Dramatically to significantly
Weakens to reduces

Wendy Peer

Reviewer #3 (Remarks to the Author):

The manuscript by Dong et al. documented the process of identifying a QTL, named to GSA1, regulating rice grain size and abiotic stress tolerance. Firstly, the authors identified the QTL GSA1 for grain weight and grain size on chromosome 3 using a set of CSSLs. Then, they fine mapped the GSA1 locus to a 29.74-kb region and conducted overexpression and knockout of LOC_Os03g55040, a candidate gene for GSA1, for proving its function. The authors also confirmed that GSA1 catalyzes glucosylation of monoligonls and flavonoids for modulating flavonoid glycoside profiles and phenylpropanoid metabolism, thereby regulating rice grain size and abiotic stress tolerance. The authors provided lot of experiment evidences to support their findings. However, they could need to address and response the following questions before its possible publication.

1. In Fig.1f, how many recombinant plants was identified in this segregating population containing 5260 individuals? The authors should show genotypes and phenotypes (grain weight, grain length and grain width) of all recombinants in Fig. 1. In addition, the marker D3-125.1 is a co-segregation marker with the target QTL. So, the authors could not delimit the GSA1 locus to the 29.74-kb region between D3-125.1 and D3-125.48, and should reanalyze the fine-mapped region of GSA1.
2. Line130-135, the fine mapped interval of GSA1 comprises five putative genes. Especially, there are three UGT encoding genes in this region. Whether the variations in two other UGT genes affect grain size and stress tolerance, besides LOC_Os03g55040 (GSA1).
3. Line 138-141 and Line 367-369, the authors should provide experiment evidences for verifying the functional polymorphism (A349T and/or A246V). between WYJ and CG14.
4. Line 161-169, the authors performed overexpression transformation and gene knockout experiments for proving the function of LOC_Os03g55040. Why not conduct genomic complementary transformation?
5. Line 167-169 and Line 179-181, the authors speculated that the GSA1 from CG14 is a weakly functioning allele. So, overexpression of GSA1 from CG14 should show larger grains, instead of smaller grains.
6. Line 177-179, the expression level of GSA1 in NIL-GSA1 CG14 was higher than that in NIL-GSA1 WYJ. The authors should compare both the sequence and the activation of GSA1 promoter in CG14 and WYJ.
7. In Fig. 2b, the cellular evidence is weaker simply depending on the length and width of outer epidermal cells. Observation of inner epidermal cells, cross section and longitudinal cutting of spikelet hulls should be provided.
7. Line 216-236, the authors should provide more molecular evidences to support that GSA1 affects auxin biosynthesis, transport and signal transduction.
8. Line 374-381, could a QTL for abiotic stress tolerance be detected in the GSA1 locus using the segregating population (BC4F2)? Additionally, the authors should investigate the stress tolerance of all recombinants used in the fine-mapping of GSA1.
9. In Fig. 5 and Supplementary Fig. 7, the survival rate of WYJ under 42 °C for 26 hours was higher than that of NIL-GSA1 WYJ under 42 °C for 15 hours, and the survival rate of WYJ under 16% PEG for 14 d was lower than that of NIL-GSA1 WYJ under the same condition. Notably, the GSA1 overexpression lines was similar PEG tolerance to NIL-GSA1 CG14. The authors need check these data again.
10. Seedling development and spikelet development are two distinct stages. How does the GSA1 gene synergistically regulate abiotic stress tolerance at the seedling stage and spikelet development? In Fig. 6, the plants harboring GSA1 allele from WYJ under abiotic stress should reduce lignin biosynthesis, resulting in a smaller grain compared with the same plants under normal condition.
11. The authors should analyze nucleotide diversity and selection signatures in GSA1 in African and Asian rice.
12. In the Methods, the authors should describe the method and software used in QTL analysis.

13. In Supplementary Table 1, the authors should add the information of molecular marker. In addition, “Weight” should be “Additive effect”.

Point-by-point Response to Reviewers

Dear Reviewers,

We are very grateful for the three referees' comments on our manuscript. You provided valuable insights, critical comments and thoughtful suggestions, all of which were very helpful for revising and improving our manuscript, as well as providing important guidance for our future research. Based on your comments and suggestions, we performed further experiments (nine Supplementary Figures were added to the new manuscript) and made careful modifications to the original manuscript. We replaced the data for T_1-generation transgenic lines of *Pro35S:GSA1*^{WYJ}, *Pro35S:GSA1*^{CG14} and *KO-GSA1* with T_2-generation transgenic lines which provided more solid evidence to support our statements (new **Fig 1g-j** and new **Supplementary Fig. 7c-j**). We hope that the revised manuscript is more satisfactory. The main changes are highlighted in yellow in the revised version of the manuscript. Detailed descriptions of the revisions and responses to the reviewers' comments are provided below.

Reviewer #1 (Remarks to the Author):

The authors reported the cloning and characterization of a minor QTL that affected both grain size and abiotic stress tolerance in rice. The minor QTL, GSA1, encodes a UDP-glucosyltransferase (UGT83A1) that exhibits glucosyltransferase activities with broad substrate specificities towards flavonoids and monolignols. The authors further conclude that natural variation of GSA1 contribute to the variation of grain size in rice and GSA1 controls grain size through regulating auxin biosynthesis, transportation, and signaling. Although the results are overall interesting, with the concerns listed below, I am not sure the conclusions drawn by the authors are fully supported by the data presented.

Main points:

Comment-1. Line 37-141: in addition to the coding sequence analyzed, the authors need to compare the promoter region between the two parents to find the possible polymorphism underlying the differential expression of the two GSA1 alleles.

Response:

Thank you for this suggestion. We have compared the *GSA1* promoter region between the two parents and found 32 natural variations including nucleotide insertions, deletions and SNPs. Conserved motifs predicted by PlantCARE (i.e., TATA-box and CAAT-box) in the *GSA1*^{CG14} promoter region contain natural variations that may affect the binding of transcription factors to these motifs and the activation of the *GSA1* promoter (described in the revised manuscript line 149-153 and new **Supplementary Fig. 5**). It will be interesting to further study whether and to what extent these natural variations within the conserved motifs affect the expression of *GSA1* and which transcription factor bind to these motifs. Thank you again for your good comments.

Comment-2. Line 163-171: in addition to show that overexpression of GSA1WYJ in WYJ background affecting grain size, the authors really need to compliment the GSA1CG14 phenotype by introducing the genomic region of either GSA1WYJ or GSA1CG14 into CG14 background and to perform further experiments using these two lines.

Response:

Thank you for this constructive comment. We performed a genetic complementation test in which a DNA fragment from WYJ containing the putative promoter region, the entire ORF, and the 3' untranslated region of *GSA1* was introduced into NIL-*GSA1*^{CG14}. We have just obtained two complimentary lines, but one complimentary line only has two plants, which is not sufficient to perform statistical tests. Thus, we used the other complementation line to perform further analysis. NIL-*GSA1*^{CG14} transgenic line harboring the full-length *GSA1* transgene showed NIL-*GSA1*^{WYJ} phenotypes with respect to 1,000-grain weight, grain length and grain width (described in the revised manuscript line 195-200 and new **Supplementary Fig. 7k-n**). These data together with the data for overexpression lines and knock out lines indicate that we successfully cloned *GSA1* regulating grain size in rice.

Comment-3. Line 168-169: considering the GSA1CG14 is a weak allele in terms of glucosyltransferase activity, it is not reasonable the overexpressing lines resulted in

smaller grain size. The data from the two isogenic lines, the two complimentary lines as required above in main points #2 should also be included.

Response:

First of all, thank you for your insightful comment. The production of smaller grains by the *GSA1*^{CG14} overexpression lines might be the result of a dominant negative effect. Although *GSA1*^{CG14} is overexpressed, the glucosyltransferase activity of GSA1^{CG14} is still low. Therefore, we speculate that overexpression resulted in an excess of GSA1^{CG14}, which occupied the sugar acceptor, and decreased the amount of free substrate for GSA1^{WYJ} to bind and catalyse. As a result, glycosylation and metabolism were slowed down. Thus, the phenotype of the *GSA1*^{CG14} overexpression line is similar to that of *GSA1* knock-out lines. In complementation lines, GSA1^{WYJ}, which has higher catalytic efficiency towards sugar acceptors, was expressed at normal level resulting in normal levels of glycosylation and metabolism. Thus, the phenotype of the complementation lines is comparable to that of NIL-*GSA1*^{WYJ}. Thanks again for your insightful comments again.

Comment-4. Line 179-180: please give evidence for the suggestion.

Response:

Thank you for your comment. The expression level of *GSA1* is higher in NIL-*GSA1*^{CG14}, which exhibits smaller grains. This is surprising because *GSA1* is a positive regulator of grain size, and we have tried to explain this contradiction. Given that the glucosyltransferase activity of GSA1^{CG14} was lower than that of GSA1^{WYJ}, we speculated that a regulator of *GSA1* transcription may up-regulate the expression level of *GSA1* to produce more GSA1 protein in NIL-*GSA1*^{CG14}. In addition, conserved motifs containing natural variations were found in the *GSA1*^{CG14} promoter region; these variations also possibly affect the expression of *GSA1* (described in the revised manuscript line 149-153 and new **Supplementary Fig. 5**). Thanks again for your helpful comment.

Comment-5. Line 237-239: the authors need to give direct evidence that differential IAA biosynthesis, transportation, and signaling are really responsible for the variation of grain size in the two parents. I think at least data from complimentary experiment and genetic evidence need to be provided.

Response:

Thank you for raising these important points. Previous studies have revealed that IAA biosynthesis, transportation, and signaling result in the variation of grain size. *TSG1*, which encodes a tryptophan aminotransferase, plays a prominent role in local auxin biosynthesis in rice. Mutation of *TSG1* also affects auxin transport and downstream auxin signaling and result in smaller grains (Guo, *et al*., 2018). Overexpression of *BG1*, which is positive regulator of auxin response and is involved in auxin transport, leads to significantly increased grain size (Liu, *et al*., 2015). Loss of function of the auxin response factor *OsARF4* results in larger rice grains (Hu, *et al*., 2018). In our study, RNA-seq and qPCR assays revealed that the expression levels of genes (i.e., *TSG1*, *TAR1*, *TARL1*, *TARL2* and *BG1*) involved in auxin biosynthesis, transportation, and signaling were significantly decreased in NIL-*GSA1*^{CG14} (**Fig. 2h, i**), which exhibits smaller grains, compared with NIL-*GSA1*^{WYJ}. In addition, the endogenous OsPIN1 protein level in NIL-*GSA1*^{WYJ} was higher than that in NIL-*GSA1*^{CG14} (described in the revised manuscript line 282-283 and new **Supplementary Fig. 9q**). Thus, we suggest that the disruption of auxin biosynthesis, transport, and signaling in NIL-*GSA1*^{CG14} results in smaller rice grains (described in the revised manuscript line 286-288). We regret that time limitations prevented us from performing genetic tests in rice, which would require at least one year. However, the molecular evidence we provide in our study supports hypothesis that differences in auxin biosynthesis, signaling and transport underlie the differences in grain size between NIL-*GSA1*^{WYJ} and NIL-*GSA1*^{CG14}. We thank you for your kindly understanding.

References:

Guo, T. *et al*. TILLERING AND SMALL GRAIN 1 dominates the tryptophan aminotransferase family required for local auxin biosynthesis in rice. *J Integr Plant Biol* (2019).

Liu, L.C. *et al*. Activation of Big Grain1 significantly improves grain size by regulating auxin transport in rice. *PNAS* 112, 11102-11107 (2015).

Hu Z. *et al*. A Novel QTL qTGW3 Encodes the GSK3/SHAGGY-Like Kinase OsGSK5/OsSK41 that Interacts with OsARF4 to Negatively Regulate Grain Size and Weight in Rice. *Mol. Plant.* (2018). doi: 10.1016/j.molp.2018.03.005

Comment-6. Line 335-338: kinetics of the two GSA1 alleles need to be provided.

Response:

We sincerely thank the Reviewer for this constructive comment. We performed kinetic analysis of GSA1^{WYJ} and GSA1^{CG14} using kaempferol as a sugar acceptor. The *K*m values of GSA1^{WYJ} and GSA1^{CG14} were 5.107±1.086 mM and 8.557± 4.651 mM, respectively, indicating that GSA1^{WYJ} has a higher catalytic efficiency (described in the revised manuscript line 424-427 and new **Supplementary Fig. 11e)**.

We regret that we could not measure the *kcat* and *kcat*/*K*m ratio of GSA1. The high hydrophobicity of the GSA1 protein makes it difficult to express as a fusion protein in *E. coli*. The full-length cDNA sequence of *GSA1* was cloned into the pET28a vector, pET32a vector, pGEX4T-2 vector, pMAL-c5x vector and pCOLD-TF vector. Among them, only the fusion protein expressed from the recombinant plasmid pCOLD-*GSA1* was soluble in the supernatant; however, the fusion protein had no glycosyltransferase activity regardless of whether the trigger factor was cut by factor Xa or not. GSA1 protein with glycosyltransferase activity could not be expressed in an insect cell expression system either. Even so, the fact that the *K*m value of GSA1^{WYJ} is much lower than that of GSA1^{CG14}, provides further evidence of the weak glycosyltransferase activity of GSA1^{CG14}. We thank you for your understanding.

Comment-7. Line 379-380: the sensitivity of GSA1CG14 ox line need to be included. The data from the two isogenic lines, the two complimentary lines as required above in main points #2 should also be included.

Response:

Thank you for your thoughtful suggestions. As suggested, we investigated the sensitivity of the *GSA1*^{CG14} overexpression line to NaCl treatment. WYJ and *Pro35S:GSA1*^{CG14} were grown under normal conditions for 14 days and then transferred to 120 mM NaCl for 7 days and recovered for 7 days. *Pro35S:GSA1*^{CG14} seedlings exhibited decreased salt tolerance compared with WYJ seedlings (described in the revised manuscript line 443-445 and new **Supplementary Fig. 13g,h)**. The complementary transgenic line showed NIL-*GSA1*^{WYJ} phenotypes with respect to survival rate under NaCl treatment (described in the revised manuscript line 445-447 and **Supplementary Fig. 13k,l).**

Comment-8. Line 400-402: the authors need to provide data whether and to what extend GSA1WYJ and/or GSA1CG14 are induced by abiotic stresses.

Response:

Thank you for your constructive comments. We measured the expression level of *GSA1* before and after NaCl treatment for 5 days. The result confirmed that the expression levels of *GSA1* in NIL-*GSA1*^{WYJ} and NIL-*GSA1*^{CG14} were higher after NaCl treatment, and that the increase in NIL-*GSA1*^{WYJ} was larger than that in NIL-*GSA1*^{CG14} (described in the revised manuscript line 447-450 and new **Supplementary Fig. 13m)**, which indicates that *GSA1* is induced by abiotic stress and is involved in the abiotic stress response.

Comment-9. Line 427-428: the gene expression and metabolite profiling data showed by included for GSAl CRISPR lines and also the two complimentary lines as required above in main points #2.

Response:

Thank you for this constructive comment. As suggested, we first observed the survival rates of WYJ and *KO-GSA1* plants under NaCl treatment. We found that *KO-GSA1* seedlings were more sensitive to NaCl treatment compared with WYJ seedlings (described in the revised manuscript line 443-445 and new **Supplementary Fig. 13i,j)**.

Then we measured the expression levels of genes responsible for the central phenylpropanoid pathway, and biosynthesis of lignin and flavonoids before and after NaCl treatment in WYJ, *KO-GSA1* and *Pro35S:GSA1*^{WYJ}. We found that the central phenylpropanoid pathway failed to be induced under salt stress in *KO-GSA1* and that the lignin pathway was not down-regulated under abiotic stress in *KO-GSA1*. In addition, the results also indicated disruption of induction of the flavonoid biosynthesis pathway under abiotic stress in *KO-GSA1* (described in the revised manuscript line 470-471, 479-481 and 511-512, and new **Supplementary Fig. 15b)**. In contrast, genes involved in the central phenylpropanoid pathway were much more highly induced under salt stress in *Pro35S:GSA1*^{WYJ} than in WYJ; the decrease in expression of lignin pathway genes was larger in *Pro35S:GSA1*^{WYJ} than in WYJ; and the increase in expression of flavonoid biosynthesis pathway genes under abiotic stress was larger in *Pro35S:GSA1*^{WYJ} than in WYJ (described in the revised manuscript line 464-466, 477-478, 504-507 and 509-510, and new **Fig. 5g)**. The complementary transgenic line showed no significant difference in the expression levels of these genes compared with NIL-*GSA1*^{WYJ} (described in the revised manuscript line 481-483 and 512-514 and new **Supplementary Fig. 15c)**.

Given that the widely targeted metabolomics assay (WTMA) usually takes more than three months and we just harvested the T_1-generation of complementation lines in December, we did not have enough time to perform WTMA of the complementation lines and *KO-GSA1* before and after NaCl treatment. During the manuscript submission stage, we had started to perform WTMA of *Pro35S:GSA1*^{WYJ} and WYJ before and after NaCl treatment to further study the mechanism underlying metabolism flux redirection contributing to abiotic stress tolerance. We present these results as an alternative. The metabolomics data showed that the down-regulation of the lignin biosynthesis pathway was much more obvious in *Pro35S:GSA1*^{WYJ} compared with WYJ under salt stress (described in the revised manuscript line 532-535 and **Supplementary Fig. 17a)**. Moreover, the glycosidic flavonoids including apigenin-7-*O*-glucoside, chrysoeriol glucoside derivatives and anthocyanins accumulated at higher levels in *Pro35S:GSA1*^{WYJ} seedlings compared with WYJ

seedlings under salt stress (described in the revised manuscript line 545-549, and new **Supplementary Fig. 17b)**. The more effective metabolic flux redirection possibly leads to the enhanced abiotic stress tolerance in *Pro35S:GSA1*^{WYJ} seedlings. We hope these additional analyses will meet with your approval. Thanks to you for your excellent comment again.

Comment-10. The authors need to provide evidence that flavonoid aglycones suppress auxin pathway while glycosylated flavonoids do not.

Response:

Thank you for your insightful comment. The synthetic compound 1-N-naphthylphthalamic acid (NPA) has been used as an auxin transport inhibitor, and aglycone flavonoids that share structural similarity with NPA also function as endogenous inhibitors of auxin transport (Murphy *et al.*, 2000). Excess aglycone flavonoid in *tt7* and *tt3* which are required for flavonoid biosynthesis reduce the auxin transport (Peer *et. al*., 2004). Similarly, our study found that the aglycone flavonoid (kaempferol, naringenin and quercetin) were accumulated at higher levels in NIL-*GSA1*^{CG14} than that in NIL-*GSA1*^{WYJ}. In addition, the expression levels of genes related to auxin biosynthesis, transport and signaling were down-regulated in NIL-*GSA1*^{CG14}, and the endogenous OsPIN1 protein level in NIL-*GSA1*^{CG14} was lower than that in NIL-*GSA*^{WYJ} (described in the revised manuscript line 272-283 and **Fig. 2h,i and** new **Supplementary Fig. 9q**). Thus, we conclude that aglycone flavonoids suppress auxin pathway.

Flavonoids are synthesized at the end of phenylpropanoid pathway. Many phenylpropanoids are toxic and unstable molecules and so rarely accumulate in their aglycone form in plant cells. The mechanism of their toxicity is not clear. Glycosylation by UGT activity reduces their toxicity (Roy *et al.*, 2016). We hypothesized that one of flavonoid toxicity is the inhibition of the auxin transport, and glycosylation of flavonoids might alter their chemical structure, reduce their structural similarity with NPA, resulting in the reduced inhibition of auxin transport. It will be interesting to further investigate the direct effect of flavonoid glycosides on auxin

transport. We hope these will meet with your approval. Thank you for your constructive comment again.

Reference:

Murphy, A., Peer, W.A. & Taiz, L. Regulation of auxin transport by aminopeptidases and endogenous flavonoids. *Planta* **211**, 315-24 (2000).Peer, W.A. *et al.* Variation in expression and protein localization of the PIN family of auxin efflux facilitator proteins in flavonoid mutants with altered auxin transport in Arabidopsis thaliana. *Plant Cell* **16**, 1898-1911 (2004).

Le Roy, J., Huss, B., Creach, A., Hawkins, S. & Neutelings, G. Glycosylation Is a Major Regulator of Phenylpropanoid Availability and Biological Activity in Plants. *Frontiers in Plant Science* **7**(2016).

Minor points:

Comment-1. Line 110-111: the authors need to show the result of these QTL analyses in Supplementary figure.

Response:

Thank you for this suggestion. The QTL mapping results are shown in **Fig 1f**, and the results of QTL calculation are displayed in **Supplementary Table 1**. We have chosen to present the detailed mapping results (e.g., LOD value and percent variance explained) in table form to make them more accessible to the reader (**Supplementary Table 1)**.

Comment-2. Line 115-118: the authors need to add result of QTL mapping for number of grains per panicle to show whether the GSA1 locus can also be detected by this trait.

Response:

Thank you for your insightful comment. It will be interesting to determine whether the *GSA1* locus contributes to grain number per panicle. However, in the QTL mapping of study in which *GSA1* was identified as a QTL for grain size, which lasted

over four years, we didn't investigate the grain number per panicle of each recombinant line. In addition, most of the recombinant lines used in the QTL mapping study were harvested at least four years ago, thus now most of the seeds can hardly germinate. Therefore, we are unable to determine whether *GSA1* contributes to grain number per panicle. We hope these will meet with your approval. Thank you again for your constructive comment.

Comment-3. Line 119-120: the authors showed that GSA1 didn't affect grain yield, however this is not consistent with what it is claimed in the Title and the Abstract.

Response:

Thank you for pointing this out. According to the Reviewer's comment, we revised the Title and Abstract to make them more accurate (in the revised manuscript line 2 and 43).

Comment-4. Line 231-236: are TSG1 and their homologs somehow related to auxin pathway?

Response:

Thank you for raising this good question. Previous studies have revealed that *TSG1*, which encodes a tryptophan aminotransferase, dominates the tryptophan aminotransferase family and is required for local auxin biosynthesis and thus affects auxin signaling and transport. The *tsg1* mutant showed hypersensitivity to indole-3-acetic acid. TSG1 and its homolog OsTAR1, but not the homologs OsTARL1 and OsTARL2, displayed marked aminotransferase activity, which is responsible for auxin biosynthesis (Guo, *et al*., 2019).

References:

Guo, T. *et al*. TILLERING AND SMALL GRAIN 1 dominates the tryptophan aminotransferase family required for local auxin biosynthesis in rice. *J Integr Plant Biol* (2019).

Comment-5. Lin 270-212: what about the flavonoid content in the leaves of the two isogenic lines and also in the complimentary lines as indicated in main points #2.

Response:

Thank you for your insightful comment. As we have mentioned in the response to Comment-9 (under Main points), because WTMA usually takes more than three months to complete and we just harvested the T_1-generation of complementation lines in December, we did not have enough time to perform WTMA of the leaves of the two isogenic lines and the complementation lines. We hope that the reviewer will understand this limitation. Thank you for your constructive comment once more.

Comment-6. Line 245-247: the authors need to include the phylogenetic analysis of GSA1 with its homologs in rice and other plant species.

Response:

Thank you for the thoughtful comment. As suggested, we performed the phylogenetic of GSA1 and its homologs in *Gramineae*, *Brassicaceae Burnett* and *Leguminosae*. We found that the GSA1 sequences of monocotyledons and dicotyledons were separated into two branches, which suggests that *GSA1* had existed before monocotyledons and dicotyledons divided during plant evolution, and that *GSA1* is a conserved gene with a fundamental function (described in the revised manuscript line 143-148 and new **Supplementary Fig. 4c**).

Reviewer #2 (Remarks to the Author):

I really enjoyed reading this. The authors have conducted a thorough analysis of GSA1 and the presentation is very clear. The authors present a study where they mapped a UGT-glucosyl transferase, UGT83A1/GSA1, in rice. This is a minor QTL controlling grain that was not previously mapped. The authors found that GSA1 not only glycosylated many flavonoid species, but also documented two other points: 1) grain size changes were due to cell length and cell number, and 2) overexpression of GSA1 activity also increased abiotic stress tolerance. The authors conclude that GSA1 activity results in metabolic flux changes in the phenyl propanoid pathway and propose that the mechanism is via a feedback loop. However, the author conclude in several places that GSA1 also directly controls auxin biosynthesis, but how GSA1 directly acts on auxin biosynthesis is not clear.
There are a few minor comments about some of the interpretations and presentation of the conclusions that need to be addressed.

Comment-1.The authors have presented one interpretation of the data, and a in a few cases, the authors may consider adding an alternate hypothesis or alternate interpretation of their data. I encouraged the authors to expand a bit more on the other possibilities.

Response:

We sincerely thank the Reviewer for the constructive comments. We have paid more attention to our interpretations and hypotheses and provided more possible explanations for our findings. For example, we have expanded our interpretation of the finding that *GSA1* has a higher expression level in NIL-$GSA1^{CG14}$, which exhibits smaller grains. This is a surprising finding because *GSA1* is a positive regulator of grain size, and we have tried to provide more explanation for this contradiction. One hypothesis we came up with is that because of the decreased glucosyltransferase activity of $\mathrm{GSA1}^{\mathrm{CG14}}$, a regulator of *GSA1* up-regulates the expression level of *GSA1* to produce more GSA1 protein. Another hypothesis we put forward is that natural variations in the conserved motif of $GSA1^{CG14}$ promoter region may affect transcription factor binding and activation of *GSA1* promoter (described in the revised manuscript line 149-153 and new **Supplementary Fig. 5**).

Comment-2. One example is alluded to above regarding GSA1 control of auxin biosynthesis. Since does not encode an auxin biosynthetic enzyme and or a transcription factor, direct control is unlikely. The effect may be indirect. The gene

expression of the auxin responsive genes make sense given the auxin levels. The auxin levels and expression of auxin metabolic genes and transporters appear match in the two cultivars, respectively. Perhaps gene expression is constitutively low in CG14 overall? This appears to be the case generally in the data presented. However, there does appear to be a case where CG14 has increased expression in YP compared to WYJ (Fig. 3a). Can this helpful in interpreting your data?

Response:

Thank you for the very useful comment and insightful interpretation of our data. This insight has led us to conclude that *GSA1*, which encodes a UDP-glucosyltransferase instead of an auxin biosynthetic enzyme or a transcription factor, is indirectly involved in auxin biosynthesis, signaling and transport indirectly (described in the revised manuscript line 284, 287 and 592). Further evidence supporting this conclusion was provided by measurement of auxin levels and expression of auxin metabolic genes and transporters genes. In addition, the endogenous OsPIN1 protein level in NIL-$GSA1^{WYJ}$ was higher than that in NIL-$GSA1^{CG14}$ (described in the revised manuscript line 282-283 and new **Supplementary Fig. 9q**). Thus, we speculate that $GSA1^{CG14}$ indirectly interferes with auxin biosynthesis, signaling and transport. The higher *GSA1* expression level in NIL-$GSA1^{CG14}$ might be caused by the weak activity of $\mathrm{GSA1}^{\mathrm{CG14}}$ through a feed-back loop or by natural variation in the conserved domains of *GSA1* promoter, as discussed in the response to Comment-1. The weak activity of $\mathrm{GSA1}^{\mathrm{CG14}}$ also leads to accumulation of flavonoids, which interferes with auxin transport and the subsequent auxin biosynthesis and signaling. As a result, most genes related to auxin transport, biosynthesis and signaling were down-regulated in NIL-$GSA1^{CG14}$.

Comment-3. Grain cell number (cell division) is also regulated by cytokinin, so this is also a possible mechanism in addition to the role of auxin in cell elongation. Lines 234-237, Lines 495, etc, make a definitive statement, that is extrapolating the data too far, in my opinion. Can the authors modify these sentence? The characterization in lines 520-521 more in keeping with what the date show. Also, Fig 6 does not show GSA1 acting directly on auxin, which is consistent with what the authors state in lines 520-521 and to my thinking.

Response:

Thank you for your insightful comments.

1) It is known that both cytokinin and auxin are responsible for regulating cell division (Moubayidin, *et al*., 2009). However, no significant difference was observed in the expression level of genes related to cytokinin biosynthesis (*IPT7*, *IPT9* and *IPT10*) or signalling (*RR1*, *RR2*, *RR3*, *RR4*, *RR5*, *RR6* and *RR7*) between young panicles of NIL-*GSA1*^{WYJ} and NIL-*GSA1*^{CG14} (Please see figure below). Therefore, we have no solid evidence to conclude that the difference in cell number between NIL-*GSA1*^{WYJ} and NIL-*GSA1*^{CG14} was caused by differences in the cytokinin pathway.

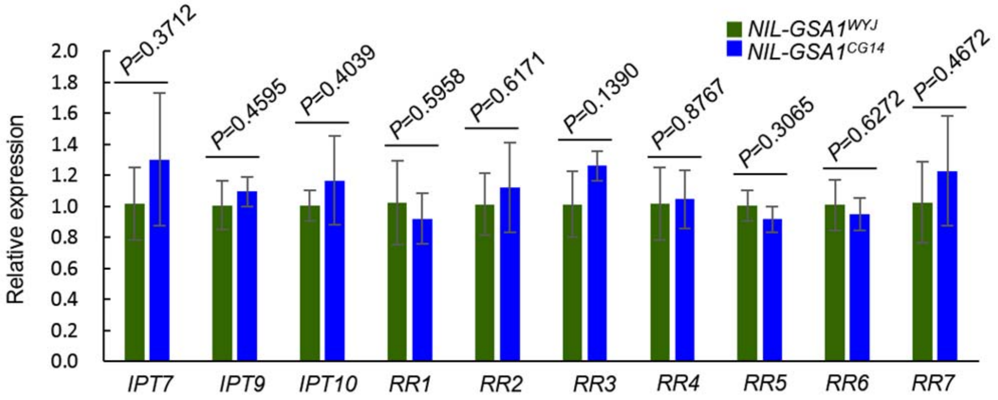

2) As the reviewer suggested, we thought more about our statements about regarding *GSA1* and the auxin pathway and realized that some statements were overstatement. We have revised these sentences to make our conclusion more accurate (the revised manuscript line 284, 287 and 592). Thank you again for your helpful comments.

References:

Moubayidin, L., Di Mambro, R. & Sabatini, S. Cytokinin-auxin crosstalk. *Trends Plant Sci* **14**, 557-62 (2009).

Comment-4. Since other UGTs are in the QTL and a gene of unknown function, and there is more scopolin, heavily glucosylated apigenen, etc., in CG14, is it possible that CG14 might have some other compensatory mechanism for a partially active GSA1 that increases these glucosylated compounds? Are there any differences/natural variation between GC14 and WJY besides GSA1 known? Is it possible that has overall lower gene expression than in WYJ?

Response:

We sincerely thank the reviewer for these constructive comments. There are three UGT genes within the target region, namely *LOC_Os03g55030*, *LOC_Os03g55040*

(*GSA1*) and *LOC_Os03g55050*. The amino acid substitutions between LOC_Os03g55030 proteins of the two NILs are F20L, Y137S and I220V. The substitutions in LOC_Os03g55050 are P7L, D90A, I277V and F388C. None of these natural variations occur in the PSPG-box. Since the weak glycosyltransferase activity of GSA1^{CG14} is caused by the A349T substitution within the PSPG-box (described in the revised manuscript line 135-141 and 417-424 and new **Supplementary Fig. 4a** and new **Supplementary Fig. 11a-d**), the other UGTs possibly replace the glycosyltransferase activity of GSA1 through a compensatory mechanism, even though, their substrates are not necessarily the same as those of GSA1. As a result, some flavonoid glycosides such as scopolin possibly accumulate in NIL-*GSA1*^{CG14}. We thank the reviewer for reminding us of the possible compensatory mechanism of the three UGTs, which led us to further study this issue. We found that the expression levels of *LOC_Os03g55030* and *LOC_Os03g55050* in NIL-*GSA1*^{CG14} panicles were comparable to those in NIL-*GSA1*^{WYJ} (described in the revised manuscript line 204-206 and new **Supplementary Fig. 8k)**. Moreover, knock-out lines of *LOC_Os03g55030* and *LOC_Os03g55050* obtained using the CRISPR/Cas9 system exhibited no significant difference compared with WYJ with respect to grain size and abiotic stress tolerance (described in the revised manuscript line 200-204 and 452-457, and new **Supplementary Fig. 8a-j** and new **Supplementary Fig. 14a-f)**. Thus, we conclude that *LOC_Os03g55040* is the causal gene for *GSA1* locus. Thank you again for your insightful comments.

Comment-5. Fig. S6d, e. For the image it appears that the empty vector (negative control) is the same as CG14? Was the baseline (negative control) subtracted from all of the data? That may affect the presentation of these data if CG14 is equivalent to negative control. Can that authors add a sentence that these are in vitro assays, and that some of the reactions may not necessarily occur in in vivo if the enzyme and substrates are not in the same cell type or compartment?

Response:

We thank the reviewer for this good comments. In fact, the dotted line on the left (the middle peak in new **Supplementary Fig. 10d**) indicates the peak of glycosylated

coniferyl alcohol (not coniferin) produced by GSA1^{WYJ} and GSA1^{CG14}, and the dotted line on right indicates the sugar acceptor, namely coniferyl alcohol. GSA1^{CG14} produced significantly less glycosylated coniferyl alcohol than GSA1^{WYJ}, while the empty vector (negative control) did not glycosylated coniferyl alcohol. We hope that we made our interpretation clear enough. Thank you again for your comments.

Comment-6. Lines 392-394. While I believe that you can make this statement for the phenylpropanoid pathway because substrate shunting and the feedback mechanism is largely known, I do not agree that this same logic can be applied to auxin biosynthesis when it is applied in other places in this work.

Response:

Thank you for your constructive comments. We have looked into this issue some more. Indeed, previous studies have revealed that UGT genes are responsible for metabolic redirection in plants. The *ugt78d1 ugt78d2* double mutant, which has compromised 3-O-glycosylation of flavonol, exhibits severely altered flavonol glycoside profiles, reduced total flavonol content and redirection of metabolic flux within the flavonol biosynthesis pathway (Yin *et al*, 2012). Over-expressing *UTG72E3* leads to the redirection of metabolic flux from the phenylpropanoid-dependent sinapate esters pathway to the lignin pathway (Lanot *et al*, 2008). In our study, we found that *GSA1* plays a key role in metabolic redirection under abiotic stress. However, we do not have enough evidence to conclude that *GSA1* is directly responsible for auxin biosynthesis. Thus we revised our statements about *GSA1* and auxin biosynthesis to make them consistent with the available evidence (described in the revised manuscript lines 284, 287 and 592). Thank you again for your excellent comments.

References:

Yin, R. *et al.* Feedback inhibition of the general phenylpropanoid and flavonol biosynthetic pathways upon a compromised flavonol-3-O-glycosylation. *J Exp Bot* **63**, 2465-78 (2012)

Lanot, A., Hodge, D., Lim, E.K., Vaistij, F.E. & Bowles, D.J. Redirection of flux through the phenylpropanoid pathway by increased glucosylation of soluble intermediates. *Planta* **228**, 609-616 (2008).

Comment-7. Lines 399-400. I think that concluding that GSA1 is the key regulator of lignin biosynthesis under abiotic stress is also an overstatement, especially as CAD7 expression is not affected in bi biotic stress in CG14. Since CG14 has constitutively low CAD7, does it lodge or does it have sufficient lignin?

Response:

We thank the reviewer for the useful comments. We have looked into this issue.

1) Actually, there was no difference in the expression level of *CAD7* after NaCl treatment not only in NIL-*GSA1*^{CG14} but also in the *KO-GSA1* lines (described in the revised manuscript line 479-481 and new **Supplementary Fig. 15a,b**), implying that the down-regulation of lignin biosynthesis after abiotic stress treatment is disrupted in NIL-*GSA1*^{CG14} and *KO-GSA1*. However, the decrease in *CAD7* expression level was larger in *Pro35S:GSA1* than in WYJ after NaCl treatment (described in the revised manuscript line 477-479 and new **Fig. 5g**), suggesting that there was more down regulation of lignin biosynthesis under abiotic stress in *Pro35S:GSA1*. These results suggested that *GSA1* takes part in the down regulation of lignin biosynthesis under abiotic stress. We have revised our conclusion to make it consistent with the evidence (line 484 in the revised manuscript).

2) As the reviewer pointed out, since *CAD7* is lowly expressed in NIL-*GSA1*^{CG14}, the lignin content of spikelet hulls and caryopses were lower in NIL-*GSA1*^{CG14}than in NIL-*GSA1*^{WYJ} (described in the revised manuscript 329-331 and **Fig. 3c**). However, the plant height, number of effective panicles, panicle length and grain yield per plant values for NIL-*GSA1*^{CG14} were comparable to those for NIL-*GSA1*^{WYJ} (described in the revised manuscript 118-120 and new **Supplementary Fig. 1e-j**), suggesting that NIL-*GSA1*^{CG14} has sufficient lignin to meet the requirements for rice plant development and reproduction. Special thanks to you for your helpful comments once more.

Comment-8. Line 437. Something seems to be missing here.

Response:

Thank you very much for pointing this out. We are sorry for our carelessness. In this line, we meant to say that the levels of monolignols (p-coumaryl alcohol, sinapyl alcohol and coniferyl alcohol, which accept sugars from GSA1) were largely reduced (line 527 in the revised manuscript).

Comment-9. Fig. 6 discussion. Is there a specific key point where GSA1 acts or is it a more overall bulk flow?

Response:

Thank you for raising this question. *GSA1* is responsible for the redirection of metabolic flux underlying abiotic stress tolerance. However, how *GSA1* senses the stress signal and the upstream regulator of *GSA1* are largely unknown. The mechanism underlying the regulation of metabolism in response to abiotic stress is also unclear. We thank the reviewer for pointing out these intriguing issues, which arouse our interest to further study it.

Comment-10. Did the authors use technical and/or biological replicates for the qRT-PCR? It's not clear in the methods.
Recommend editing for English grammar and usage, a few examples
Content(s) can be changes to level(s)
Dramatically to significantly
Weakens to reduces
Wendy Peer

Response:

Thank you for raising these concerns.

1) We are sorry for our carelessness. The number of biological replicates were shown in the figure legends, and we forgot to add it to the methods. We have included them in the revised manuscript (described in the line 737-738).

2) We have examined the manuscript carefully and corrected the grammatical mistakes (the lines 266, 270, 273, 299, 302, 307, 321, 325, 349, 464, 497, 523, 537, 538, 609, 612, 654, 775, 1145, 1151, 1205 and 1464 in the revised manuscript). Thank you again for your excellent comments.

Reviewer #3 (Remarks to the Author):

The manuscript by Dong et al. documented the process of identifying a QTL, named to GSA1, regulating rice grain size and abiotic stress tolerance. Firstly, the authors identified the QTL GSA1 for grain weight and grain size on chromosome 3 using a set of CSSLs. Then, they fine mapped the GSA1 locus to a 29.74-kb region and conducted overexpression and knockout of LOC_Os03g55040, a candidate gene for GSA1, for proving its function. The authors also confirmed that GSA1 catalyzes glucosylation of monoligonls and flavonoids for modulating flavonoid glycoside profiles and phenylpropanoid metabolism, thereby regulating rice grain size and abiotic stress tolerance. The authors provided lot of experiment evidences to support their findings. However, they could need to address and response the following questions before its possible publication.

Comment-1. In Fig.1f, how many recombinant plants was identified in this segregating population containing 5260 individuals? The authors should show genotypes and phenotypes (grain weight, grain length and grain width) of all recombinants in Fig. 1. In addition, the marker D3-125.1 is a co-segregation marker with the target QTL. So, the authors could not delimit the GSA1 locus to the 29.74-kb region between D3-125.1 and D3-125.48, and should reanalyze the fine-mapped region of GSA1.

Response:

Thanks for your constructive comments sincerely. Actually, the numbers of recombinant individuals are shown between the marker positions in **Fig. 1f**. As suggested, the grain length and grain width data are now shown in new **Supplementary Fig. 3**. We regret that we did not accurately interpret the results of fine mapping of the *GSA1* locus. Indeed, we delimited the *GSA1* locus to a 29.47-kb region between D3-125.37 (very close to D3-125.1 on the right of D3-125.37) and D3-125.49 (very close to D3-125.48 on the left of D3-125.49). Then we identified a recombinant individual in which the D3-125.1 to D3-125.48 region was heterozygous and the left border marker D3-125.37 and the right border marker D3-125.49 were homozygous for WYJ (this recombinant individual was obtained by screening two generations of progeny) to develop the BC_4F_2 generation and conduct analysis of the *GSA1* locus. We have revised **Fig. 1f** (added: Recombinant lines R2, R3 and R4 originated from recombinant line C2; recombinant line R1 originated from

recombinant line R2 in the legends) and **Supplementary Table 1** accordingly. Thank you again for your helpful comments.

Comment-2. Line130-135, the fine mapped interval of GSA1 comprises five putative genes. Especially, there are three UGT encoding genes in this region. Whether the variations in two other UGT genes affect grain size and stress tolerance, besides LOC_Os03g55040 (GSA1).

Response:

Thank you for the thoughtful comment. There are three UGT genes within the target region, namely *LOC_Os03g55030*, *LOC_Os03g55040* (*GSA1*) and *LOC_Os03g55050*. The amino acid variations between the two alleles of *LOC_Os03g55030* are F20L, Y137S and I220V. Those between the two alleles of *LOC_Os03g55050* are P7L, D90A, I277V and F388C. None of these natural variations occur in the PSPG-box. Since the weak glycosyltransferase activity of $GSA1^{CG14}$ is caused by the A349T substitution within the PSPG-box (described in the revised manuscript line 135-141 and 417-424 and new **Supplementary Fig. 4a** and new **Supplementary Fig. 11a-d**), the other UGTs possibly replace the glycosyltransferase activity of GSA1 through a compensatory mechanism. However, the expression levels of *LOC_Os03g55030* and *LOC_Os03g55050* in NIL-$GSA1^{CG14}$ panicles were comparable to those in NIL-$GSA1^{WYJ}$ (described in the revised manuscript line 204-206 and new **Supplementary Fig. 8k)**. Moreover, the knock-out lines of *LOC_Os03g55030* and *LOC_Os03g55050* generated using the CRISPR/Cas9 system exhibited no significant difference compared with WYJ with respect to grain size and abiotic stress tolerance (described in the revised manuscript line 200-204 and 452-457, and new **Supplementary Fig. 8a-j** and new **Supplementary Fig. 14a-f)**. Thank you again for your insightful comment.

Comment-3. Line 138-141 and Line 367-369, the authors should provide experiment evidences for verifying the functional polymorphism (A349T and/or A246V). between WYJ and CG14.

Response:

Thank you for your helpful comment sincerely. To identify the amino acid change underlying the differential activity of $GSA1^{WYJ}$ and $GSA1^{CG14}$, we heterologously expressed two versions of GSA1, $GSA1^{A246V}$ and $GSA1^{A349T}$, in *Escherichia coli*. HPLC analysis revealed that the glucosyltransferase activity of $GSA1^{A246V}$ towards kaempferol and sinapyl alcohol was comparable to that of $GSA1^{WYJ}$, and the glucosyltransferase activity of $GSA1^{A349T}$ was comparable to that of $GSA1^{CG14}$ (described in the revised manuscript line 417-424 and new **Supplementary Fig. 11a-d**). These results imply that the alanine at position 349 within the conserved PSPG box domain is vital for the glucosyltransferase activity of GSA1. Thank you again for your excellent comment.

Comment-4. Line 161-169, the authors performed overexpression transformation and gene knockout experiments for proving the function of LOC_Os03g55040. Why not conduct genomic complementary transformation?

Response:

Thank you for your helpful suggestion. We have performed a genetic complementation test in which a DNA fragment from WYJ containing the putative promoter region, the entire ORF, and the 3' untranslated region of *GSA1* was introduced into NIL-$GSA1^{CG14}$. We have just obtained two complementary lines, but one complementary line only has two plants, which is not sufficient to perform statistical tests. Thus, we used the other complementary line to perform further analysis. NIL-$GSA1^{CG14}$ transgenic lines harboring the full-length *GSA1* transgene showed NIL-$GSA1^{WYJ}$ phenotypes with respect to 1,000-grain weight, grain length and grain width (described in the revised manuscript line 195-200 and new **Supplementary Fig. 7k-n**). These data together with the data for overexpression lines and knock-out lines indicated that we have successfully cloned *GSA1*. Thank you for your helpful comment again.

Comment-5. Line 167-169 and Line 179-181, the authors speculated that the GSA1 from CG14 is a weakly functioning allele. So, overexpression of GSA1 from CG14 should show larger grains, instead of smaller grains.

Response:

Thank you for your insightful comment. The smaller grains produced by *GSA1*^{CG14} overexpression lines might be the result of a dominant negative effect. Even though *GSA1*^{CG14} was overexpressed, glucosyltransferase activity of GSA1^{CG14} is still low. Therefore, we speculate that overexpression resulted in an excess of GSA1^{CG14}, which occupied the sugar acceptor, and decreased the amount of free substrate for GSA1^{WYJ} to bind and catalyse. As a result, glycosylation and metabolism were slowed down. Thus, the phenotype of the *GSA1*^{CG14} overexpression line is similar to that of *GSA1* knock-out lines. We thank you for your understanding.

Comment-6. Line 177-179, the expression level of GSA1 in NIL-GSA1 CG14 was higher than that in NIL-GSA1 WYJ. The authors should compare both the sequence and the activation of GSA1 promoter in CG14 and WYJ.

Response:

Thank you for your helpful suggestion. We analysed the promoter region between the two parents and found that 32 natural variations including nucleotide insertions, deletions and SNPs. Variations in conserved motifs (i.e., TATA-box and CAAT-box) in *GSA1*^{CG14} promoter region may affect the binding of transcription factors to these motifs and the activation of the *GSA1* promoter (described in the revised manuscript line 149-153 and new **Supplementary Fig. 5**). It will be interesting to further study whether and to what extent these natural variations within the conserved motifs affect the expression of *GSA1* and which transcription factor binds to these motifs. Thank you again for your insightful comment.

Comment-7. In Fig. 2b, the cellular evidence is weaker simply depending on the length and width of outer epidermal cells. Observation of inner epidermal cells, cross section and longitudinal cutting of spikelet hulls should be provided.

Response:

Thanks for the helpful suggestion. As suggested, we compared cross sections of the central parts of the spikelet hulls in NIL-*GSA1*^{WYJ} and NIL-*GSA1*^{CG14} at the booting stage. Our observations revealed that there were significantly fewer parenchyma cells

in NIL-*GSA1*^{CG14} compared with NIL-*GSA1*^{WYJ} (described in the revised manuscript line 251-254 and new **Supplementary Fig. 9k, l**). Observation of inner epidermal cells revealed that the average length and width of cells in NIL-*GSA1*^{CG14} spikelet hulls was significantly decreased compared with NIL-*GSA1*^{WYJ} (described in the revised manuscript line 254-257 and new **Supplementary Fig. 9m-o**). In addition, we tried our best to perform longitudinal sectioning of spikelet hulls, but we failed to obtain the unbroken sections of spikelet halls located at the same position. Nevertheless, the cross sectioning of spikelet hulls and the observation of inner epidermal cells and outer epidermal cells have provided solid evidence that *GSA1* is a QTL that finely regulates spikelet development by controlling cell proliferation and cell expansion. Thank you again for your insightful comments.

Comment-7. Line 216-236, the authors should provide more molecular evidences to support that GSA1 affects auxin biosynthesis, transport and signal transduction.

Response:

Thank you for the constructive comment. As suggested, we performed immunoblotting assays to investigate endogenous levels of OsPIN1 protein in NIL-*GSA1*^{WYJ} and NIL-*GSA1*^{CG14}. We found that the endogenous level of OsPIN1 protein was clearly decreased in young panicles of NIL-*GSA1*^{CG14} compared with that of NIL-*GSA1*^{WYJ} (described in the revised manuscript line 282-283 and new **Supplementary Fig. 9q**). Unfortunately, we do not have specific antibody for assaying the levels of proteins related to auxin biosynthesis and signal transduction, and it is difficult to make a usable antibody for these proteins in a short time. Nevertheless, the immunoblotting assays of endogenous OsPIN1 protein levels together with RNA-seq and qPCR assays have provided solid evidence for the hypothesis that *GSA1* is indirectly involved in auxin biosynthesis, transport and signal transduction during multiple stages of panicle and spikelet development in rice. Thank you again for your helpful comment.

Comment-8. Line 374-381, could a QTL for abiotic stress tolerance be detected in the GSA1 locus using the segregating population (BC4F2)? Additionally, the authors

should investigate the stress tolerance of all recombinants used in the fine-mapping of GSA1.

Response:

Thank you for the insightful comments. As suggested, we tried to use the BC_4F_2 and the recombinant lines to perform abiotic stress tolerance assays. Unfortunately, the seeds of these lines were no longer able to germinate because they have been stored for at least four years after harvest. However, we have investigated the contribution of the *GSA1* locus to abiotic stress tolerance using NIL-*GSA1*^{WYJ} and NIL-*GSA1*^{CG14}. Thus, we hope for the Reviewer's kindly understanding and approval. Thank you again for your excellent comments again.

Comment-9. In Fig. 5 and Supplementary Fig. 9, the survival rate of WYJ under 42 °C for 26 hours was higher than that of NIL-GSA1 WYJ under 42 °C for 15 hours, and the survival rate of WYJ under 16% PEG for 14 d was lower than that of NIL-GSA1 WYJ under the same condition. Notably, the GSA1 overexpression lines was similar PEG tolerance to NIL-GSA1 CG14. The authors need check these data again.

Response:

Thank you for pointing this out. Indeed, we have tried our best to ensure that both the control lines and experimental lines were grown and were treated under the same conditions. Therefore, it makes sense to compare of the survival rate between the control lines and experimental lines grown in the same batch. However, since the plants were placed in different positions in light growth incubators, the growth vigor of rice seedlings of the same genotype was not identical between different parallel experiments (new **Supplementary Fig. 12a-c** and new **Supplementary Fig. 13 a** (left), **c** (left), **e** (left)**, g** (left), **i** (left) and **k** (upper)). As a result, the survival rate of rice seedlings with the same genotype from different parallel experiments might not be exactly the same, and it is not appropriate to compare the survival rate of the seedlings with different genotype from different parallel experiments. We hope that the reviewer will understand this and give an approval. Thank you again for your insightful comments.

Comment-10. Seedling development and spikelet development are two distinct stages. How does the GSA1 gene synergistically regulate abiotic stress tolerance at the seedling stage and spikelet development? In Fig. 6, the plants harboring GSA1 allele from WYJ under abiotic stress should reduce lignin biosynthesis, resulting in a smaller grain compared with the same plants under normal condition.

Response:

Thank you for your insightful comments. Indeed, the expression level of *GSA1* was relatively high in leaves and spikelets (**Fig 2a**), suggesting that *GSA1* acts as glucosyltransferase during both seedling and spikelet development. When subjected to abiotic stress, survival is of top priority for plant and energy and metabolic flux will be coordinated to respond to abiotic stress. *GSA1* takes part in the redirection of metabolic flux, leading to the production of more flavonoid glycosides to scavenge ROS and protect the plant from damage. Thus, allowing plants to survive the seedling stage and continue growing to the reproductive stage; the spikelet then obtains energy and metabolites from the surviving plant tissues to generate caryopses and seed. Similar to its role at the seedling stage, *GSA1* may be involved in the induction of flavonoid biosynthesis and abiotic stress tolerance during spikelet development. However, we regret that we did not have enough time to investigate the abiotic stress tolerance during rice reproductive stage, which will takes more than half a year.

In addition, lignin is required during both seedling growth and spikelet development, and the biosynthesis of lignin is regulated by *GSA1*. It is quite reasonable to speculate that grain size should be reduced under abiotic stress compared with normal conditions due to the down-regulation of lignin biosynthesis. We agree with the reviewer's suggestion and have replaced the rice grain diagram with smaller one (**Fig 6b**, the upper part).

Comment-11 The authors should analyze nucleotide diversity and selection signatures in GSA1 in African and Asian rice.

Response:

We sincerely thank the reviewer for this constructive suggestion, and we have analysed the nucleotide diversity and selection signatures in *GSA1* in African and Asian rice. We examined the nucleotide diversity in a ~3.3-kb genomic region containing the ~1-kb promoter and the ~1.6-kb entire ORF of *GSA1* from the Rice3K data (Mansueto, *et al*., 2016), 446 *O. rufipogon* accessions (Huang, *et al*., 2012), 20 *O. glaberrima* varieties and 94 *O. barthii* accessions (Wang, *et al*., 2014). A sliding-window analysis of the nucleotide diversity in the 3.3-kb region showed that the π value in *O. glaberrima* varieties was much lower than that in *O. barthii* (described in the revised manuscript line 155-162 and new **Supplementary Fig. 6a**). The π value in *O. japonica* varieties was much lower than that in *O. rufipogon* (described in the revised manuscript line 162-163 and new **Supplementary Fig. 6b**). The π value in *O. indica* varieties was much higher than that in *O. rufipogon* (described in the revised manuscript line 163-166 and new **Supplementary Fig. 6c**), implying that more natural variations occurred during the domestication of *O. indica* varieties from *O. rufipogon*. These results demonstrated that *GSA1* has been directionally selected in *O. glaberrima* varieties and *O. japonica* varieties during the domestication of African and Asian rice (described in the revised manuscript line 166-168).

References:

Mansueto, L. *et al.* Rice SNP-seek database update: new SNPs, indels, and queries. *Nucleic acids research* **45**, D1075-D1081 (2016).

Huang, X. *et al.,* A map of rice genome variation reveals the origin of cultivated rice *Nature*. 2012 Oct 25;490(7421):497-501

Wang, M. *et al*., The genome sequence of African rice (*Oryza glaberrima*) and evidence for independent domestication. *Nat Genet*. 2014 Sep;46(9):982-8

Comment-12. In the Methods, the authors should describe the method and software used in QTL analysis.

Response:

Thank you for pointing this out. As suggested, we described the methods and software used in QTL analysis in Lines 727-729.

Comment-13. In Supplementary Table 1, the authors should add the information of molecular marker. In addition, “Weight” should be “Additive effect”.

Response:

Thank you for these suggestions. We added the molecular marker information to Supplementary Table 1. In addition, we have changed the “Weight” into “Additive effect”. We sincerely thank you for your insightful comments.

Reviewers' comments:

Reviewer #1 (Remarks to the Author):

I think the authors have made substantial improvement to the work and the manuscript. There are still two points need to be solved.

1. For comment-5 (on page 4 of Response to Referees Letter): the author need to perform extra measurement of IAA and the transcription of the related genes using the complimentary lines.
2. For comment-6 (on page 5 of Response to Referees Letter): the Kms measured by the authors are several orders different to the published flavonoid UGTs. And it seems that the two UGTs show very similar Kms from the data provided by the authors, which is not in line with the conclusion drawn by the authors. I think the authors need to redo this experiment to give more reliable data.

Reviewer #2 (Remarks to the Author):

The authors have addressed my questions and concerns in the revision.

In the revision, can that author include information in the methods regarding the alignments, phylogenetic analyses, and tree building (Figure S4)?

Wendy Peer

Reviewer #3 (Remarks to the Author):

The authors conducted additional experiments and revised the relevant statements appropriately. I believe this manuscript provided valuable scientific and useful agricultural information.
Minor points:
1. GSA1 explained approximately 14% phenotype variation of grain size (Supplementary Table 1). So, this QTL did not a minor QTL.
2. The authors obtained three independent recombinant plants of R3 type and two recombinant plants of R2 type. I suggest the authors to provide the phenotype data of each independent recombinant plant in Figure 1f and Supplementary Figure 3.
3. In main text and Supplementary Figure 6, "O. japonica" should be "japonica" or "O. sativa ssp. japonica", and "O. indica" should be "indica" or "O. sativa ssp. indica". In addition, in Supplementary Fig 6 b and c, "O. rufipogen" should be "O. rufipogon".
4. Recommend editing for English grammar and usage to improve the readability of this paper.

Point-by-point Response to Reviewers

Dear Reviewers,

We are very grateful for the referees' comments regarding our manuscript. You provided valuable comments and thoughtful suggestions, all of which were very helpful for revising and improving our manuscript. Based on your comments and suggestions, we performed further experiments and made careful modifications to our manuscript during the second revision. We hope that the revised manuscript is more acceptable and satisfactory. The main changes are highlighted in yellow in the revised version. Detailed descriptions of the revisions and responses to the reviewers are provided below. During the COVID-19 pandemic, we wish you good health.

Reviewer #1 (Remarks to the Author):

I think the authors have made substantial improvement to the work and the manuscript. There are still two points need to be solved.

Comment-1. For comment-5 (on page 4 of Response to Referees Letter): the author need to perform extra measurement of IAA and the transcription of the related genes using the complimentary lines.

Response: Thank you for this constructive comment. During the COVID-19 pandemic, suspension of business occurred in the company which we selected to measure the IAA level. In addition, IAA standard is not available during COVID-19 pandemic, as a result, we could not perform measurement of IAA by ourselves. However, according to your comment, we examined the expression levels of IAA related genes, and found that expression levels of auxin related genes in the complementary line *gGSA1*^{com} seedlings were comparable to that of NIL-*GSA1*^{WYJ} (described in the revised manuscript line 280-282 and new **Supplementary Fig. 9q**). These data suggest that the complementary line were comparable to NIL-*GSA1*^{WYJ}

with respect to auxin biosynthesis, transport and signaling. Thank you for your kindly understanding and approval.

Comment-2. For comment-6 (on page 5 of Response to Referees Letter): the Kms measured by the authors are several orders different to the published flavonoid UGTs. And it seems that the two UGTs show very similar Kms from the data provided by the authors, which is not in line with the conclusion drawn by the authors. I think the authors need to redo this experiment to give more reliable data.

Response: Thank you for pointing this out. It is our negligence that regarding the kaempferol concentration of mother solution as the concentration of reaction mixture. The concentration of kaempferol of mother solution is 200 times that of reaction mixture. Thus, the *Km*s that we measured were actually in μM instead of mM. We have modified the data according to the correct concentration (new **Supplementary Fig. 11e**). $GSA1^{WYJ}$ and $GSA1^{CG14}$ show similar *Km*s towards kaempferol, implying that $GSA1^{WYJ}$ and $GSA1^{CG14}$ have similar affinity towards kaempferol. We then investigated the affinity of $GSA1^{WYJ}$ and $GSA1^{CG14}$ towards UDP-glucose, and found that the *K*m values of $GSA1^{WYJ}$ and $GSA1^{CG14}$ towards UDP-glucose were 51.06 ± 7.814 μM and 134.6 ± 21.63 μM, respectively (described in the revised manuscript line 427-433 and new **Supplementary Fig. 11f**). These data imply that $GSA1^{WYJ}$ has higher affinity towards UDP-glucose than $GSA1^{CG14}$ and the natural variations in $GSA1^{CG14}$ might result in impaired UDP-glucose binding, which possibly leads to the decreased glucosyltransferase activity of $GSA1^{CG14}$. Thank you for your valuable comments again.

Reviewer #2 (Remarks to the Author):

The authors have addressed my questions and concerns in the revision.

In the revision, can that author include information in the methods regarding the alignments, phylogenetic analyses, and tree building (Figure S4)?

Wendy Peer

Response:

Thank you for pointing this out. As suggested, we described the methods regarding the alignments, phylogenetic analyses, and tree building in Lines 884-890.

Reviewer #3 (Remarks to the Author):

The authors conducted additional experiments and revised the relevant statements appropriately. I believe this manuscript provided valuable scientific and useful agricultural information.
Minor points:
Comment-1. GSA1 explained approximately 14% phenotype variation of grain size (Supplementary Table 1). So, this QTL did not a minor QTL.

Response:
Thank you for pointing this out. We have replaced minor QTL by QTL and deleted the description of minor QTL.

Comment-2. The authors obtained three independent recombinant plants of R3 type and two recombinant plants of R2 type. I suggest the authors to provide the phenotype data of each independent recombinant plant in Figure 1f and Supplementary Figure 3.

Response:
Thank you for the thoughtful suggestion. After identification of the recombinant plants four years ago, we selected representative recombinant plants for fine mapping of GSA1. Thus, in the second revised manuscript, we added "representative recombinant line" in the legends of Figure 1f and Supplementary Figure 3, respectively (please see Lines 175, 1108 and 1260). However, the seeds of other recombinant plant lines are no longer able to germinate at present because they have been stored for at least four years after harvest, therefor we could not get phenotype data of these independent recombinant plants. Thank you for your kindly understanding.

Comment-3. In main text and Supplementary Figure 6, "O. japonica" should be "japonica" or "O. sativa ssp. japonica", and "O. indica" should be "indica" or "O. sativa ssp. indica". In addition, in Supplementary Fig 6 b and c, "O. rufipogen" should be "O. rufipogon".

Response:
Thank you for your kind reminder. It is our negligence. We examined the lines carefully and corrected this mistake in Lines 155, 159, 161, 163, 165, 1283 and 1284, and Supplementary Fig 6 b and c.

Comment-4. Recommend editing for English grammar and usage to improve the readability of this paper.

Response: Thank you for your comment. We have carefully corrected the grammatical errors as you have suggested. We believe that the corrected version reads much better now. Please see the revised manuscript.

REVIEWERS' COMMENTS:

Reviewer #1 (Remarks to the Author):

I think the authors have addressed my concerns.

Third Revision of Manuscript

Reviewer #1 (Remarks to the Author):

I think the authors have addressed my concerns.
